# DNDI-6148: A Novel Benzoxaborole Preclinical Candidate for the Treatment of Visceral Leishmaniasis

**DOI:** 10.1021/acs.jmedchem.1c01437

**Published:** 2021-10-29

**Authors:** Charles E. Mowbray, Stéphanie Braillard, Paul A. Glossop, Gavin A. Whitlock, Robert T. Jacobs, Jason Speake, Bharathi Pandi, Bakela Nare, Louis Maes, Vanessa Yardley, Yvonne Freund, Richard J. Wall, Sandra Carvalho, Davide Bello, Magali Van den Kerkhof, Guy Caljon, Ian H. Gilbert, Victoriano Corpas-Lopez, Iva Lukac, Stephen Patterson, Fabio Zuccotto, Susan Wyllie

**Affiliations:** † Drugs for Neglected Diseases initiative (DNDi), 15 Chemin Louis-Dunant, 1202 Geneva, Switzerland; ‡ Sandexis Medicinal Chemistry Ltd, Innovation House, Discovery Park, Ramsgate Road, Sandwich, Kent CT13 9ND, U.K.; § Scynexis, 3501 C Tricenter Boulevard, Durham, North Carolina 27713, United States; ∥ Laboratory for Microbiology, Parasitology and Hygiene (LMPH), University of Antwerp, Universiteitsplein 1, 2610 Wilrijk, Antwerp, Belgium; ⊥ Faculty of Infectious and Tropical Diseases, London School of Hygiene & Tropical Medicine, Keppel Street, London WC1E 7HT, U.K.; # Anacor Pharmaceuticals, 1020 East Meadow Circle, Palo Alto, California 94303, United States; ∇ Division of Biological Chemistry and Drug Discovery, Wellcome Centre for Anti-infectives Research, School of Life Sciences, University of Dundee, Dow Street, Dundee DD1 5EH, U.K.

## Abstract

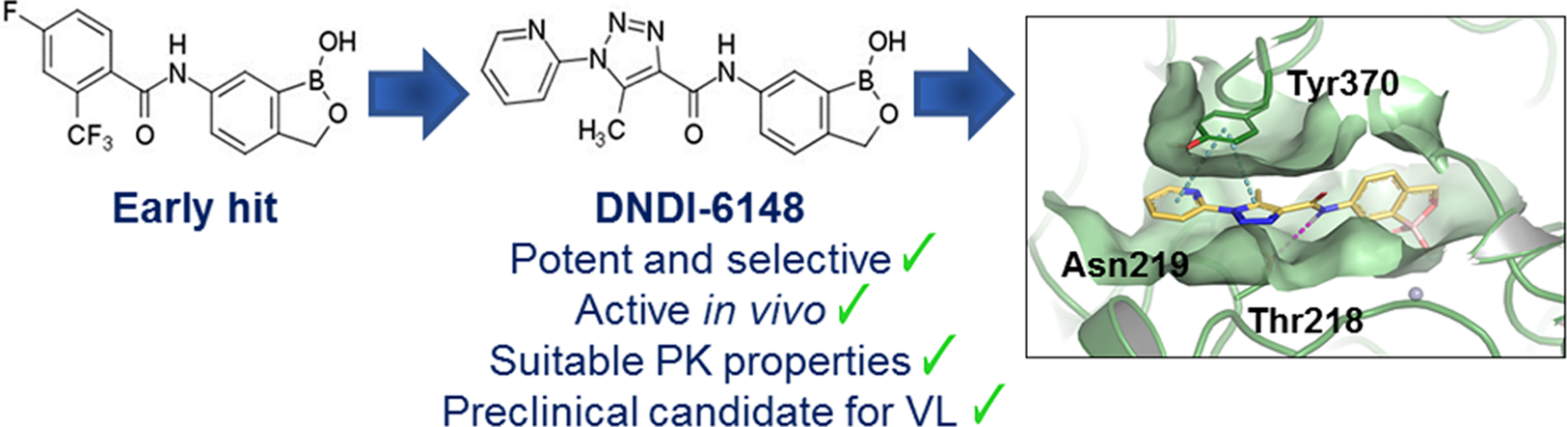

Visceral leishmaniasis (VL) is a parasitic disease endemic across multiple regions of the world and is fatal if untreated. Current therapies are unsuitable, and there is an urgent need for safe, short-course, and low-cost oral treatments to combat this neglected disease. The benzoxaborole chemotype has previously delivered clinical candidates for the treatment of other parasitic diseases. Here, we describe the development and optimization of this series, leading to the identification of compounds with potent *in vitro* and *in vivo* antileishmanial activity. The lead compound (DNDI-6148) combines impressive *in vivo* efficacy (>98% reduction in parasite burden) with pharmaceutical properties suitable for onward development and an acceptable safety profile. Detailed mode of action studies confirm that DNDI-6148 acts principally through the inhibition of *Leishmania* cleavage and polyadenylation specificity factor (CPSF3) endonuclease. As a result of these studies and its promising profile, DNDI-6148 has been declared a preclinical candidate for the treatment of VL.

## Introduction

Visceral leishmaniasis (VL) is a poverty-linked disease,^1^ and occurs primarily in poor populations across Asia, East Africa, and South America.^2^ Current estimates suggest 50 000–90 000 new cases of VL occur each year.^3^ VL is caused by infection with the protozoan parasites *Leishmania donovani* and *Leishmania infantum*, with transmission mediated by the bite of the female phlebotomine sand fly. Once the human host is infected, parasites survive and multiply within macrophages, leading to symptoms including prolonged fever, enlarged spleen and liver, substantial weight loss, and progressive anemia.^4^ If left untreated, the vast majority of clinically symptomatic VL patients die within months.

The current standard treatment for patients with VL in East Africa is sodium stibogluconate (SSG) combined with paromomycin (PM).^4^ In South-East Asia, the standard treatment is liposomal amphotericin B (LAB), with paromomycin and miltefosine as a second-line treatment option.^5^ While these combination approaches are more effective than SSG monotherapy, the mainstay of VL treatment for many years, they still have significant limitations such as cost, route of administration (all given via the parenteral route apart from the oral drug miltefosine), and toxicity. Consequently, there is an ongoing need for effective new treatments that are easy to administer via oral dosing, are effective against VL in different regions of the world, have an improved safety profile, and are affordable for the patients who require them. Research and development for the treatment of VL has evolved rapidly in recent years, with 5 drug candidates from 4 different classes now in phase-1 clinical studies (Table 1).^6^

**Table 1:** Current Portfolio of VL Drug Candidates in Clinical Development^a^

compound ID	compound class	mechanism of action and/or molecular target	organization
DNDI-6148^6^	benzoxaborole	CPSF3	DND*i*/Pfizer
DNDI-0690^6^	nitroimidazole	bioactivation by NTR2^7^	DND*i*/TB Alliance
GSK3186899/DDD853651^6,7^	pyrazolopyrimidine	CRK12 (cyclin-dependent kinase)^8^	GSK/DDU
GSK3494245/DDD1305143^6,8^	imidazopyridine	proteasome inhibitor^9^	GSK/DDU
LXE408^9^	triazolopyrimidine	proteasome inhibitor^10^	Novartis

a DDU: Drug Discovery Unit, University of Dundee and GSK: GlaxoSmithKline.

**Figure 1:**
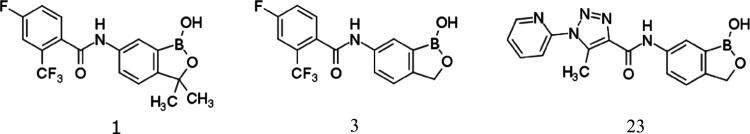
Structures of acoziborole (**1**), early hit 3, and preclinical candidate DNDI-6148 (**23**).

Here, we outline the optimization of a benzoxaborole series toward a possible new therapy for VL. The benzoxaborole class of compounds has already successfully delivered a clinical candidate (acoziborole, AN5568, **1**, Figure 1) for the treatment of stage 1 and stage 2 human African trypanosomiasis (HAT, African sleeping sickness).^11^ Stimulated by the success of the acoziborole program, modifications to the benzoxaborole template were undertaken to identify novel compounds that may have utility for the treatment of VL, leading to the discovery of a preclinical candidate **23** (DNDI-6148). This compound combines good *in vitro* potency against both *L. infantum* and *L. donovani* alongside very high levels of *in vivo* efficacy (>98% reduction in parasite burden) in hamster models of infection. In addition, DNDI-6148 possesses excellent pharmacokinetics and is predicted to have acceptable human pharmacokinetics and an efficacious dose consistent with the requirements of the published target candidate (TCP) and target product (TPP) profiles for VL published by DND*i*. Clinical trials with DNDI-6148 are now underway.^11^

## Results and Discussion

### Chemistry

**Scheme 1:**
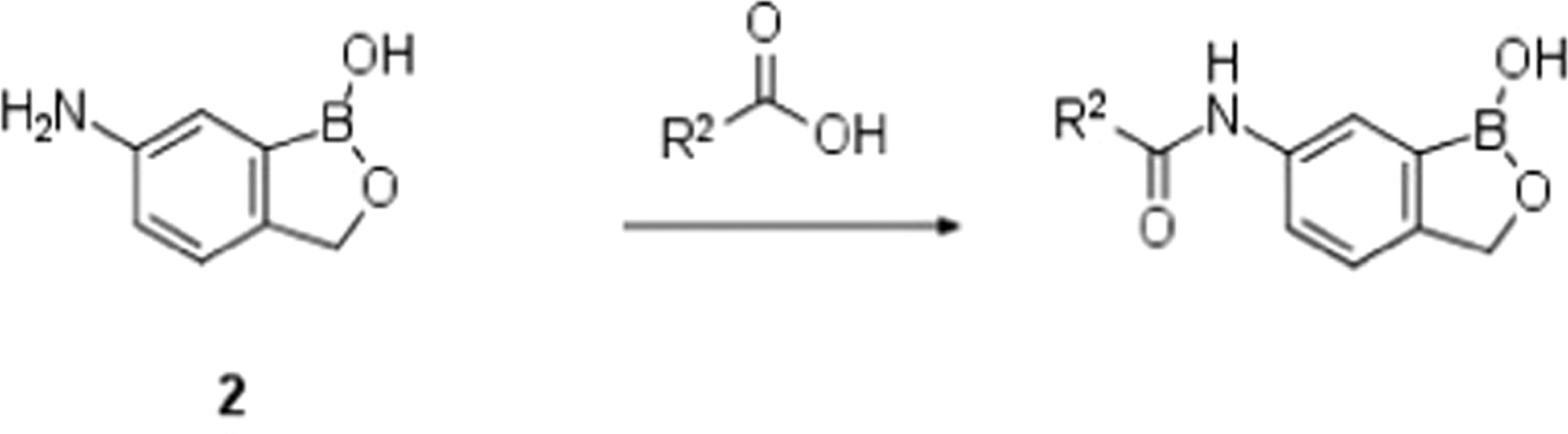
General Synthesis of Amides **4**–**31** Reagents and conditions: Method A: HOBt, EDCI, *N*,*N*-diisopropylethylamine (DIPEA), CH_2_Cl_2_, rt, 2 h, 66%; Method B: HATU, DIPEA, *N*,*N*-dimethylformamide (DMF), rt, 16 h, 8–77%; and Method C: (i) SOCl_2_, 50 °C, 4 h, (ii) DIPEA, tetrahydrofuran (THF), rt, 16 h, 4–68%.

The synthesis of benzoxaborole target compounds **3**–**31** consisted of amide bond formation between the key intermediate **2** and an appropriately functionalized acid (Scheme 1) using one of three coupling methods, whose choice was driven by substrate compatibility: Method A (HOBt/EDCI coupling), Method B (HATU coupling), and Method C (acid chloride formation using SOCl_2_ followed by coupling with intermediate **2**). The synthesis of the carboxylic acid intermediates R^2^CO_2_H is described in detail in the Supplementary Information. Example **12** was synthesized using Method A. Examples **4**, **6**–**11**, **13**–**15**, **17**–**18**, **20**–**21**, **23**–**27**, and **29**–**30** were synthesized using Method B, while examples **5**, **16**, **19**, **22**, **28**, and **31** were synthesized using Method C.

**Scheme 2:**
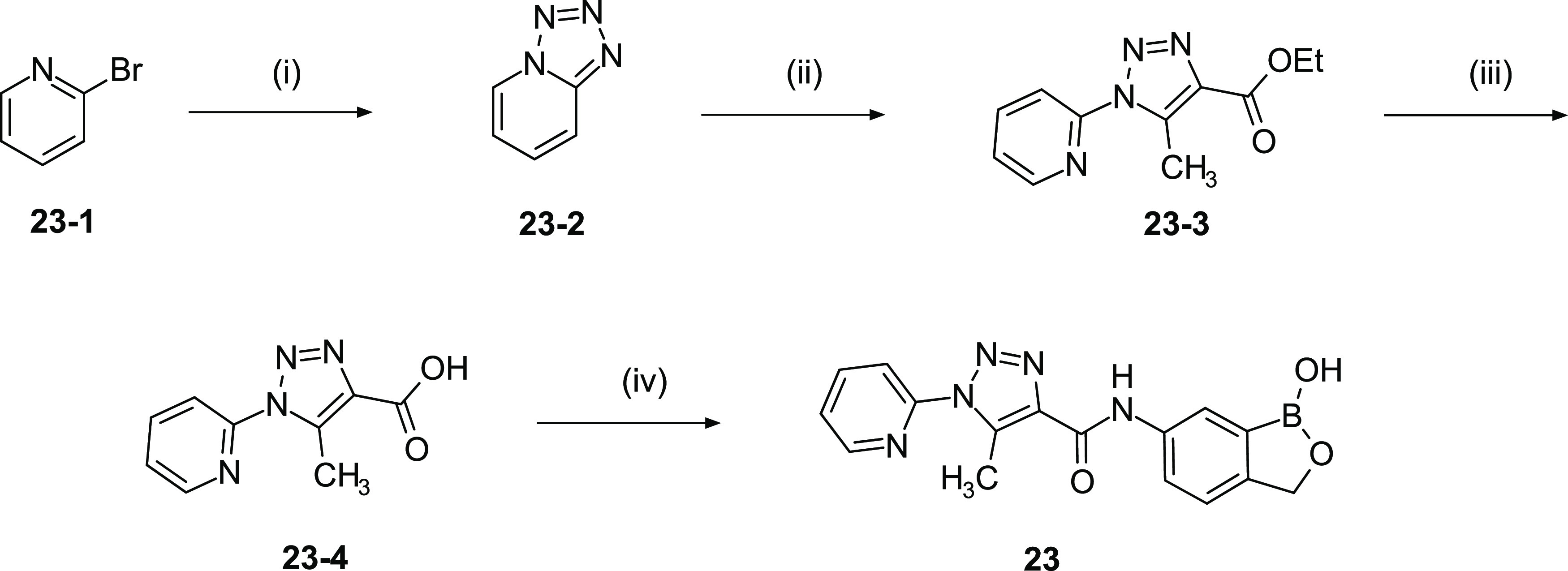
Synthesis of Compound **23** Reagents and conditions: (i) NaN_3_, CuI, NaOAc, DMEDA, EtOH, H_2_O, 1 h, reflux, 94%; (ii) ethyl 3-oxobutanoate, NaOEt, EtOH, 48 h, reflux; (iii) NaOH, H_2_O, EtOH, 20 h, 45 °C, 48% for (ii) and (iii); and (iv) **2**, HATU, DIPEA, DMF, room temperature (RT), 3 h, 74%. Note, the material subsequently used in the mode of action studies was prepared from commercially available **23-2** via a modification of this route (see Scheme 5).

Preclinical candidate compound **23** was synthesized using a 4-step synthesis (Scheme 2).

Formation of the fused tetrazole **23-2** was achieved by the reaction of 2-bromopyridine with sodium azide. Cyclo-addition of **23-2** with ethyl 3-oxobutanoate gave the N1-substituted triazole **23-3**, which was hydrolyzed to the corresponding acid **23-4**. Coupling benzoxaborole scaffold **2** using Method B gave the final compound **23** in high overall yield.

Compound **1** was potent in *in vitro* assays against *Trypanosoma brucei*, the etiological agent of HAT (EC_50_ value of 795 nM),^12^ but demonstrated only weak activity in intramacrophage assays with both *L. infantum* and *L. donovani* (Table 2). It should be noted that *L. infantum*-derived infections can be refractory to some current drug therapies. Thus, new compounds in development are screened against both *L. infantum* and *L. donovani* at an early stage to ensure efficacy. However, the close analogue **3** (SCYX-6759), lacking the gem-dimethyl substitution on the benzoxaborole ring, was considerably more potent in these assays.^13^

**Table 2:** Collated EC_50_ Values for Compounds **1**, **3** and the Current Front-Line Antileishmanial Miltefosine

	EC_50_ values, μM^a^		
compound	intramacrophage *L. donovani*	intramacrophage *L. infantum*	PMM
**1**	59	>64	>64
**3**	1.2	2.7	>64
**23**	1.4	1.8	>64
miltefosine	10	10	33

a Geometric mean value of at least three independent assays. PMM: primary mouse macrophages.

Previous studies demonstrated that compound **3** has low clearance in multiple animal models and is efficacious in a murine model of HAT.^13^ Consequently, compound **3** was progressed to an *L. infantum* hamster model of VL infection (Table 3).^14^ Compound **3** was highly efficacious at QD doses of 50 and 100 mg/kg, virtually clearing all parasites from the liver and spleen. However, reductions in parasite burden in the bone marrow were more modest and thus failed to meet the DND*i* target candidate profile (TCP) of >95% reduction in parasite burden. Therefore, additional benzoxaboroles were synthesized to identify compounds that were highly efficacious in all tissues across a broad dose range.

**Table 3:** *In Vivo* Efficacy of Compound **3** in an *L. infantum* Hamster Model of VL, after QD Dosing for 5 Days^a^

	reduction in parasite burden, %		
dose (mg/kg)	liver	spleen	bone marrow
25	87.8	81.8	81.6
50	97.4	95.0	91.2
100	98.6	96.0	88.3

a *n* = 6 animals per group, results were expressed as a percentage reduction in amastigote burden compared to vehicle-treated, infected control animals.

The simplified benzoxaborole ring system, with the gem-dimethyl substitution on the benzoxaborole ring removed, was retained in subsequent analogues and the amide substituent became the focus for development. Replacing the lipophilic aryl substituent with a more polar heteroaryl group was investigated in detail to reduce the lipophilicity to values more associated with orally bioavailable drugs.^15^ Consistent with this design strategy, a range of substituted pyrazoles was synthesized (Table 4). Excellent levels of *in vitro* potency were achieved with compounds **4**–**6**, which contain a trifluoromethyl R^1^ substituent and a fluorinated *N*-alkyl R^2^ substituent. Importantly, compounds **4–6** showed no evidence of cytotoxicity (CC_50_ > 38 μM) in the counter-screen with PMM. These analogues also had excellent *in vitro* metabolic stability in HLM, but compounds **4** and **6** had poor stability in hamster liver microsomes (HamLM), which precluded their evaluation in the *in vivo* hamster model of VL. Compound **5** combined sufficient *in vitro* potency, selectivity index (SI), and stability in both HLM and HamLM to be considered for further progression. Compound **7** (R^1^ = Me) was slightly less potent than its CF_3_ analogue **6**, and this same trend was also observed for the *N*-cyclobutyl examples **8** and **9** as well as for the 2-pyridyl examples **11** and **12**. However, the CF_3_-substituted analogues **6**, **9**, and **12** were all more cytotoxic in PMM than their CH_3_-substituted counterparts **7**, **8**, and **11**. Tetrahydropyranyl-substituted analogue **10** combined many of the necessary properties, but HamLM stability was insufficient to deliver enough *in vivo* exposure for efficacy studies. Typically, a HamLM clearance of <100 μL/min/mg is required to observe meaningful exposure *in vivo*. Further lipophilic aryl R^2^ substituents such as 4-Cl-phenyl **13** and 2-MeO-phenyl **14** had potent *in vitroL. infantum* activity but were cytotoxic in PMM and/or MRC5 cells. The pyrazole **15** exhibited a significant drop in potency compared to its isomeric analogue **6**, whereas the pyridyl-substituted compound **16** was slightly more potent than its isomer **11**. Further substituted pyridine examples **17** and **18** were also potent and demonstrated good SI. In addition, compounds **16**–**18** were stable in both HLM and HamLM. Additional pyrazole isomers **19**–**21** all failed to combine sufficient *in vitro* potency and weak cytotoxicity with good metabolic stability.

**Table 4:**
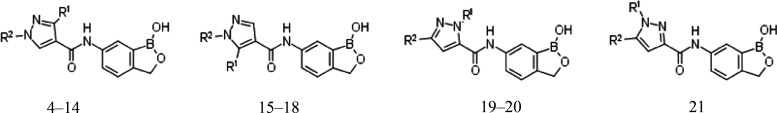
Antileishmanial Activity of Pyrazoles **4**–**21**

					MRC5^a^		HLM	HamLM
compound	R^1^	R^2^	intramacrophage *L. infantum* EC_50_ (μM)^a^	PMM^a^ CC_50_ (μM)	CC_50_ (μM)	SI^b^	Cl_int_ (μL/min/mg)	
**3**			2.7	>64	>64	>24	0.8	n.d.
**4**	CF_3_	CF_3_	2.1	>64	>64	>31	<11.9	1,035
**5**	CF_3_	CH_2_CF_3_	1.6	50	>64	>40	<11.9	41
**6**	CF_3_	(CH_2_)_2_CF_3_	1.3	38	49	38	<11.9	114
**7**	CH_3_	(CH_2_)_2_CF_3_	5.7	>64	>64	>11	n.d.	n.d.
**8**	CH_3_	c-butyl	16	>64	>64	>4	<11.9	57
**9**	CF_3_	c-butyl	0.59	32	26	53	<11.9	337
**10**	CF_3_	4-THP	3.0	>64	>64	>21	<11.9	109
**11**	CH_3_	2-pyridyl	5.7	>64	>64	>11	<11.9	n.d.
**12**	CF_3_	2-pyridyl	0.64	2.0	1.1	1.6	n.d.	n.d.
**13**	CF_3_	4-Cl-phenyl	1.3	8.0	2.6	2	n.d.	n.d.
**14**	CH_3_	2-MeO-phenyl	2.2	>64	15.7	7.3	n.d.	n.d.
**15**	CH_3_	(CH_2_)_2_CF_3_	14	>64	>64	>5	<11.9	48
**16**	CH_3_	2-pyridyl	3.1	59	61	15	<11.9	57
**17**	CH_3_	2-pyridyl-6-OMe	1.4	>64	40	31	<11.9	40
**18**	CH_3_	2-pyridyl-6-Me	1.6	>64	41	25	<11.9	44
**19**	H	2-pyridyl	0.5	32	4.0	8	<11.9	253
**20**	CH_3_	2-pyridyl	34	>64	>64	>2	18	121
**21**	CH_3_	2-pyridyl	1.8	>64	54	31	14	106
miltefosine			10	33	>64	>6.2	n.d.	n.d.

a Geometric mean value of at least two independent tests.

b Selectivity index representing the ratio of CC_50_/EC_50_; data are presented as the geometric mean value of ratios calculated using separate data from at least two independent tests. n.d., not determined.

Of the compounds made up to this point, 2-pyridyl-substituted analogue **16** was one of the most interesting as it combined good *in vitro* potency, low lipophilicity, promising SI, and was stable in both HLM and HamLM. For the next set of compounds, the 2-pyridyl substituent was retained and further changes to the 5-membered heterocycle were investigated (Table 5). Triazole **22**, where R^1^ = H, showed an encouraging improvement in both *in vitro* potency and HamLM stability over pyrazole **16**. The Me-substituted example **23** had an even better profile with high levels of *L. infantum* potency, no sign of cytotoxicity in PMM and MRC5, and very high levels of metabolic stability in HLM and HamLM. Further 1,2,3-triazoles demonstrated that the 2-pyridyl substituent was important; for example, the phenyl example **24** and 3-pyridyl example **27** had significantly weaker *in vitro* potency. Substitution on the 2-pyridyl group could be tolerated, with the 6-OMe analogue **25** showing an improved potency profile but at the expense of metabolic stability. The 6-Me compound **26** lost some potency and was therefore not progressed to metabolic stability assessment. Furan example **28** was very potent, and although it did exhibit some cytotoxicity in both PMM and MRC5, the high level of potency meant that **28** still retained a high SI. Despite being more lipophilic, metabolic stability was retained in both HLM and HamLM and, therefore, **28** was considered for further progression. The isomeric furan **29** had slightly reduced potency and SI compared to **28**, and the thiophene **30** was highly cytotoxic in MRC5 cells. The more polar oxadiazole **31** delivered a good overall profile and was also considered for further progression.

**Table 5:**
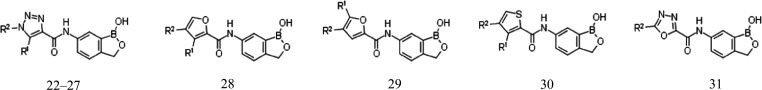
Antileishmanial Activity of Compounds **22–31**

					MRC5^a^		HLM	HamLM
compound	R^1^	R^2^	intramacrophage *L. infantum* amastigote EC_50_ (μM)^a^	PMM^a^ CC_50_ (μM)	CC_50_ (μM)	SI^b^	Cl_int_ (μL/min/mg)	
**22**	H	2-pyridyl	1.7	>64	44.8	30	<11.9	18
**23**	CH_3_	2-pyridyl	1.8	>64	>64	>39	<11.9	<11.9
**24**	CH_3_	phenyl	37	>64	>64	>1.7	<11.9	n.d.
**25**	CH_3_	2-pyridyl-6-OMe	0.8	32	>64	>80	23	40
**26**	CH_3_	2-pyridyl-6-Me	6.4	>64	>64	>10	n.d.	n.d.
**27**	CH_3_	3-pyridyl	38	>64	>64	>1.8	n.d.	n.d.
**28**	CH_3_	2-pyridyl	0.5	49	18	37	<11.9	40
**29**	CH_3_	2-pyridyl	1.7	29	45	18	<11.9	82
**30**	CH_3_	2-pyridyl	0.6	32	2.3	4	<11.9	88
**31**		2-pyridyl	2.0	>64	>64	>32	19	12

a Geometric mean value of at least two independent tests.

b Selectivity index representing the ratio of CC_50_/EC_50_; data are presented as the geometric mean value of ratios calculated using separate data from at least two independent tests. n.d., not determined.

The most promising compounds from this SAR investigation, namely **5**, **16**, **23**, **28**, and **31**, were also assessed for their *in vitro* macrophage potency using intracellular *L. donovani* (Table 6). Pleasingly, four of these compounds retained excellent levels of *L. donovani* potency; however, the oxadiazole **31** was about 5-fold weaker against *L. donovani* compared with its *L. infantum* potency.

**Table 6:** Antileishmanial Activity of Selected Compounds against *L. donovani*

				MRC5^a^	
compound	intramacrophage *L. infantum* EC_50_ (μM)^a^	intramacrophage *L. donovani* EC_50_ (μM)^a^	PMM^a^ CC_50_ (μM)	CC_50_ (μM)	SI^b^
**5**	1.6	1.0	53.8	>64	>64
**16**	3.1	1.2	>64	61.1	52
**23**	1.8	1.4	>64	>64	>46
**28**	0.5	0.4	54	18	45
**31**	2.0	9.4	>64	>64	>7

a Geometric mean value of at least two independent tests.

b Selectivity index representing the ratio of CC_50_/EC_50_; data are presented as the geometric mean value of ratios calculated using separate data from at least two independent tests.

Compounds **5**, **16**, **23**, **28**, and **31** were also progressed to hamster oral pharmacokinetic (PK) studies and an *L. infantum* model of VL in hamsters (Table 7). Triazole **23** exhibited noticeably higher exposure after a single oral dose of 50 mg/kg compared with the other lead compounds, which is consistent with its higher *in vitro* stability in HamLM and indicated that *in vitro* metabolic clearance may be predictive of *in vivo* clearance for this set of compounds.

**Table 7:** Oral Exposure and *In Vivo* Efficacy of Selected Compounds in an *L. infantum* Hamster Model of VL Following BID Dosing for 5 and 10 Days^a^

		reduction in parasite burden, %						
		liver		spleen		bone marrow		
compound	dose (mg/kg), BID	5 days	10 days	5 days	10 days	5 days	10 days	AUC_0–24_ after single p.o. dose of 50 mg/kg (h.ng/mL)
**5**	25	89.7	n.d.	90.0	n.d.	77.0	n.d.	21 000
	50	99.7	n.d.	99.2	n.d.	98.0	n.d.	
**16**	25	87.8	n.d.	87.7	n.d.	72.2	n.d.	7900*
	50	99.6	n.d.	98.5	n.d.	95.1	n.d.	
**23**	25	98.3	100	98.2	99.9	93.6	99.4	120 000
	50	99.8	n.d.	99.9	n.d.	99.0	n.d.	
**28**	25	98.9	99.4	97.8	96.9	95.7	84	21 000
	50	99.9	n.d.	99.9	n.d.	99.1	n.d.	
**31**	25	67.3	n.d.	69.0	n.d.	55.0	n.d.	21 000
	50	99.4	n.d.	99.4	n.d.	99.7	n.d.	

a *n* = 6 animals per group, the results are expressed as a percentage reduction in amastigote burden compared to vehicle-treated, infected control animals, *Study run using a single 25 mg/kg p.o. dose.

All five compounds demonstrated excellent efficacy, with parasitemia significantly reduced in all organs after a dose of 50 mg/kg BID for 5 days. Triazole **23** and furan **28** also maintained excellent efficacy at a lower dosing of 25 mg/kg BID, consistent with the combination of good potency and high exposure observed for **23** and the very high potency of **28**. Extending the duration of treatment from 5 to 10 days for compound **23** led to almost complete eradication of parasites in the liver, spleen, and bone marrow. In contrast, the efficacy profile of compound **28** was not improved by extending the dosing period.

Compounds **23** and **28** were then studied in an *L. donovani* hamster model (Table 8). Triazole **23** exhibited high levels of efficacy in the liver and spleen when dosed orally at 50 mg/kg BID for 5 days, although the reduction in parasite load in bone marrow was lower than the target of 95%. However, by extending the dosing period to 10 days, very high efficacy across all three tissues was achieved at a dose of 25 mg/kg BID. In addition, furan **28** achieved excellent efficacy in all tissues at both 25 mg/kg BID and 50 mg/kg BID for 5 days. These important data demonstrated that for both *L. infantum* and *L. donovani*, potent *in vitro* and *in vivo* activity could be achieved.

**Table 8:** *In Vivo* Efficacy of Selected Compounds in an *L. donovani* Hamster Model of VL Following BID Dosing for 5 and 10 Days^a^

		reduction in parasite burden, %					
		liver		spleen		bone marrow	
compound	dose (mg/kg), BID	5 days	10 days	5 days	10 days	5 days	10 days
**23**	25	n.d.	99.9	n.d.	99.8	n.d.	99.6
	50	99.3	n.d.	98.6	n.d.	83.4	n.d.
**28**	25	95.9	n.d.	95.9	n.d.	94.5	n.d.
	50	99.9	n.d.	100	n.d.	100	n.d.

a *n* = 6 animals per group, results were expressed as a percentage reduction in amastigote burden compared to vehicle-treated, infected control animals, n.d., not determined.

Compounds **23** and **28** were also assessed in mouse models of *L. donovani* and *L. infantum*; data confirmed that the high levels of efficacy observed in the chronic hamster model of infection was also achieved in the acute mouse model (Table 9).

**Table 9:** *In Vivo* Efficacy of Selected Compounds in *L. donovani* and *L. infantum* Mouse Models of VL Following BID Dosing for 5 Days

		reduction in liver parasite burden following 5 days of p.o. dosing BID^a^, %	
compound	dose (mg/kg), BID	*L. infantum*	*L. donovani*
**23**	25	96.1	97.1
	50	98.6	99.7
**28**	25	n.d.	97.5
	50	n.d.	99.5

a *n* = 5 animals per group, the results were expressed as a percentage reduction in parasite burden compared to vehicle-treated, infected control animals, n.d., not determined.

Based on their *in vitro* potency, metabolic stability, and *in vivo* pharmacokinetic and efficacy profiles, compounds **23** and **28** were investigated in more detail to determine if either could be progressed to further preclinical development. Encouraged by the outstanding *in vivo* efficacy of compound **28**, a 14-day exploratory toxicology study was conducted in male and female Sprague-Dawley rats. Unfortunately, compound **28** caused significant multiorgan toxicity following daily dosing of 25 mg/kg and the NOAEL (No Observed Adverse Effect Level) was not determined (<12.5 mg/kg). Consequently, further development of compound **28** was halted.

**Scheme 3:**
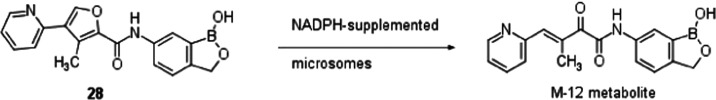
Metabolic Fate of Compound **28** in the Presence of Microsomes from Multiple Species

One potential concern associated with compound **28** was the presence of the furan, a functional group that can undergo oxidative metabolism to reactive metabolites.^16^ Thus, **23** and **28** were investigated to determine potential routes of metabolism and to understand if protein–ligand adducts were formed when the compounds were incubated in microsomes in the presence of glutathione. Metabolite identification studies with compound **28** showed evidence of ring-opening of the furan to an enone M-12 metabolite in all species tested (rat, mouse, dog, human, hamster). This metabolite has the potential to undergo conjugation to form protein–ligand adducts (Scheme 3).

**Scheme 4:**
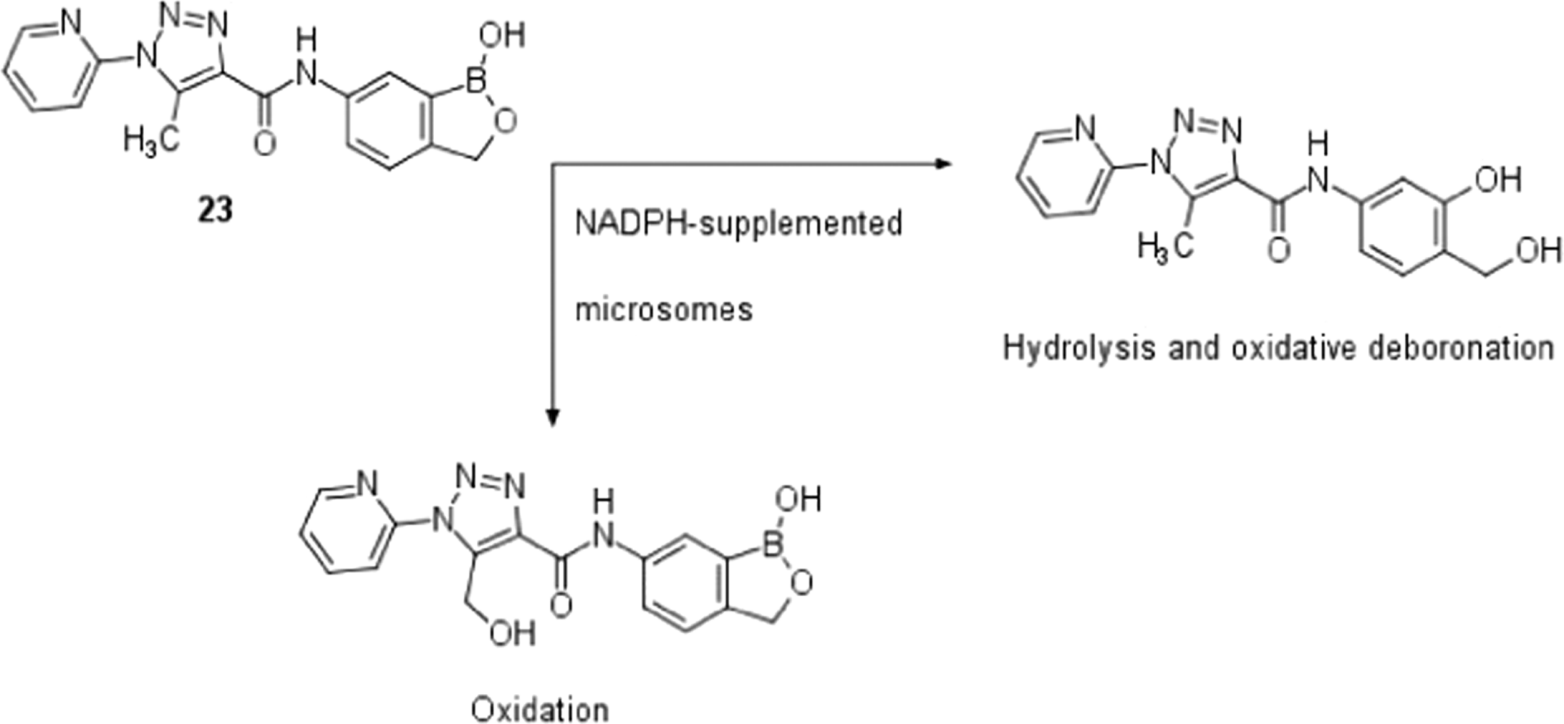
Metabolic Fate of Compound **23** in the Presence of Microsomes

In contrast, incubation of triazole **23** with microsomes indicated very limited metabolism, with only small amounts of mono-oxidation and hydrolysis/oxidative deboronation observed in dog and human microsomes, respectively (Scheme 4). No metabolism was detected in rat microsomes, and no evidence of reactive metabolite formation was found in any species.

In addition to the safety signals raised by a moderate *in vitro* activity against the MRC5 cell line (Table 5) and the lack of efficacy improvement when treatment duration was extended in the hamster model (Table 7), these *in vitro* metabolic studies clearly identified a potential weakness with furan **28** that may contribute to the observed *in vivo* toxicity. Therefore, **23** was prioritized for further evaluation. CYP inhibition was weak (IC_50_ > 50 μM vs CYP1A2, 2C9, 2C19, 3A4, 2D6), hERG selectivity was high (hERG inhibition < 20% at 30 μM), and no activity > 50% was observed when **23** was tested in a panel of 88 targets (Cerep panel, Figure S13) at a concentration of 10 μM. Plasma-protein binding was moderate and consistent across species (dog 87%, human 92%, mouse 93%, rat 92%, hamster 88%). Thermodynamic solubility in physiologically relevant media was low to moderate (Fasted State Simulated Intestinal Fluid, FaSSIF solubility = 5.4 μg/mL, Fed State Simulated Intestinal Fluid, FeSSIF solubility = 6.3 μg/mL), and membrane permeability was good with no evidence of P-gp-mediated efflux (MCDK efflux ratio of 0.7, Table S3). Microsome and hepatocyte stability was excellent across all species (Table 10), although hepatocyte stability was slightly worse in dogs compared with rats, monkeys, and humans. Compound **23** was also negative when tested in an Ames assay (TA98 and TA100 bacterial strains) with and without metabolic activation over a dose range from 1.5 to 1000 μg/well, suggesting no clastogenicity. To complete the early safety profile of compound **23**, a 14-day exploratory toxicology study was conducted in male and female Sprague-Dawley rats where the no adverse effect limit was set at 50 mg/kg/day.

**Table 10:** *In Vitro* Metabolic Stability Across Species for Compound **23**

species	mouse	rat	dog	human
microsome Cl_int_ (μL/min/mg protein)	<11.9	<11.9	5.8	<11.9
hepatocyte Cl_int_ (μL/min/million cells)	n.d.	0.7	2.4	0.5

*In vivo* pharmacokinetics after i.v. and p.o. dosing was determined in rats and dogs (Table 11 and Figure S12). Clearance in rats was low (4.5 mL/min/kg) with comparatively higher clearance in dogs (13.7 mL/min/kg), in line with the higher turnover in dog hepatocytes compared with rat hepatocytes. Oral bioavailability in rats was very high, indicating complete oral absorption. Bioavailability was somewhat lower in dogs and not fully understood at this point, but still acceptable for future progression of the compound. Allometric scaling from available rat and dog PK data suggests that compound **23** will have low clearance (Cl = 1–4 mL/min/kg) and moderate–high bioavailability (40–90%) in humans. BID dosing in humans at 3–20 mg/kg is predicted to achieve the efficacious exposure observed in the *L. infantum* and *L. donovani* hamster and mouse models of VL.

**Table 11:** *In Vivo* Pharmacokinetics of **23** in Rat and Dog

species	rat^a^		dog^b^	
route of administration	i.v.	p.o.	i.v.	p.o.
clearance (mL/min/kg)	4.5		13.7	
*V*_d_ (L/kg)	0.6		1.3	
*T*_1/2_ (h)	2.8		1.5	
*F* (%)		94		39

a *n* = 3 male SD rats, 2 mg/kg i.v., 10 mg/kg p.o.

b *n* = 2 male Beagle dogs, 1 mg/kg i.v., 5 mg/kg p.o.

### Mode of Action Studies (MoA)

MoA studies as an integrated part of a drug discovery program can provide vital information that can be used to combat the high failure rates associated with the development of phenotypically active compounds. The association of active compounds with defined molecular targets during the development process can be extremely powerful. Understanding compound MoA can allow toxic liabilities associated with the target to be directly assessed, prevent enrichment of drug candidates against the same molecular target, and halt the development of inhibitors with an unattractive or invalidated target. Furthermore, this knowledge can inform future drug combination strategies.

**Figure 2:**
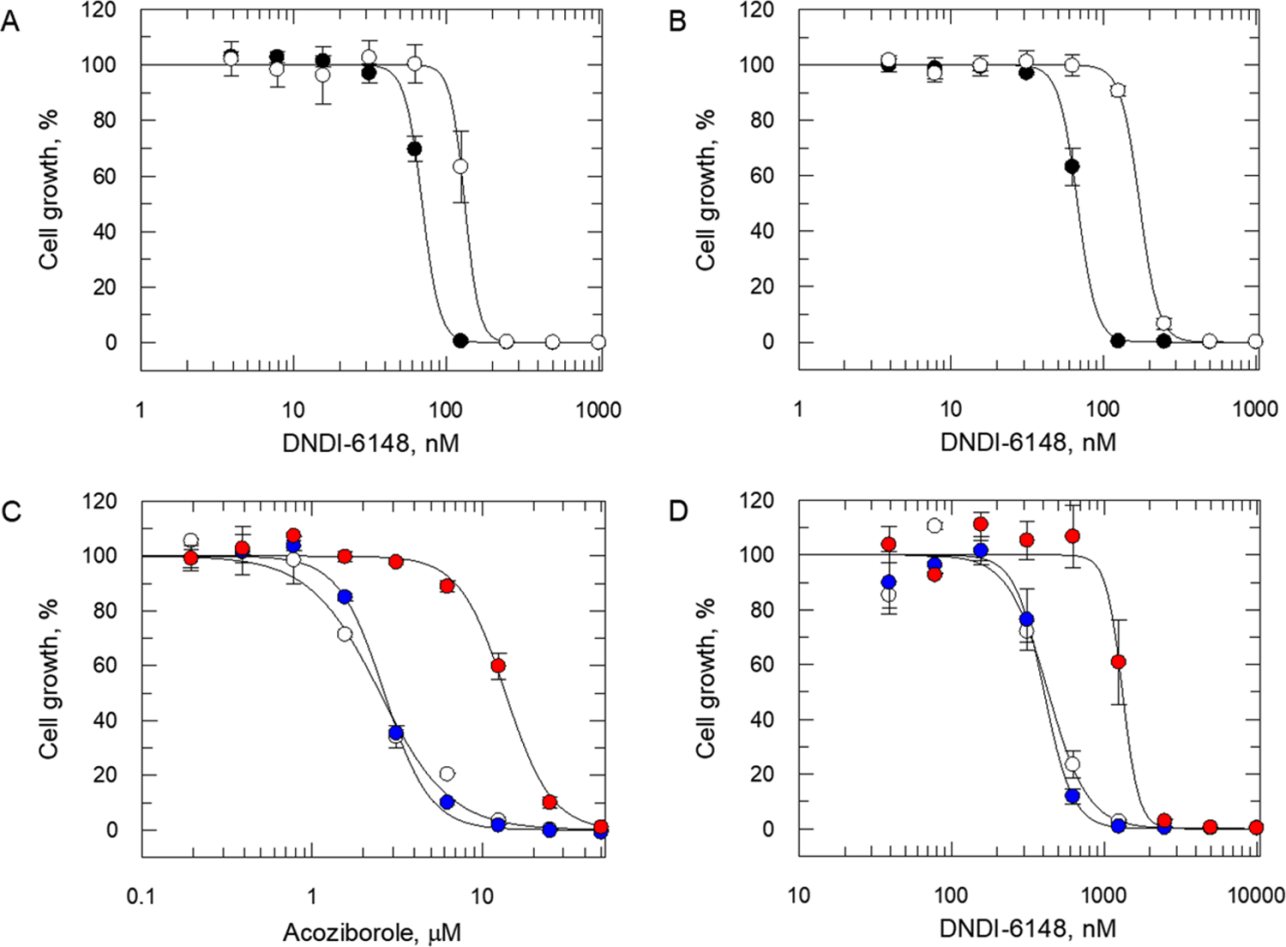
Potency of DNDI-6148 (**23**) against wild-type and transgenic *T. brucei* and *L. donovani*. (A) EC_50_ values for DNDI-6148 (**23**) were determined for wild-type *T. brucei* trypanosomes (closed circles) and *T. brucei* trypanosomes maintaining a mutated version of CPSF3 (Asn^232^His) (open circles). EC_50_ values of 47 ± 0.9 and 90 ± 3.4 nM were determined for wild-type and transgenic trypanosomes, respectively. (B) EC_50_ values of 67 ± 1 and 172 ± 3 nM were determined for DNDI-6148-treated wild-type trypanosomes (closed circles) and trypanosomes overexpressing the wild-type version of CPSF3, respectively. (C) EC_50_ values of 2.5 ± 0.1, 2.8 ± 0.1, and 11.7 ± 0.4 μM were determined for acoziborole-treated wild-type (open circles), CPSF3-overexpressing (blue circles), and CPSF3 (Asn^219^His)-overexpressing (red circles) *L. donovani* promastigotes, respectively. (D) EC_50_ values of 411 ± 12, 508 ± 13, and 1674 ± 51 nM were determined for DNDI-6148-treated wild-type (open circles), CPSF3-overexpressing (blue circles), and CPSF3 (Asn^219^His)-overexpressing (red circles) *L. donovani* promastigotes, respectively. All curves are the nonlinear fits of data using a two-parameter EC_50_ equation provided by GraFit. EC_50_ values are the weighted mean ± standard deviation of at three biological replicates (*n* = 3), with each biological replicate comprised of three technical replicates.

Our previous studies with acoziborole (AN5568, **1**), in clinical development for HAT, revealed that this promising benzoxaborole specifically targets the Cleavage and Polyadenylation Specificity Factor 3 (CPSF3) in *T. brucei*.^17−19^ Additional compounds from within this series were also shown to target CPSF3, including **3**.^13,19^ CPSF3 is an endonuclease that forms part of the CPSF complex, involved in the control of polyadenylation and trans-splicing of pre-mRNA. Indeed, CPSF3 orthologues have also been identified as the molecular targets of benzoxaboroles active against *Plasmodium falciparum,*(20)*Toxoplasma gondii*,^21^ and *Cryptosporidium spp*.^21^ With this in mind, we hypothesized that DNDI-6148 (**23**) might also target this important endonuclease in *L. donovani*. To test this hypothesis, we first assessed the potency of DNDI-6148 (**23**) against *T. brucei* bearing a mutation in the active site of CPSF3 (Asn^232^His), previously demonstrated to confer resistance to acoziborole (**1**).^19^ Bloodstream trypanosomes bearing this specific mutation were 1.8-fold less sensitive to DNDI-6148 (**23**) compared to wild-type parasites (Figure 2A). Similarly, trypanosomes overexpressing the wild-type version of CPSF3 were 2.9-fold less sensitive to DNDI-6148 (**23**) (Figure 2B). Collectively, these data suggest that, like acoziborole (**1**), this benzoxaborole specifically targets CPSF3 in *T. brucei*.

To determine if these boron-containing compounds specifically target CPSF3 in *L. donovani*, transgenic promastigotes were generated overexpressing either CPSF3^WT^ or CPSF3 bearing an Asn^219^His mutation, equivalent to the Asn^232^His mutation in the *T. brucei* enzyme. Elevated levels of the wild-type and mutated CPSF3 in these cell lines were confirmed by label-free quantification (Figure S2). Overexpression of CPSF3^WT^ in *L. donovani* promastigotes did not substantively affect susceptibility to either acoziborole (**1**) (Figure 2C) or DNDI-6148 (**23**) (Figure 2D). However, promastigotes overexpressing the mutated version of this enzyme were significantly less sensitive to both compounds, demonstrating a 5-fold and 3.6-fold reduction in susceptibility to acoziborole (**1**) and DNDI-6148 (**23**), respectively (Figure 2C,D). The shift in potency observed with parasites overexpressing mutated CPSF3 was found to be specific for benzoxaboroles. Compound **3** elicited a similar shift in potency, while the established *N*-myristoyltransferase inhibitor DDD100097^22^ did not (Figure S3). The fact that this single Asn^219^His mutation in the active site of CPSF3 has a marked effect on the potencies of both acoziborole (**1**) and DNDI-6148 (**23**) provides strong evidence that this endonuclease is the molecular target of this preclinical candidate.

### Precision Base Editing of *Ld*CPSF3

We next utilized precision base editing with Cas9 to further probe the interactions between DNDI-6148 (**23**) and *L. donovani* CPSF3. In the first instance, a template encoding a specific Asn^219^His mutation was provided to repair a Cas9-induced lesion within CPSF3. Transfected parasites were then selected with DNDI-6148 (**23**) (6 μM). The resulting DNDI-6148-resistant population was subcloned, genomic DNA was harvested, and the CPSF3 was sequenced to ensure that the desired edit had been successfully introduced. In all cases, these parasites maintained the Asn^219^His encoded by the repair template and were consistently 3-fold less sensitive to DNDI-6148 (**23**) than wild-type (Table S2). As in our previous studies with *T. brucei* CPSF3, attempts to edit Asn^219^ to Tyr to mimic the human CPSF3 enzyme at this position were not tolerated by *L. donovani*. However, using a degenerate repair template, parasites were recovered, maintaining Asn^219^His and also Glu^229^Val homozygous mutations. These doubly mutated parasites were >5-fold less sensitive to DNDI-6148 (**23**) (Table S2). Collectively, these data provide compelling evidence that DNDI-6148 specifically targets the endonuclease CPSF3 in *L. donovani*. These data are also consistent with a recently published study reporting CPSF3 as amongst the top “hits” following the selection of the Cos-Seq genome-wide overexpression library with DNDI-6148.^23^

### Molecular Modeling

**Figure 3:**
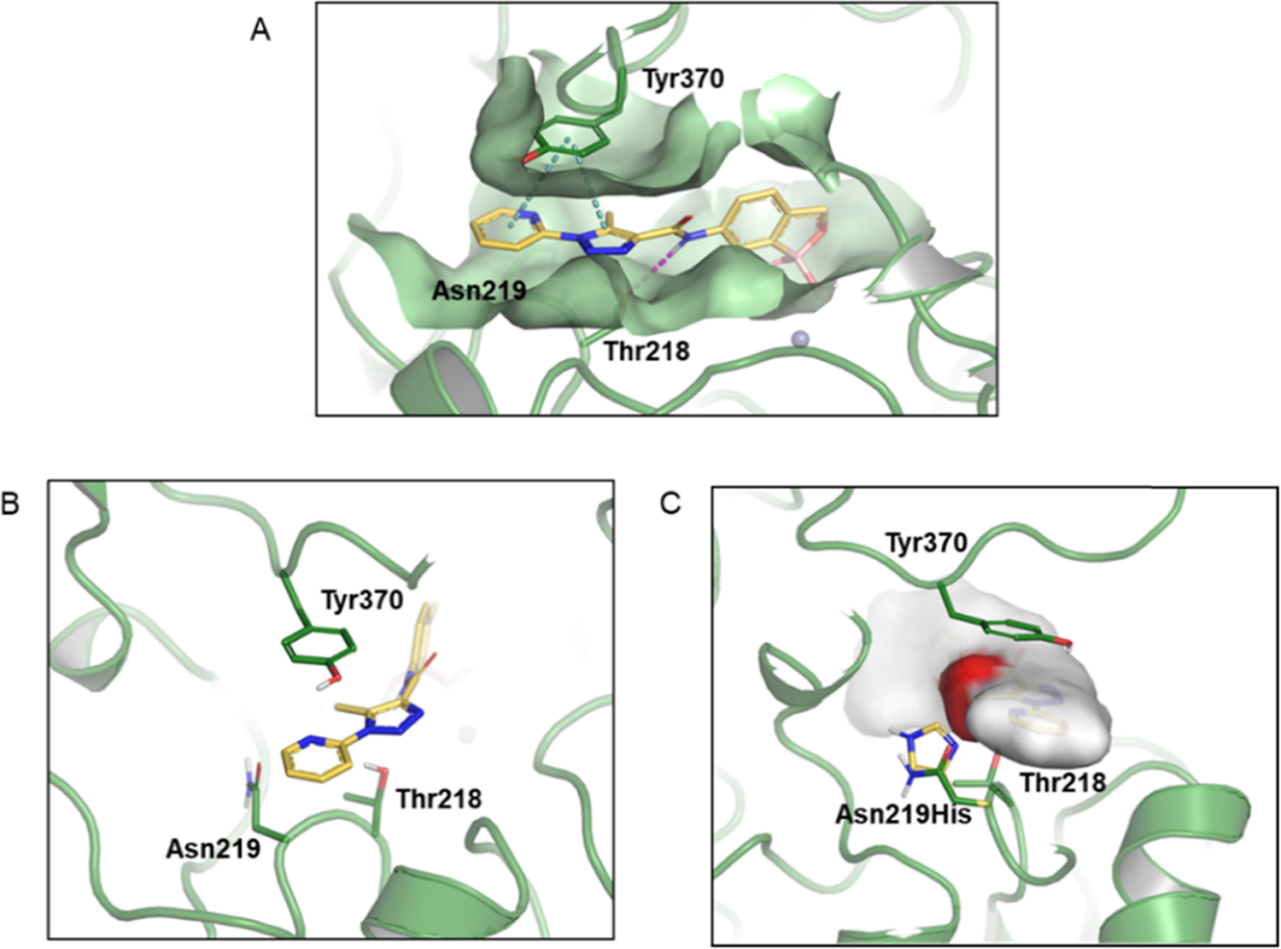
Molecular docking of DNDI-6148 into a model of the *L. donovani* CPSF3 active site. (A) Docking model for DNDI-6148 (**23**) (yellow structure) bound to *L. donovani* CPSF3. Gray spheres represent zinc atoms, blue dotted lines indicate a π stacking interaction, and the purple dotted line indicates a hydrogen bond interaction. (B) Close-up of key interactions involved in DNDI-6148 (**23**) (yellow structure) binding in the active site of CPSF3. (C) Modified docking model illustrating a steric clash between DNDI-6148 (**23**) (red patch) and the His residue (yellow) in Asn^219^His substituted CPSF3. The wild-type CPSF3 structure is shown in green.

A homology model of the *L. donovani* CPSF3 was generated using the crystallographic structure of the *Thermus thermophilus* TTHA0252 homologue as a template (PDB code 3IEM—Figure 3). The *Leishmania* enzyme shares 30% sequence identity with this bacterial homologue. The proposed binding mode for DNDI-6148 (**23**) in the model is consistent with the binding of the benzoxaborole acoziborole to the catalytic site of CPSF3 located at the interface of the metallo-β-lactamase and β-CASP domains in *T. brucei* CPSF3.^19^ The site comprises two zinc atoms coordinated by a network of conserved histidine and aspartic acid residues. Interaction with a zinc-activated water molecule leads to the formation of a negatively charged tetrahedral boronate species that coordinates the zinc atoms (Figure S4), mimicking the transition state of the phosphate of the RNA substrate. The amide in position 6 of the benzoxaborole moiety directs the pyridyl-triazole moiety of DNDI-6148 (**23**) toward the area occupied by the terminal uracil base of the RNA substrate and establishes a π-stacking interaction with Tyr^370^ (Figure 3A). Additionally, the amide NH forms a hydrogen bond with the hydroxyl group in the Thr^218^ side-chain. There seems to be no direct interaction between DNDI-6148 (**23**) and the Asn^219^ residue, where mutation to His is associated with resistance (Figure 3B). However, we propose that mutation to a bulkier His residue in this position has a negative impact on DNDI-6148 (**23**) binding due to steric clashes with the methyl pyridyl-triazole moiety of the compound (Figure 3C). This likely prompts the ligand to adopt a different binding mode where a hydrogen bond with Thr^218^ is lost, the hydrophobic methyl is directed toward the solvent, and the overall ligand conformation is strained. Undoubtedly, there is a high degree of similarity surrounding the proposed binding site of DNDI-6148 in the parasite enzyme and the human homologue, with 21 identical residues out of the 26 within 5 Å from the bound ligand,^19^ However, Asn^219^ in the parasite enzyme is replaced by a tyrosine residue in the human homologue. This bulkier tyrosine residue is likely to cause severe steric hindrance that prevents DNDI-6148 from binding to the human and is entirely consistent with the favorable selective toxicity profile of this compound.

The second mutation (Glu^229^Val) is at the beginning of helix 7 in the β-CASP domain of CPSF3. Helix 7 is some way from the catalytic site but is connected to the loop that contains Thr^218^, Asn^219^, and Ile^221^, defining the pocket that recognizes the pyridine ring of DNDI-6148 (**23**). In our models, a mutation from Glu^229^ to Val would result in a structural rearrangement of the loop, changing the morphology of the binding site and ultimately impacting the ligand recognition event.

## Conclusions

We have optimized a series of benzoxaboroles and identified a range of compounds with very potent *in vitro* antileishmanial activity against *L. infantum* and *L. donovani*, starting from benzamide-substituted compound **3**. Variation of the amide substituent was explored extensively, and a range of heterocyclic amide groups was found to deliver potent *in vitro* activity combined with good metabolic stability. Several compounds were assessed in the *L. infantum* hamster model of VL, with compound **23** standing out by exhibiting very high levels of efficacy in all organs at doses of 25 mg/kg BID for 5 or 10 days. Additional *in vivo* studies in the *L. donovani* hamster model also indicated high levels of efficacy, demonstrating that **23** was equally efficacious against both *Leishmania* species responsible for causing VL. Efficacy of **23** was also confirmed in *L. donovani* and *L. infantum* BALB/c mouse models. Further study of compound **23** indicated that it had excellent pharmacokinetics, a good *in vitro* safety profile, and met all of the criteria in the DND*i* TCP for VL. Comprehensive MoA studies confirm that DNDI-6148 (**23**) targets the endonuclease CPSF3, a previously unexploited drug target in *Leishmania spp*. Consequently, **23** (DNDI-6148) was nominated as a preclinical candidate and further development toward clinical studies is ongoing. Furthermore, compound **23** demonstrates similar levels of *in vitro* and *in vivo* activity against species of *Leishmania* responsible for causing cutaneous leishmaniasis (CL), supporting the development of this candidate for multiple forms of leishmaniasis.^24^ It should be noted that we have also generated data that supports the development of DNDI-6148 for Chagas’ disease. This information will be disclosed in due course. In conclusion, the data package for DNDI-6148 (**23**) provides every reason to believe that this benzoxaborole can become a much-needed safe oral treatment for patients suffering from this devastating neglected tropical disease.

## Experimental Section

### Chemistry

#### Purity

All compounds reported in this study are >95% pure as determined by high-performance liquid chromatography (HPLC) analysis. HPLC chromatograms of a representative compound (**3**) and all analogues used in *in vivo* experiments (**5**, **16**, **23**, **28**, **31**) are shown in the Supplementary Information (Figures S6–S11).

#### General

Unless otherwise indicated, all reactions were magnetically stirred under an inert atmosphere. All reagents, including solvents, were used as received. Anhydrous solvents were dried in-house by passing through activated alumina. Thin-layer chromatography was performed on glass-backed precoated silica gel 60 plates, and compounds were visualized using UV light or iodine. Silica gel column chromatography was performed using 200–300-mesh silica gel. Preparative HPLC was performed using Gilson-281 liquid handlers equipped with one of four columns, chosen from (1) Phenomenex Synergi C18 150 mm × 30 mm, 4 μm, (2) YMC-pack ODS-AQ 150 mm × 30 mm, 5 μm, (3) Agela Venusil ASB C18 150 mm × 21.2 mm, 5 μm, or (4) Boston Symmetrix C18 ODS-R 150 mm × 30 mm, 5 μm; elution was performed with 0.225% (by volume) of formic acid in water (solvent A) and acetonitrile (solvent B); fractions containing products were lyophilized. NMR spectra were recorded on a Bruker AVANCE 400 MHz spectrometer in the solvents specified. Liquid chromatography–mass spectrometry (LC-MS) spectra were recorded on an Agilent 1200 or Shimadzu 2020 spectrometer equipped with electrospray ionization, quadrupole MS detector, and Chromolith Flash RP-18e 25 mm × 2.0 mm column, eluting with 0.0375% (by volume) of trifluoroacetic acid (TFA) in water (solvent A) and 0.01875% (by volume) of TFA in acetonitrile (solvent B). Analytical HPLC was performed using a Shimadzu LC20AB machine and one of two columns, chosen from (1) Xtimate C18 2.1 mm × 30 mm, 3 μm or (2) CHROM-MATRIX Innovation C18 2.1 mm × 30 mm, 2.6 μm; elution was performed with 0.0375% (by volume) of TFA in water (solvent A) and 0.01875% (by volume) of TFA in acetonitrile (solvent B). The purity of final compounds was ≥96%, as determined by HPLC. Unless otherwise stated, final compounds were isolated as amorphous solids without collection of melting point data.

### General Procedures for the Preparation of Amides

#### General Procedure A

To a solution of the corresponding acid (14.4 mmol, 1.0 equiv), HOBt (17.3 mmol, 1.2 equiv), EDCI (17.3 mmol, 1.2 equiv), and DIPEA (36.1, 2.5 equiv) in dichloromethane (DCM) (30 mL) was added 6-aminobenzo[*c*][1,2]oxaborol-1(3*H*)-ol (14.4 mmol, 1.0 equiv). The reaction mixture was stirred at room temperature for 2 h. Water (100 mL) was added, and the reaction mixture was extracted with DCM (100 mL × 3). The combined organic extracts were washed with brine (100 mL × 2), dried with anhydrous Na_2_SO_4_, filtered, and concentrated in a vacuum to give the crude product, which was purified by silica gel chromatography or preparative HPLC.

#### General Procedure B

To a solution of the corresponding acid (1.5 mmol, 1.0 equiv), HATU (1.9 mmol, 1.3 equiv), and DIPEA (2.8 mmol, 1.8 equiv) in DMF (3 mL) was added 6-aminobenzo[*c*][1,2]oxaborol-1(3*H*)-ol (1.5 mmol, 1.0 equiv). The mixture was stirred at room temperature overnight. The mixture was purified by preparative HPLC.

#### General Procedure C

A solution of the corresponding acid (10.5 mmol) in SOCl_2_ (40 mL) was stirred at 50 °C for 4 h. The mixture was concentrated in a vacuum to give acyl chloride, which was used directly in the next step. To a solution of 6-aminobenzo[*c*][1,2]oxaborol-1(3*H*)-ol (1.2 g, 8.1 mmol) and DIPEA (2.9 mL, 16.7 mmol) in THF (30 mL) was added the acid chloride (8.1 mmol). The mixture was stirred at room temperature for 16 h. Water (100 mL) was added, and the reaction mixture was extracted with EtOAc (100 mL × 3). The combined organic extracts were washed with brine (100 mL × 2), dried with anhydrous Na_2_SO_4_, filtered, and concentrated in a vacuum to give the crude product, which was purified by silica gel chromatography or preparative HPLC.

##### *N*-(1-Hydroxy-1,3-dihydrobenzo[*c*][1,2]oxaborol-6-yl)-1,3-bis(trifluoromethyl)-1*H*-pyrazole-4-carboxamide (**4**)

The title compound was synthesized according to method B using a mixture of 1,3-bis(trifluoromethyl)-1*H*-pyrazole-4-carboxylic acid and 1,5-bis(trifluoromethyl)-1*H*-pyrazole-4-carboxylic acid (4 g, 16 mmol), HATU (7.3 g, 19.2 mmol), 6-aminobenzo[*c*][1,2]oxaborol-1(3*H*)-ol **2** (2.9 g, 19.2 mmol), DIPEA (4.1 g, 32 mmol), and DMF (30 mL) to yield 6.5 g of crude product as a yellow solid. 2 g of the crude product was purified by prep-HPLC to yield **4** as a white solid (200 mg). HPLC: 100% pure. MS (ESI) *m*/*z* = 380.1 [M + 1]^+^. ^1^H NMR (400 MHz, DMSO-*d*_6_) δ = 10.54 (s, 1H), 9.44 (s, 1H), 9.32 (brs, 1H), 8.18 (s, 1H), 7.65 (d, *J* = 7.2 Hz, 1H), 7.42 (d, *J* = 8.4 Hz, 1H), 4.98 (s, 2H). HRMS (ES+): calcd for C_13_H_9_B_1_F_6_N_3_O_3_ [M+H]^+^ 380.0641, found 380.0644 (0.75 ppm).

##### *N*-(1-Hydroxy-1,3-dihydrobenzo[*c*][1,2]oxaborol-6-yl)-1-(2,2,2-trifluoroethyl)-3-(trifluoromethyl)-1*H*-pyrazole-4-carboxamide (**5**)

To a cold solution of 6-amino-3,3-dimethylbenzo[*c*][1,2]oxaborol-1(3*H*)-ol **2** (22.7 g, 152.3 mmol) and DIPEA (39.3 g, 305.2 mmol) in THF (500 mL) was added a solution of 1-(2,2,2-trifluoroethyl)-3-(trifluoromethyl)-1*H*-pyrazole-4-carbonyl chloride (42.7 g, 152.5 mmol) in THF (150 mL) dropwise. The mixture was stirred at room temperature for 4 h and concentrated in a vacuum to a residue that was recrystallized from MeCN and water to yield **5** as a gray solid (41.1 g, 68%). HPLC: 98% pure. MS (ESI) *m*/*z* = 394.2 [M+H]^+^. ^1^H NMR (400 MHz, DMSO-*d*_6_) δ 10.37 (s, 1H), 9.27 (s, 1H), 8.72 (s, 1H), 8.16 (s, 1H), 7.66 (dd, *J* = 2.0, 8.0 Hz, 1H), 7.39 (d, *J* = 8.4 Hz, 1H), 5.48 (q, *J* = 9.2 Hz, 2H), 4.97 (s, 2H).

##### *N*-(1-Hydroxy-1,3-dihydrobenzo[*c*][1,2]oxaborol-6-yl)-3-(trifluoromethyl)-1-(3,3,3-trifluoropropyl)-1*H*-pyrazole-4-carboxamide (**6**)

The title compound was synthesized according to method B using 3-(trifluoromethyl)-1-(3,3,3-trifluoropropyl)pyrazole-4-carboxylic acid **6** (18.0 g, 65.2 mmol), HATU (34.7 g, 91.3 mmol), 1-hydroxy-3*H*-2,1-benzoxaborol-6-amine **2** (10.7 g, 71.8 mmol), DIPEA (16.9 g, 131.0 mmol), and DMF (150 mL) to give a residue. The residue was purified by silica gel chromatography (elution with ethyl acetate/petroleum ether/AcOH = 1: 3: 0.1) to give the crude product, which was recrystallized from MeOH to yield **6** as a white solid (9.60 g, 36.2%). HPLC: 99% pure. MS (ESI) *m*/*z* = 408.1 [M + 1]^+^. ^1^H NMR (400 MHz, DMSO-*d*_6_): δ 10.22 (s, 1H), 9.25 (s, 1H), 8.66 (s, 1H), 8.17 (s, 1H), 7.64 (d, *J* = 8.0 Hz, 1H), 7.38 (d, *J* = 8.0 Hz, 1H), 4.96 (s, 2H), 4.56 (t, *J* = 6.8 Hz, 2H), 3.02 (m, 2H). HRMS (ES+): calcd for C_15_H_13_B_1_F_6_N_3_O_3_ [M+H]^+^ 408.0954, found 408.0957 (0.70 ppm).

##### *N*-(1-Hydroxy-1,3-dihydrobenzo[*c*][1,2]oxaborol-6-yl)-3-methyl-1-(3,3,3-trifluoropropyl)-1*H*-pyrazole-4-carboxamide (**7**) and *N*-(1-Hydroxy-1,3-dihydrobenzo[*c*][1,2]oxaborol-6-yl)-5-methyl-1-(3,3,3-trifluoropropyl)-1*H*-pyrazole-4-carboxamide (**15**)

The title compounds were synthesized according to method B using a mixture of 3-methyl-1-(3,3,3-trifluoropropyl)-1*H*-pyrazole-4-carboxylic acid and 5-methyl-1-(3,3,3-trifluoropropyl)-1*H*-pyrazole-4-carboxylic acid (469 mg, 2.1 mmol), HATU (890 mg, 2.3 mmol), 6-aminobenzo[*c*][1,2]oxaborol-1(3*H*)-ol **2** (300 mg, 2.0 mmol), HATU (890 mg, 2.3 mmol), DIPEA (520 mg, 4.0 mmol), and DMF (5 mL) to yield the crude product, which was purified by preparative HPLC to give **7** (207 mg, 29%) as a white solid and **15** (205 mg, 29%) as a white solid.

**7**: HPLC: 99% pure. MS (ESI) *m*/*z* = 354[M+H]^+^. ^1^H NMR (400 MHz, DMSO-*d*_6_): δ = 9.77 (s, 1H), 9.21 (s, 1H), 8.42 (s, 1H), 8.14 (s, 1H), 7.68 (d, *J* = 8.0 Hz, 1H), 7.36 (d, *J* = 8.0 Hz, 1H), 4.97 (s, 2H), 4.38 (t, *J* = 6.8 Hz, 2H), 2.90 (m, 2H), 2.40 (s, 3H). HRMS (ES+): calcd for C_15_H_16_B_1_F_3_N_3_O_3_ [M+H]^+^ 354.1237, found 354.1240 (0.90 ppm).

**15**: HPLC: 99% pure. MS (ESI) *m*/*z* = 354[M+H]^+^. ^1^H NMR (400 MHz, DMSO-*d*_6_): δ = 9.79 (s, 1H), 9.22 (s, 1H), 8.16 (s, 1H), 8.12 (s, 1H), 7.79 (d, *J* = 8.4 Hz, 1H), 7.36 (d, *J* = 8.4 Hz, 1H), 4.96 (s, 2H), 4.35 (t, *J* = 6.8 Hz, 2H), 2.87 (m, 2H), 2.57 (s, 3H). HRMS (ES+): calcd for C_15_H_16_B_1_F_3_N_3_O_3_ [M+H]^+^ 354.1237, found 354.1240 (0.90 ppm).

##### Cyclobutyl-*N*-(1-hydroxy-1,3-dihydrobenzo[*c*][1,2]oxaborol-6-yl)-3-methyl-1*H*-pyrazole-4-carboxamide (**8**)

The title compound was synthesized according to method B using cyclobutyl-3-methyl-1*H*-pyrazole-4-carboxylic acid (201.0 mg, 1.1 mmol), 6-aminobenzo[*c*][1,2]oxaborol-1(3*H*)-ol **2** (167.0 mg, 1.1 mmol), HATU (543.0 mg, 1.4 mmol), DIPEA (180.0 mg, 1.4 mmol), and DMF (8 mL) to yield the crude product, which was purified by preparative HPLC to yield **8** as a white solid (96.6 mg, 28%). HPLC: 99% pure. MS (ESI) *m*/*z* = 312.1 [M +H]^+^. ^1^H NMR (400 MHz, DMSO-*d*_6_): δ 9.71 (s, 1H), 9.21 (s, 1H), 8.43 (s, 1H), 8.12 (d, *J* = 1.6 Hz, 1H), 7.67 (dd, *J* = 8.4, 2.0 Hz, 1H), 7.35 (d, *J* = 8.4 Hz, 1H), 4.96 (s, 2H), 4.80 (m, 1H), 2.40 (m, 7H), 1.82 (m, 2H). HRMS (ES+): calcd for C_16_H_19_B_1_N_3_O_3_ [M+H]^+^ 312.1519, found 312.1523 (1.13 ppm).

##### 1-Cyclobutyl-*N*-(1-hydroxy-1,3-dihydrobenzo[*c*][1,2]oxaborol-6-yl)-3-(trifluoromethyl)-1*H*-pyrazole-4-carboxamide (**9**)

The title compound was synthesized according to method B using 1-cyclobutyl-3-(trifluoromethyl)-1*H*-pyrazole-4-carboxylic acid (565 mg, 2.4 mmol), HATU (1.2 g, 3.2 mmol), 6-aminobenzo[*c*][1,2]oxaborol-1(3*H*)-ol **2** (430.0 mg, 2.9 mmol), DIPEA (805.0 mg, 6.2 mmol), and DMF (4 mL) to yield the crude product, which was purified by preparative HPLC to yield **9** as a white solid (278.0 mg, 32%). HPLC: 100% pure. MS (ESI) *m*/*z* = 366.0 [M + 1]^+^. ^1^H NMR (400 MHz, DMSO-*d*_6_) δ = 10.16 (s, 1H), 9.25 (s, 1H), 8.68 (s, 1H), 8.16 (s, 1H), 7.63 (dd, *J* = 1.6, 8.0 Hz, 1H), 7.38 (d, *J* = 8.4 Hz, 1H), 4.99 (m, 1H), 4.96 (s, 2H), 2.47 (m, 4H), 1.86 (m, 2H). HRMS (ES+): calcd for C_16_H_16_B_1_F_3_N_3_O_3_ [M+H]^+^ 366.1237, found 366.1240 (0.87 ppm).

##### *N*-(1-Hydroxy-1,3-dihydrobenzo[*c*][1,2]oxaborol-6-yl)-1-(tetrahydro-2*H*-pyran-4-yl)-3-(trifluoromethyl)-1*H*-pyrazole-4-carboxamide (**10**)

The title compound was synthesized according to method B using 1-(tetrahydro-2*H*-pyran-4-yl)-3-(trifluoromethyl)-1*H*-pyrazole-4-carboxylic acid (900 mg, 3.4 mmol), HATU (1.9 g, 5.1 mmol), 6-aminobenzo[*c*][1,2]oxaborol-1(3*H*)-ol **2** (507 mg, 3.4 mmol), and DIPEA (880 mg, 6.8 mmol) to yield the crude product, which was purified by preparative HPLC to yield **10** as a pink solid (358.6 mg, 26.7%). HPLC: 100% pure. MS (ESI) *m*/*z* = 396.0 [M + 1]^+^. ^1^H NMR (400 MHz, DMSO-*d*_6_) δ = 10.16 (s, 1H), 9.26 (s, 1H), 8.68 (s, 1H), 8.16 (s, 1H), 7.63 (d, *J* = 8.0 Hz, 1H), 7.38 (d, *J* = 8.0 Hz, 1H), 4.96 (s, 2H), 4.58 (m, 1H), 4.00 (m, 2H), 3.50 (t, *J* = 11.0 Hz, 2H), 2.07 (m, 2H), 1.96 (m, 2H). HRMS (ES+): calcd for C _17_ H_18_ B_1_Fe _3_N>_3_ O_4_ [M+H]^+^  396.1342, found 396.1346 (0.89 ppm).

##### *N*-(1-Hydroxy-1,3-dihydro-2,1-benzoxaborol-6-yl)-3-methyl-1-(pyridine-2-yl)-1*H*-pyrazole-4-carboxamide (**11**)

The title compound was synthesized according to method B using 3-methyl-1-(pyridin-2-yl)-1*H*-pyrazole-4-carboxylic acid (211 mg, 1.04 mmol), HATU (400 mg, 1.05 mmol), 6-aminobenzo[*c*][1,2]oxaborol-1(3*H*)-ol **2** (155 mg, 1.04 mmol), and DIPEA (0.27 mL, 1.56 mmol) to yield the crude product, which was triturated with DCM. The precipitated product was collected by filtration to give **11** (198 mg 54%) as an off-white solid. ^1^H NMR (DMSO-*d*_6_ with 0.05% v/v tetramethylsilane (TMS), 400 MHz): δ 2.52 (s, 3H, CH_3_), 4.97 (s, 2H, CH_2_), 7.37 (d, 1H, Ar, *J* = 8.0 Hz), 7.42 (ddd, 1H, ArH, *J* = 7.6 Hz, *J* = 5.2 Hz, *J* = 1.2 Hz), 7.73 (dd, ArH, *J* = 8.4 Hz, *J* = 2.0 Hz), 7.94 (d, 1H, ArH, *J* = 8.0 Hz), 8.03 (dd, 1H, ArH, *J* = 8.0 Hz, *J* = 1.6 Hz), 8.21 (d, 1H, ArH, *J* = 2.0 Hz), 8.54 (m, 1H, ArH), 9.47 (s, 1H, ArH), 9.70 (s, 1H, OH), 10.06 (s, 1H, NH).

##### 6-(((1-(Pyridin-2-yl)-3-(trifluoromethyl)-1*H*-pyrazole-4-carbonyl)oxy)amino)benzo[*c*][1,2]oxaborol-1(3*H*)-ol (**12**)

The title compound was synthesized according to method A using 1-(pyridin-2-yl)-3-(trifluoromethyl)-1*H*-pyrazole-4-carboxylic acid (200.0 mg, 0.78 mmol), 6-aminobenzo[*c*][1,2]oxaborol-1(3*H*)-ol **2** (120.0 mg, 0.8 mmol), HOBt (126.4 mg, 0.9 mmol), EDCI (172.8 mg, 0.9 mmol), DIPEA (201.0 mg, 1.6 mmol), and DMF (5 mL) to yield the crude product, which was purified by preparative HPLC to yield **12** as a white solid (200.0 mg, 66%). HPLC: 99% pure. MS (ESI) *m*/*z* = 389 [M+H]^+^. ^1^H NMR (400 MHz, DMSO-*d*_6_): δ 10.41 (s, 1H), 9.66 (s, 1H), 9.26 (s, 1H), 8.63 (m, 1H), 8.26 (d, *J* = 1.6 Hz, 1H), 8.15 (m, 1H), 8.04 (d, *J* = 8.4 Hz, 1H), 7.72 (dd, *J* = 8.0, 2.0 Hz, 1H), 7.58 (m, 1H), 7.40 (d, *J* = 8.4 Hz, 1H), 5.00 (s, 2H). HRMS (ES+): calcd for C _17_H_13_ B_1_F_3_ N_4_O_3_ [M+H]^+^  389.1033, found 389.1036 (0.82 ppm).

##### 6-(((1-(4-Chlorophenyl)-3-(trifluoromethyl)-1*H*-pyrazole-4-carbonyl)oxy)amino)benzo[*c*][1,2]oxaborol-1(3*H*)-ol (**13**)

The title compound was synthesized according to method B using 1-(4-chlorophenyl)-3-(trifluoromethyl)-1*H*-pyrazole-4-carboxylic acid (200 mg, 0.7 mmol), 6-aminobenzo[*c*][1,2]oxaborol-1(3*H*)-ol **2** (134 mg, 0.9 mmol), HATU (315.0 mg, 0.83 mmol), HATU (315.0 mg, 0.83 mmol), TEA (0.2 mL, 1.4 mmol), and DMF (5 mL) to yield the crude product, which was purified by preparative HPLC to yield **13** as a white solid (180.0 mg, 62%). HPLC: 99% pure. MS (ESI) *m*/*z* = 422.0 [M+H]^+^. ^1^H NMR (400 MHz, DMSO-*d*_6_): δ 10.32 (s, 1H), 9.32 (s, 1H), 9.26 (s, 1H), 8.19 (s, 1H), 7.92 (d, *J* = 8.8 Hz, 2H), 7.68 (m, 3H), 7.41 (d, *J* = 8.0 Hz, 1H), 4.98 (s, 2H).

##### *N*-(1-Hydroxy-1,3-dihydro-2,1-benzoxaborol-6-yl)-1-(2-methoxyphenyl)-3-methyl-1*H*-pyrazole-4-carboxamide (**14**)

The title compound was synthesized according to method B using 1-(2-methoxyphenyl)-3-methyl-1*H*-pyrazole-4-carboxylic acid (300 mg, 1.29 mmol), 6-aminobenzo[*c*][1,2]oxaborol-1(3*H*)-ol **2** (192 mg, 1.29 mmol), HATU (491 mg, 1.29 mmol), DIPEA (250 mg, 1.94 mmol), and DMF (3 mL) to yield the crude product, which was triturated with DCM. The precipitated product was collected by filtration and recrystallized from CHCl_3_ to give **14** (190 mg, 41%) as an off-white solid. ^1^H NMR (CDCl_3_ with 0.05% v/v TMS, 400 MHz): δ 2.50 (s, 3H, CH_3_), 3.93 (s, 3H, CH_3_), 4.97 (s, 2H, CH_2_), 7.09–7.14 (m, 1H, ArH), 7.29–7.32 (m, 1H, ArH), 7.36–7.43 (m, 2H, ArH), 7.67–7.72 (m, 2H, ArH), 8.14 (d, 1H, ArH, *J* = 1.6 Hz), 8.88 (s, 1H, ArH), 9.23 (s, 1H, OH), 9.89 (s, 1H, NH).

##### *N*-(1-Hydroxy-1,3-dihydrobenzo[*c*][1,2]oxaborol-6-yl)-5-methyl-1-(pyridin-2-yl)-1*H*-pyrazole-4-carboxamide (**16**)

A solution of 5-methyl-1-(pyridin-2-yl)-1*H*-pyrazole-4-carboxylic acid (15.0 g, 73.9 mmol) in SOCl_2_ (100 mL) was stirred at 50 °C for 4 h. The mixture was concentrated in a vacuum to give acyl chloride, which was used directly in the next step. To a solution of 6-aminobenzo[*c*][1,2]oxaborol-1(3*H*)-ol **2** (11.0 g, 73.8 mmol) and DIPEA (15 mL) in DCM (30 mL) was added a solution of acyl chloride in DCM (20 mL) dropwise at 0 °C. The mixture was stirred at room temperature for 1 h. The precipitate was filtered and the cake was washed with MTBE (100 mL) and water (50 mL), dried in a vacuum and recrystallized from THF (50 mL) to yield **16** as a white solid (16.0 g, 65%). HPLC: 100% pure. MS (ESI) *m*/*z* = 335.2 [M+H]^+^. ^1^H NMR (400 MHz, DMSO-*d*_6_) δ 9.99 (s, 1H), 9.24 (s, 1H), 8.57 (dd, *J* = 5.2, 1.2 Hz, 1H), 8.36 (s, 1H), 8.14 (d, *J* = 1.6 Hz, 1H), 8.07 (m, 1H), 7.84 (d, *J* = 8.0 Hz, 1H), 7.72 (m, 1H), 7.40 (m, 1H), 7.37 (d, *J* = 8.4 Hz, 1H), 4.98 (s, 2H), 2.85 (s, 3H).

##### *N*-(1-Hydroxy-1,3-dihydrobenzo[*c*][1,2]oxaborol-6-yl)-1-(6-methoxypyridin-2-yl)-5-methyl-1*H*-pyrazole-4-carboxamide (**17**)

The title compound was synthesized according to method B using 1-(6-methoxypyridin-2-yl)-5-methyl-1*H*-pyrazole-4-carboxylic acid (2.3 g, 10 mmol), 6-aminobenzo[*c*][1,2]oxaborol-1(3*H*)-ol **2** (1.50 g, 10 mmol), HATU (3.8 g, 10 mmol), DIPEA (3.9 g, 30 mmol), and DMF (20 mL) to yield the crude product, which was purified by preparative HPLC to yield **17** as a white solid (2.8 g, 77%). HPLC: 99% pure. MS (ESI) *m*/*z* = 365.0 [M+H]^+^. ^1^H NMR (400 MHz, DMSO-*d*_6_) δ 9.97 (s, 1H), 9.22 (s, 1H), 8.34 (s, 1H), 8.16 (d, *J* = 1.6 Hz, 1H), 7.94 (t, *J* = 8.0 Hz, 1H), 7.73 (dd, *J* = 8.0, 2.0 Hz, 1H), 7.45 (d, *J* = 7.6 Hz, 1H), 7.39 (d, *J* = 8.4 Hz, 1H), 6.88 (d, *J* = 8.0 Hz, 1H), 4.98 (s, 2H), 3.93 (s, 3H), 2.94 (s, 3H). HRMS (ES+): calcd for C_18_H_18_B_1_ N_4_O_4_ [M+H]^+^ 365.1421, found 365.1425 (1.07 ppm).

##### *N*-(1-Hydroxy-1,3-dihydrobenzo[*c*][1,2]oxaborol-6-yl)-5-methyl-1-(6-methylpyridin-2-yl)-1*H*-pyrazole-4-carboxamide (**18**)

The title compound was synthesized according to method B using 5-methyl-1-(6-methylpyridin-2-yl)-1*H*-pyrazole-4-carboxylic acid (100.0 mg, 0.5 mmol), 6-aminobenzo[*c*][1,2]oxaborol-1(3*H*)-ol **2** (82.0 mg, 0.5 mmol), HATU (210 mg, 0.6 mmol), DIPEA (89 mg, 0.7 mmol), and DMF (3 mL) to yield the crude product, which was purified by preparative HPLC to yield **18** as a white solid (34.8 mg, 22%). HPLC: 96% pure. MS (ESI) *m*/*z* = 349.1 [M + 1]^+^. ^1^H NMR (400 MHz, DMSO-*d*_6_): δ 9.95 (s, 1H), 9.21 (s, 1H), 8.33 (s, 1H), 8.15 (s, 1H), 7.92 (t, *J* = 7.6 Hz, 1H) 7.72 (d, *J* = 10.0 Hz, 1H), 7.61 (d, *J* = 8.0 Hz, 1H), 7.35 (m, 2H), 4.97 (s, 2H), 2.84 (s, 3H), 2.54 (s, 3H).

##### *N*-(1-Hydroxy-1,3-dihydrobenzo[*c*][1,2]oxaborol-6-yl)-3-(pyridin-2-yl)-1*H*-pyrazole-5-carboxamide (**19**)

To a solution of 6-aminobenzo[*c*][1,2]oxaborol-1(3*H*)-ol **2** (357.0 mg, 2.4 mmol) and DIPEA (619.0 mg, 4.8 mmol) in MeCN (10 mL) was added a solution of 3-(pyridin-2-yl)-1*H*-pyrazole-5-carbonyl chloride (500 mg, 2.4 mmol) in MeCN (10 mL) dropwise. The mixture was stirred at 60 °C for 20 h. The mixture was concentrated in a vacuum to give a crude product, which was purified by preparative HPLC to yield **19** as a white solid (28.4 mg, 3.7%). HPLC: 96% pure. MS (ESI) *m*/*z* = 321.1 [M + 1]^+^. ^1^H NMR (400 MHz, DMSO-*d*_6_) δ = 8.65 (d, *J* = 4.4 Hz, 1H), 8.21 (s, 1H), 8.01 (d, *J* = 8.0 Hz, 1H), 7.90 (t, *J* = 7.8 Hz, 1H), 7.82 (dd, *J* = 2.0, 8.0 Hz, 1H), 7.51 (s, 1H), 7.38 (m, 2H), 4.98 (s, 2H).

##### *N*-(1-Hydroxy-1,3-dihydrobenzo[*c*][1,2]oxaborol-6-yl)-1-methyl-3-(pyridin-2-yl)-1*H*-pyrazole-5-carboxamide (**20**) and *N*-(1-Hydroxy-1,3-dihydrobenzo[*c*][1,2]oxaborol-6-yl)-1-methyl-5-(pyridin-2-yl)-1*H*-pyrazole-3-carboxamide (**21**)

The title compounds were synthesized according to method B using a mixture of 1-methyl-3-(pyridin-2-yl)-1*H*-pyrazole-5-carboxylic acid and 1-methyl-5-(pyridin-2-yl)-1*H*-pyrazole-3-carboxylic acid (300 mg, 1.5 mmol), HATU (722 mg, 1.9 mmol), 6-aminobenzo[*c*][1,2]oxaborol-1(3*H*)-ol **2** (222.0 mg, 1.5 mmol), DIPEA (361.0 mg, 2.8 mmol), and DMF (3 mL) to yield the crude product, which was purified by preparative HPLC to give **20** (123 mg, 25%) as a white solid and **21** (40.7 mg, 8%) as a white solid.

**20:** HPLC: 99% pure. LC-MS (ESI) *m*/*z* = 335.1 [M + 1]^+^, *t* = 0.931 min. ^1^H NMR (400 MHz, DMSO-*d*_6_) δ = 10.42 (s, 1H), 9.29 (s, 1H), 8.63 (d, *J* = 4.4 Hz, 1H), 8.23 (s, 1H), 7.97 (d, *J* = 8.0 Hz, 1H), 7.87 (m, 1H), 7.78 (m, 2H), 7.41 (d, *J* = 8.0 Hz, 1H), 7.37 (m, 1H), 4.98 (s, 2H), 4.20 (s, 3H). HRMS (ES+): calcd for C_17_H_16_B_1_ N_4_O_3_ [M+H]^+^  335.1315, found 335.1319 (1.06 ppm).

**21:** HPLC: 99% pure. LC-MS (ESI) *m*/*z* = 335.1 [M + 1]^+^, *t* = 1.026 min. ^1^H NMR (400 MHz, DMSO-*d*_6_) δ = 10.18 (s, 1H), 9.26 (s, 1H), 8.73 (d, *J* = 4.8 Hz, 1H), 8.23 (s, 1H), 7.95 (m, 2H), 7.80 (d, *J* = 6.8 Hz, 1H), 7.45 (m, 1H), 7.36 (m, 2H), 4.97 (s, 2H), 4.28 (s, 3H). HRMS (ES+): calcd for C_17_H_16_B_1_N_4_O_3_ [M+H] ^+^ 335.1315, found 335.1319 (1.06 ppm).

##### *N*-(1-Hydroxy-1,3-dihydrobenzo[*c*][1,2]oxaborol-6-yl)-1-(pyridin-2-yl)-1*H*-1,2,3-triazole-4-carboxamide (**22**)

To a solution of 6-amino-3,3-dimethylbenzo[*c*][1,2]oxaborol-1(3*H*)-ol **2** (1.6 g, 10.5 mmol) and DIPEA (2.7 g, 21.0 mmol) in THF (20 mL) was added a solution of 1-(pyridin-2-yl)-1*H*-1,2,3-triazole-4-carbonyl chloride (2.2 g, 10.5 mmol) in THF (80 mL) at 0 °C. The mixture was stirred at room temperature for 4 h. The solvent was removed in a vacuum and the residue was recrystallized with MeCN and water to yield **22** as a gray solid (1.7 g, 51%). HPLC: 100% pure. MS (ESI) *m*/*z* = 321.9 [M+H]^+^. ^1^H NMR (400 MHz, DMSO-*d*_6_): δ 10.61 (s, 1H), 9.39 (s, 1H), 9.27 (s, 1H), 8.67 (d, *J* = 4.4 Hz, 1H), 8.20 (m, 3H), 7.82 (dd, *J* = 8.8, 2.0 Hz, 1H), 7.63 (m, 1H), 7.41 (d, *J* = 8.4 Hz, 1H), 4.98 (s, 2H). HRMS (ES+): calcd for C_15_H_12_B_1_N_5_O_3_Na_1_ [M+Na] ^+^ 344.0931, found 344.0934 (0.90 ppm).

##### *N*-(1-Hydroxy-1,3-dihydrobenzo[*c*][1,2]oxaborol-6-yl)-5-methyl-1-(pyridin-2-yl)-1*H*-1,2,3-triazole-4-carboxamide (**23**)

The title compound was synthesized according to method B using 5-methyl-1-(pyridin-2-yl)-1*H*-1,2,3-triazole-4-carboxylic acid (3.3 g, 16.2 mmol), 6-aminobenzo[*c*][1,2]oxaborol-1(3*H*)-ol **2** (2.9 g, 19.4 mmol), HATU (8.0 g, 21.0 mmol), DIPEA (4.2 g, 32.3 mmol), and DMF (30 mL) to yield the crude product, which was purified by preparative HPLC to yield **23** as a yellow solid (4.0 g, 74.1%). HPLC: 100% pure. MS (ESI) *m*/*z* = 336.1[M + 1]^+^. ^1^H NMR (400 MHz, DMSO-*d*_6_): δ 10.59 (s, 1H), 9.25 (s, 1H), 8.70 (dd, *J* = 4.8, 0.8 Hz, 1H), 8.28 (s, 1H), 8.19 (t, *J* = 8.0 Hz, 1H), 7.97 (d, *J* = 8.0 Hz, 1H), 7.83 (dd, *J* = 8.0, 2.0 Hz, 1H), 7.67 (d, *J* = 5.2 Hz, 1H), 7.38 (d, *J* = 8.0 Hz, 1H), 4.97 (s, 2H), 2.81 (s, 3H).

##### *N*-(1-Hydroxy-1,3-dihydrobenzo[*c*][1,2]oxaborol-6-yl)-5-methyl-1-phenyl-1*H*-1,2,3-triazole-4-carboxamide (**24**)

The title compound was synthesized according to method B using 5-methyl-1-phenyl-1*H*-1,2,3-triazole-4-carboxylic acid (870.0 mg, 4.3 mmol), 6-aminobenzo[*c*][1,2]oxaborol-1(3*H*)-ol **2** (870.0 mg, 5.8 mmol), HATU (1.7 g, 4.5 mmol), DIPEA (1.7 g, 13.2 mmol), and DMF (16 mL) to yield the crude product, which was purified by preparative HPLC to yield **24** as a white solid (0.61 g, 43%). HPLC: 98% pure. MS (ESI) *m*/*z* = 335 [M + 1]^+^. ^1^H NMR (400 MHz, DMSO-*d*_6_): δ 10.56 (s, 1H), 9.27 (s, 1H), 8.29 (s, 1H), 7.84 (dd, *J* = 8.0, 2.0 Hz, 1H), 7.69 (m, 5H), 7.40 (d, *J* = 8.4 Hz, 1H), 4.99 (s, 2H) 2.61 (s, 3H). HRMS (ES+): calcd for C_17_H_16_B_1_N_4_O_3_ [M+H] ^+^ 335.1315, found 335.1319 (1.06 ppm).

##### *N*-(1-Hydroxy-1,3-dihydrobenzo[*c*][1,2]oxaborol-6-yl)-1-(6-methoxypyridin-2-yl)-5-methyl-1*H*-1,2,3-triazole-4-carboxamide (**25**)

The title compound was synthesized according to method B using 1-(6-methoxypyridin-2-yl)-5-methyl-1H-1,2,3-triazole-4-carboxylic acid (150.0 mg, 0.6 mmol), 6-aminobenzo[*c*][1,2]oxaborol-1(3*H*)-ol **2** (114.0 mg, 0.77 mmol), HATU (365.0 mg, 1.0 mmol), DIPEA (165.0 mg, 1.3 mmol), and DMF (3 mL) to yield a crude product, which was purified by preparative HPLC to yield **25** as a white solid (134.5 mg, 58%). HPLC: 99% pure. MS (ESI) *m*/*z* = 366.1 [M+H]^+^. ^1^H NMR (400 MHz, DMSO-*d*_6_): δ 10.56 (s, 1H), 9.24 (s, 1H), 8.28 (m, 1H), 8.06 (t, *J* = 8.0 Hz, 1H), 7.83 (dd, *J* = 8.0, 2.0 Hz, 1H), 7.58 (d, *J* = 7.4 Hz, 1H), 7.39 (d, *J* = 8.4 Hz, 1H), 7.07 (d, *J* = 8.4 Hz, 1H), 4.98 (s, 2H), 3.95 (s, 3H), 2.91 (s, 3H). HRMS (ES+): calcd for C_17_H_17_B_1_N_5_O_4_ [M+H]^+^  366.1374, found 366.1377 (0.93 ppm).

##### *N*-(1-Hydroxy-1,3-dihydrobenzo[*c*][1,2]oxaborol-6-yl)-5-methyl-1-(6-methylpyridin-2-yl)-1*H*-1,2,3-triazole-4-carboxamide (**26**)

The title compound was synthesized according to method B using 5-methyl-1-(6-methylpyridin-2-yl)-1*H*-1,2,3-triazole-4-carboxylic acid (80.0 mg, 0.4 mmol), 6-aminobenzo[*c*][1,2]oxaborol-1(3*H*)-ol **2** (89.0 mg, 0.6 mmol), HATU (228.0 mg, 0.6 mmol), DIPEA (103.0 mg, 0.8 mmol), and DMF (3 mL) to yield the crude product, which was purified by preparative HPLC to yield **26** as a yellow solid (45 mg, 35%). HPLC: 95% pure. MS (ESI) *m*/*z* = 350.2 [M+H]^+^. ^1^H NMR (400 MHz, DMSO-*d*_6_): δ 10.56 (s, 1H), 9.24 (s, 1H), 8.28 (s, 1H), 8.07 (t, *J* = 8.0 Hz, 1H), 7.83 (d, *J* = 8.0 Hz, 1H), 7.75 (d, *J* = 8.0 Hz, 1H), 7.53 (d, *J* = 7.6 Hz, 1H), 7.39 (d, *J* = 8.0 Hz, 1H), 4.98 (s, 2H), 2.82 (s, 3H), 2.59 (s, 3H). HRMS (ES+): calcd for C_17_H_17_B_1_N_5_O_3_ [M+H] ^+^ 350.1424, found 350.1428 (1.01 ppm).

##### *N*-(1-Hydroxy-1,3-dihydrobenzo[*c*][1,2]oxaborol-6-yl)-5-methyl-1-(pyridin-3-yl)-1*H*-1,2,3-triazole-4-carboxamide (**27**)

The title compound was synthesized according to method B using 5-methyl-1-(pyridin-3-yl)-1*H*-1,2,3-triazole-4-carboxylic acid (150.0 mg, 0.7 mmol), 6-aminobenzo[*c*][1,2]oxaborol-1(3*H*)-ol **2** (109.0 mg, 0.7 mmol), HATU (380 mg, 1.0 mmol), DIPEA (188.0 mg, 1.5 mmol), and DMF (5 mL) to yield the crude product, which was purified by preparative HPLC to yield **27** as a white solid (154.6 mg, 63%). HPLC: 100% pure. MS (ESI) *m*/*z* = 336.2 [M+ 1]^+^. ^1^H NMR (400 MHz, DMSO-*d*_6_) δ 10.60 (s, 1H), 9.23 (s, 1H), 8.93 (d, *J* = 2.4 Hz, 1H), 8.85 (d, *J* = 4.8 Hz, 1H), 8.28 (d, *J* = 1.6 Hz, 1H), 8.20 (d, *J* = 7.6 Hz, 1H), 7.83 (d, *J* = 10.0 Hz, 1H), 7.74 (m, 1H), 7.39 (d, *J* = 8.0 Hz, 1H), 4.98 (s, 2H), 2.63 (s, 3H). HRMS (ES+): calcd for C_16_H_15_B_1_N_5_O_3_ [M+H]^+^ 336.1268, found 336.1271 (0.89 ppm).

##### *N*-(1-Hydroxy-1,3-dihydrobenzo[*c*][1,2]oxaborol-6-yl)-3-methyl-4-(pyridin-2-yl)furan-2-carboxamide (**28**)

To a solution of 3-methyl-4-(pyridin-2-yl)furan-2-carboxylic acid (1.7 g, 8.3 mmol) in DCM (20 mL) was added (COCl)_2_ (2.4 mL, 25.1 mmol) and DMF (50 μL). The mixture was stirred at room temperature for 8 h. The mixture was concentrated in a vacuum to give acyl chloride. To a solution of 6-aminobenzo[*c*][1,2]oxaborol-1(3*H*)-ol **2** (1.2 g, 8.1 mmol) and DIPEA (2.9 mL, 16.7 mmol) in THF (30 mL) was added acyl chloride. The mixture was stirred at room temperature for 16 h. Water (150 mL) was added and the mixture was extracted with EtOAc (150 mL × 4). The combined organic extracts were washed with brine (100 mL × 2), dried with anhydrous Na_2_SO_4_, filtered, and concentrated in a vacuum to give a residue. The residue was purified by silica gel chromatography (elution with DCM/EtOH = 50: 1) to give a crude product, which was recrystallized from MeCN (100 mL) to yield **28** as a white solid (1.6 g, 57%). HPLC: 98% pure. MS (ESI) *m*/*z* = 335.0 [M + 1]^+^. ^1^H NMR (400 MHz, DMSO-*d*_6_) δ 10.20 (s, 1H), 9.24 (s, 1H), 8.65 (d, *J* = 4.0 Hz, 1H), 8.37 (s, 1H), 8.18 (s, 1H), 7.87 (m, 1H), 7.75 (m, 1H), 7.30 (m, 3H), 4.96 (s, 2H), 2.62 (s, 3H).

##### *N*-(1-Hydroxy-1,3-dihydrobenzo[*c*][1,2]oxaborol-6-yl)-5-methyl-4-(pyridin-2-yl)furan-2-carboxamide (**29**)

To a solution of 5-methyl-4-(pyridin-2-yl)furan-2-carboxylic acid (5.7 g, 28.1 mmol), DIPEA (7.5 g, 58.1 mmol), and HATU (16.0 g, 42.1 mmol) in DMF (60 mL) was added 6-aminobenzo[*c*][1,2]oxaborol-1(3*H*)-ol **2** (4.2 g, 28.2 mmol). The mixture was stirred at room temperature overnight. Water (200 mL) was added and a lot of solid formed. The suspension was filtered and the cake was collected and purified by preparative HPLC to yield **29** as a white solid (6.5 g, 65.6%). HPLC: 100% pure. MS (ESI) *m*/*z* = 334.9 [M + 1]^+^. ^1^H NMR (400 MHz, DMSO-*d*_6_) δ 10.20 (s, 1H), 9.27 (s, 1H), 8.65 (d, *J* = 4.0 Hz, 1H), 8.17 (s, 1H), 7.88 (m, 2H), 7.75 (m, 2H), 7.40 (d, *J* = 8.4 Hz, 1H), 7.30 (m, 1H), 4.98 (s, 2H), 2.76 (s, 3H). HRMS (ES+): calcd for C_18_H_16_B_1_N_2_O_4_ [M+H]^+^ 335.1203, found 335.1206 (0.86 ppm).

##### *N*-(1-Hydroxy-1,3-dihydrobenzo[*c*][1,2]oxaborol-6-yl)-3-methyl-4-(pyridin-2-yl)thiophene-2-carboxamide (**30**)

The title compound was synthesized according to method B using 3-methyl-4-(pyridin-2-yl)thiophene-2-carboxylic acid (100 mg, 0.46 mmol), 6-aminobenzo[*c*][1,2]oxaborol-1(3*H*)-ol **2** (80 mg, 0.54 mmol), HATU (200 mg, 0.53 mmol), DIPEA (300 mg, 2.33 mmol), and DMF (2 mL) to yield the crude product, which was purified by preparative HPLC to yield **30** as a white solid (63.8 mg). HPLC: 99% pure. MS (ESI) *m*/*z* = 351.1 [M + 1]^+^. ^1^H NMR (400 MHz, DMSO-*d*_6_) δ 10.26 (s, 1H), 9.27 (s, 1H), 8.70 (d, *J* = 4.0 Hz, 1H), 8.11 (s, 1H), 8.03 (s, 1H), 7.74 (t, *J* = 2.0 Hz, 1H), 7.70 (m, 2H), 7.40 (m, 2H), 4.98 (s, 2H), 2.55 (s, 3H). HRMS (ES+): calcd for C_18_H_16_B_1_N_2_O_3_S_1_ [M+H]^+^  351.0975, found 351.0978 (0.94 ppm).

##### *N*-(1-Hydroxy-1,3-dihydrobenzo[*c*][1,2]oxaborol-6-yl)-5-(pyridin-2-yl)-1,3,4-oxadiazole-2-carboxamide (**31**)

To a reaction mixture of 5-(pyridin-2-yl)-1,3,4-oxadiazole-2-carbonyl chloride were added DIPEA (6.8 g, 52.7 mmol) and a solution of 1-hydroxy-3*H*-2,1-benzoxaborol-6-amine **2** (2.5 g, 17 mmol) in MeCN (50 mL) at 0 °C. The mixture was stirred at 0 °C for 1 h. The reaction mixture was quenched by adding water (100 mL) at room temperature and extracted with EtOAc (100 mL × 2). The combined organic extracts were washed with brine (200 mL × 2), dried over Na_2_SO_4_, filtered, and concentrated under reduced pressure to give a residue. The residue was dispersed in DCM (20 mL), filtered, and the cake was dried under reduced pressure to give a crude product, which was purified by preparative HPLC to yield **31** as a white solid (1.9 g, 45%). HPLC: 99% pure. MS (ESI) *m*/*z* = 323.2 [M+H]^+^. ^1^H NMR (400 MHz, DMSO-*d*_6_): δ 11.36 (s, 1H), 9.32 (s, 1H), 8.84 (d, *J* = 2.4 Hz, 1H), 8.32 (m, 1H), 8.25 (s, 1H), 8.12 (m, 1H), 7.85 (m, 1H), 7.72 (d, *J* = 3.6 Hz, 1H), 7.46 (d, *J* = 8.4 Hz, 1H), 5.00 (s, 2H).

### Biology

#### Compounds and Reagents

For *in vitro* assays, compound stock solutions were prepared in 100% dimethyl sulfoxide (DMSO) at 20 mM. Compounds were serially prediluted (2-fold or 4-fold) in DMSO, followed by a further (intermediate) dilution in demineralized water to assure a final in-test DMSO concentration of <1%. For *in vivo* studies, compounds were formulated in 2% ethanol, 1 mol equiv NaOH, and dextrose (5% solution) when administrated orally to mice, hamsters, and dogs or intravenously (pH adjusted to 7) to rats and dogs. For oral administration in rats, 0.5% (w/v) methylcellulose and 0.1% (w/v) sodium dodecyl sulfate in Milli-Q water were used. Miltefosine was formulated in water at 20 mg/mL.

#### Cell Cultures

Primary peritoneal mouse macrophages (PMM) were collected two days after peritoneal stimulation with a 2% potato starch suspension. MRC5_SV2_ cells (diploid human embryonic lung fibroblasts) were cultured in minimal essential medium (MEM) containing Earle’s salts, supplemented with l-glutamine, NaHCO_3_, and 5% inactivated fetal calf serum. All cultures and assays were conducted at 37 °C under an atmosphere of 5% CO_2_.

#### Parasites

*L. infantum* (MHOM/MA/67/ITMAP263) and *L. donovani* (MHOM/ET/67/L82) were maintained in the golden (Syrian) hamster (*Mesocricetus auratus*). *Ex vivo* amastigotes were collected from the spleen of an infected donor hamster using two centrifugation purification steps: 230*g* for 10 min, keeping the supernatant layer, and 4100*g* for 30 min, keeping the pellet. The spleen parasite burden was assessed using the Stauber technique.^25^ For *in vitro* assays, the inoculum was prepared in RPMI-1640 medium, supplemented with 200 mM l-glutamine, 16.5 mM NaHCO_3_, and 5% inactivated fetal calf serum. For the *in vivo* model, an infection inoculum containing 2 × 10^7^ amastigotes/100 μL was prepared in phosphate-buffered saline (PBS).

#### Animals

Female golden hamsters for the *in vivo* model of visceral leishmaniasis were purchased from Janvier, France (body weight 80–100 g). This study using laboratory rodents was carried out in strict accordance with all mandatory guidelines (EU directives, including the Revised Directive 2010/63/EU on the protection of Animals used for Scientific Purposes that came into force on 01/01/2013, and the declaration of Helsinki in its latest version) and was approved by the ethical committee of the University of Antwerp, Belgium (UA-ECD 2011-74). Female golden hamsters for the pharmacokinetic study were purchased from Vital River, Beijing, China. This study was conducted following institutional review and in accordance with institutional and national guidelines at WuXi AppTec (the Institutional Animal Care and Use Committee (IACUC)). Balb/c mice for the *in vivo* model of visceral leishmaniasis (LSHTM) were purchased from Charles River, U.K., and related studies were conducted under license PPL 70/8427 from the U.K. Home Office.

#### Intramacrophage *L. infantum* and *L. donovani* Assays

The assay was performed in sterile 96-well microtiter plates, each well containing 10 μL of the compound dilution and 190 μL of the PMM/amastigote inoculum (3 × 10^4^ cells/4.5 × 10^5^ parasites per well). Parasite multiplication was compared to untreated infected controls (100% growth) and uninfected controls (0% growth). After five-day incubation, total parasite burdens were microscopically assessed after staining the cells with a 10% Giemsa solution. The results were expressed as a percentage reduction in parasite burden compared to untreated control wells. EC_50_ values were determined using an extended dose range (2-fold compound dilutions, 8-point concentration curve). Miltefosine was used as the reference drug.^26^ For selected compounds, this assay format was also run using the *L. donovani* inoculum.

#### *In Vitro* MRC5 and PMM Cytotoxicity Assays

Assays were performed in sterile 96-well microtiter plates, each well containing 10 μL of the compound dilution and 190 μL of MRC5_SV2_ or PMM inoculum (3 × 10^4^ cells/mL). Cell growth was compared to untreated controls (100% growth) and assay-media controls (0% growth). After three-day incubation, cell viability was assessed fluorometrically by adding resazurin (50 μL/well of a stock solution in phosphate buffer (50 μg/mL)), incubating for 4 h and measuring fluorescence (λ_ex_ 550 nm, λ_em_ 590 nm). The results were expressed as a percentage reduction in cell growth compared to untreated control wells. EC_50_ values were determined using an extended dose range (2-fold compound dilutions, 8-point concentration curve) to a highest concentration of 64 μM. Tamoxifen was included as the reference drug.

#### *In Vivo* Hamster Model of Visceral Leishmaniasis

Female golden hamsters were randomly allocated to experimental groups of six animals each, based on body weight. At the start of the experiment (Day 0), each animal was infected with *L. infantum* or *L. donovani* inoculum, delivered by intracardial injection. Six hamsters were assigned to the vehicle-treated, infected control group. Six hamsters were assigned per group (1 group/compound) for the evaluation of compound and miltefosine. At Day 21 postinfection (21 dpi), all animals in each group were dosed orally for five (or ten) consecutive days (21–25 dpi): compound was dosed at 25–50 mg/kg b.i.d.; miltefosine was dosed at 40 mg/kg q.d. At Day 35 (10 days after the final oral dose), all animals were euthanized and autopsies were conducted. The study evaluated the following parameters.

1. Adverse effects: all animals were observed daily for the occurrence/presence of adverse effects.
2. Body weight: all animals were weighed twice per week to monitor general health.
3. Parasite burden: amastigote burdens in each target organ (liver, spleen, and bone marrow) were determined at Day 35. The organs of individual animals were weighed (except for bone marrow). Impression smears were stained with Giemsa for microscopic examination of the total amastigote burden, defined as the mean number of amastigotes per cell multiplied by the number of cells counted (minimum 500 nuclei); the results were expressed as a percentage reduction in amastigote burden compared to vehicle-treated, infected control animals.

#### *In Vivo* Mouse Model of Visceral Leishmaniasis

Female BALB/c mice were infected with 2 × 10^7^*L. donovani* amastigotes/0.2 mL i.v. harvested from the spleen of an infected donor RAG1B6 mouse (LSHTM). After 7 days, the mice were treated with miltefosine or DNDI-6148 for 5 consecutive days. Five days after the final dose, mice were humanely killed, liver and spleens were dissected and weighed, and impression smears were made for the calculation of parasite burden (Stauber equation).^25^

#### Liver Microsome Stability

Tests were performed by WuXi AppTec, China. Test compounds (at 1 μM) or positive controls (testosterone, propafenone, and diclofenac) were incubated at 37 °C with liver microsomes from Balb/c mouse, golden (Syrian) hamster, Sprague-Dawley rat, Beagle dog, or human in the presence of a NADPH regenerating system and phosphate buffer (100 mM, pH 7.4) at 0.4 or 0.8 mg/mL microsomal protein. The samples were removed at time intervals from 0 to 60, 90, or 120 min and immediately mixed with cold methanol supplemented or not with acid (3% formic acid) and centrifuged prior to analysis by LC-MS/MS using tolbutamide or propranolol as internal standards.

#### Hepatocyte Stability

Tests were performed by WuXi AppTec, China. Following a viability check of cryopreserved hepatocytes from Sprague-Dawley rat, Beagle dog, or human, test compounds (at 1 μM) or positive controls 7-ethoxycoumarin and 7-hydroxycoumarin (at 3 μM) were incubated at 37 °C, 5% CO_2_ with hepatocytes in Williams’ Medium E. Following incubation of 15, 30, 60, 90, 120, 180, and 240 min, reactions were stopped by the addition of acetonitrile, the samples were centrifuged, and LC-MS/MS analysis was conducted using tolbutamide as an internal standard.

#### Metabolite ID Studies

Metabolite identification studies were conducted at WuXi AppTec, China. DNDI-6148 was incubated at 10 μM in the presence of Beagle dog and Sprague-Dawley rat liver microsomes (1 mg/mL) for 60 min. Following precipitation with a solution of formic acid and acetonitrile, metabolites were identified by LC-UV-MSn (*n* = 1–2).

#### CYP Inhibition Studies

The study was conducted by WuXi AppTec, Co., China, using the time-dependent (TDI) method. For detecting any IC_50_ shift, the test compound (at concentrations of 0.05–50 μM) was first preincubated for 30 min at 37 °C with pooled human liver microsomes in the presence or not of NADPH. Following incubation with substrates (phenacetin for 1A2, diclofenac for 2C9, *S*-(+) mephenytoin for 2C19, dextromethorphan for 2D6, and midazolam or testosterone for 3A4), reactions were stopped by adding ice-cold acetonitrile, the samples were analyzed for the formation of metabolites by LC-MS/MS, and the percentage of inhibition was determined.

#### hERG Inhibition

An automated patch-clamp method (QPatch HTX) was used by WuXi AppTec, China. Chinese hamster ovary cells expressing hERG potassium channels were incubated at room temperature with test compounds (0.12, 0.37, 1.11, 3.33, 10, and 30 μM, in triplicate) or amitriptyline as a positive control.

#### Plasma-Protein Binding

Tests were conducted by WuXi AppTec, Co., China, using the equilibrium dialysis method and a 96-well plate format. The test compound (at 2 μM, in triplicate) in solution in plasma (Balb/c mouse, golden (Syrian) hamster, Sprague-Dawley rat, Beagle dog or human) was dialyzed against 100 mM phosphate-buffered saline (pH 7.4) on a rotating plate incubated for 6 h at 37 °C. Following the precipitation of protein with acetonitrile or acetonitrile/methanol containing both 1% phosphoric acid, the amount of compound present in each compartment was quantified by LC-MS/MS.

#### Thermodynamic Solubility

The thermodynamic solubility was measured by WuXi AppTec, China. Aliquots of the compound DMSO stock (10 mM) were transferred to fasted state simulated intestinal fluid (FaSSIF) buffer (pH 6.5) or fed state simulated intestinal fluid (FeSSIF) buffer (pH 5.0), and the mixtures were shaken for 24 h at room temperature. Following sampling by a Whatman filter device, the compound concentrations were determined by UV spectroscopy.

#### Membrane Permeability

Bidirectional permeability tests with MDCK-MDR1 cells were performed by WuXi AppTec, Co., China. The test compound, at a concentration of 2 μM in 0.4% DMSO/HBSS buffer with 10 mM HEPES pH 7.4, was applied to the apical or basolateral side of the cell monolayer. Permeation of the compound from A to B direction or B to A direction was determined in triplicate over a 150 min incubation at 37 °C and 5% CO_2_ (95% humidity). Test and reference compounds were quantified by LC-MS/MS analysis based on the peak area ratio of analyte/internal standard.

#### Ames Assay

Mini-Ames reverse mutation screens were conducted by WuXi AppTec, Co., China. Two *Salmonella typhimurium* strains (TA98 and TA100) were employed, both in the presence and absence of metabolic activation (induced rat liver S9). Test compounds were assessed at doses of 1.5, 4, 10, 25, 64, 160, 400, and 1000 μg per well, 2-aminoanthracene, 2-nitrofluorene, and sodium azide were used as positive controls, and the negative control was DMSO.

#### *In Vivo* Pharmacokinetic Studies

*In vivo* pharmacokinetic studies were conducted at WuXi AppTec, Co., China.

#### Hamster

Compounds were formulated as a homogenous suspension in 2% ethanol, 1 mol equiv NaOH, dextrose (5% solution), and dosed twice p.o. on a single day (8 h apart, 25 or 50 mg/kg, BID) in female golden Syrian hamsters. The K_2_-EDTA anticoagulant was added to blood samples collected from 0.25 to 36 h, which were processed to plasma and stabilized with phosphoric acid (1% final) before analysis by LC-MS/MS. PK parameters were calculated using WinNonlin software (version 6.3).

#### Rat

DNDI-6148 was formulated in 2% ethanol, 1 mol equiv NaOH, dextrose (5% solution), and administrated to male Sprague-Dawley rats, either i.v. after filtration and pH adjustment (at 2 mg/kg), or p.o. as a homogenous suspension (at 10 mg/kg). The K_2_-EDTA anticoagulant was added to blood samples collected at predose, 0.083 (i.v. only), 0.25, 0.5, 1, 2 (p.o. only), 4, 8, and 24 h, and were processed to plasma and stabilized with phosphoric acid (1% final) before analysis by LC-MS/MS. PK parameters were calculated using WinNonlin software (version 6.21 and 6.3).

#### Dog

DNDI-6148 was administrated intravenously at 1 mg/kg and orally at 5 mg/kg to male Beagle dogs. The formulation for the i.v. leg was 2% ethanol, 1.0 mol equiv NaOH, dextrose (5% solution) filtered, and adjusted to pH 7, and for the p.o. leg, the formulation was 2% ethanol, 1.0 mol equiv NaOH, and dextrose (5% solution). The K_2_-EDTA anticoagulant was added to blood samples collected at predose, 0.033 (i.v. only), 0.083 (p.o. only), 0.25, 0.5, 1, 3, 6, 9, and 24 h, and were stabilized with phosphoric acid (1% final) before analysis by LC-MS/MS. PK parameters were calculated using WinNonlin software (version 6.3).

### MoA Studies

#### Synthesis of DNDI-6148, *N*-(1-Hydroxy-1,3-dihydrobenzo[*c*][1,2]oxaborol-6-yl)-5-methyl-1-(pyridin-2-yl)-1*H*-1,2,3-triazole-4-carboxamide (**23**) Used in MoA studies

All NMR spectra for the compounds and intermediates made in the course of these studies are available upon request.

**Scheme 5:**
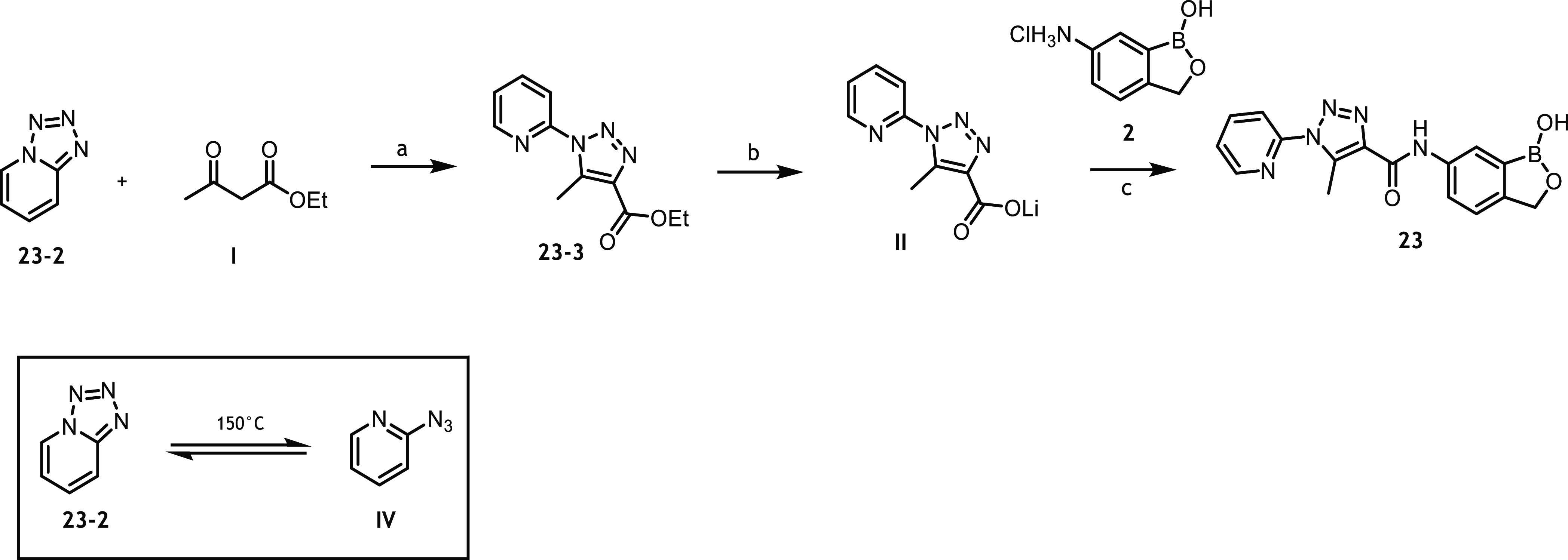
Synthetic Route to DNDI-6148 (**23**) Employed for the Material Used in the MoA Studies Reagents and conditions: (a) NaOEt, EtOH, 150 °C, 2 h (flow), 38%; (b) LiOH, THF/water, room temp., 16 h, 97%; and (c) **2**, T3P, DIPEA, DMF/EtOAc, RT, 16 h, 29%.

The samples of DNDI-6148 used in the MoA studies were prepared using a three-step sequence (Scheme 5) modified from a synthetic route previously reported by Jacobs and co-workers.^27^ In brief, triazole **23-3** was prepared using an optimized flow chemistry procedure to alleviate the potential risk of explosion when heating low molecular weight organic azides. Note, tetrazole **23-2** is converted to the azide reactant (**IV**) under the thermal reaction conditions.^28^ Treatment with LiOH hydrolyzed the ester function of **23-3** to give lithium salt **II**, which was converted to DNDI-6148 (**23**) by reaction with aniline **2**, T3P, and base.

#### Ethyl 5-Methyl-1-(pyridin-2-yl)-1*H*-1,2,3-triazole-4-carboxylate (**23-3**)

A solution of tetrazolo[1,5-*a*]pyridine **(23-2)** (144 mg, 1.2 mmol) and ethyl 3-oxobutanoate (**I**) (1.56 g, 5.99 mmol) in EtOH (10 mL) and a solution of NaOEt (245 mg, 3.6 mmol) in EtOH (10 mL) were mixed in a 1:1 ratio and applied to the flow coil (Vapourtec RS-400 flow chemistry system). The reaction mixture was then heated at 150 °C with a residence time of 2 h. The crude product mixture was collected into a vial and acidified to pH 5 by the addition of HCl (2N aq.). The reaction mixture was then concentrated under reduced pressure and directly purified by silica flash column chromatography (10:90 → 50:50 EtOAc:heptane), affording ester **23-3** as a colorless solid (110 mg, 38% yield). ^1^H NMR (500 MHz, CDCl_3_) δ 8.59 (1H, d, *J* = 4.75 Hz), 8.00–7.93 (2H, m), 7.42 (1H, t, *J* = 6.03 Hz), 4.47 (2H, q, *J* = 7.12 Hz), 2.92 (3H, s), 1.45 (3H, t, *J* = 7.10 Hz). ^13^C NMR (126 MHz, CDCl_3_) δ 161.7, 150.2, 148.5, 139.7, 139.1, 137.4, 124.1, 118.3, 61.0, 14.3, 11.0. HRMS (ES^+^): *m*/*z* [M+H]^+^ calcd for C_11_H_13_N_4_O_2_, 233.1033; found, 233.1061.

#### Lithium 5-Methyl-1-(pyridin-2-yl)-1*H*-1,2,3-triazole-4-carboxylate (**II**)

A solution of LiOH (1 M, aq, 0.31 mL, 0.31 mmol) was added to a solution of ethyl 5-methyl-1-(pyridin-2-yl)-1*H*-1,2,3-triazole-4-carboxylate (**23-3**) (72 mg, 0.31 mmol) in THF/water (4:1, 10 mL), and the resulting mixture was stirred at room temperature for 16 h. The reaction mixture was then concentrated under reduced pressure, and the resultant aqueous residue was lyophilized to furnish the title compound as a colorless solid (64 mg, 97%). ^1^H NMR (500 MHz, D_2_O) δ 8.74 (1H, d, *J* = 4.05 Hz), 8.28 (1H, t, *J* = 7.80 Hz), 7.84 (1H, d, *J* = 8.00 Hz), 7.79 (1H, dd, *J* = 7.15, 5.35 Hz), 2.67 (3H, s). ^13^C NMR (126 MHz, D_2_O) δ 168.4, 149.0, 147.9, 141.9, 140.7, 137.6, 125.9, 120.6, 9.3. HRMS (ES^+^): *m*/*z* [M+H]^+^ calcd for C_9_H_9_N_4_O_2_, 205.0720; found, 205.0686.

#### DNDI-6148, *N*-(1-Hydroxy-1,3-dihydrobenzo[*c*][1,2]oxaborol-6-yl)-5-methyl-1-(pyridin-2-yl)-1*H*-1,2,3-triazole-4-carboxamide (**23**)

Neat DIPEA (130 mg, 1.28 mmol) was added to a stirred suspension of carboxylate **II** (45 mg, 0.214 mmol) and 6-aminobenzo[*c*][1,2]oxaborol-1(3*H*)-ol, hydrochloride (**5**) (59.6 mg, 0.321 mmol) in dry DMF (3.0 mL) at room temperature. A solution of propylphosphonic anhydride (50% w/v in EtOAc, 0.757 mL, 0.642 mmol) was then added dropwise, and the resulting mixture was stirred for a further 16 h at room temperature. The reaction mixture was then concentrated under reduced pressure and directly purified by silica flash column chromatography (2:98 MeOH:CH_2_Cl_2_), affording the title compound as a colorless solid (21.7 mg, 95% purity, 29% yield). The product was further purified by reverse phase preparative HPLC (C18 silica, 5:95 → 95:5 MeCN:water + 0.1% NH_3_) to give an assay/analytical sample at >95% purity. ^1^H NMR (400 MHz, DMSO) δ 10.53 (1H, s), 9.20 (1H, s), 8.71 (1H, ddd, *J* = 4.86, 1.78, 0.72 Hz), 8.27 (1H, d, *J* = 1.76 Hz), 8.19 (1H, dt, *J* = 11.79, 1.87 Hz), 7.98 (1H, d, *J* = 8.08 Hz), 7.84 (1H, dd, *J* = 8.22, 2.02 Hz), 7.68 (1H, ddd*, J* = 7.51, 4.87, 0.95 Hz), 7.39 (1H, d, *J* = 8.32 Hz), 4.98 (2H, s), 2.82 (3H, s). ^13^C NMR (126 MHz, DMSO) δ 159.8, 149.8, 149.7, 149.5, 140.5, 139.4, 138.1, 137.7, 125.5, 124.3, 123.0, 121.9, 119.5, 70.2, 10.7 (note, the signal for the benzene quaternary carbon atom directly linked to the boron atom was not observed in the ^13^C NMR due to severe broadening from scalar relaxation caused by the spin dynamics of the quadrupolar ^11^B nucleus). HRMS (ES^+^): *m*/*z* [M+H]^+^ calcd for C_16_H_15_BN_5_O_3_, 336.1262; found, 336.1271.

### Cell Lines and Culture Conditions

The clonal *L. donovani* cell line LdBOB (derived from MHOM/SD/62/1S-CL2D) was grown as promastigotes at 28 °C, as described previously. Bloodstream form *T. brucei* MiTat 1.2 clone 221a and its derived subline, 2T1, were cultured at 37 °C in the presence of 5% CO_2_ in HMI9-T medium, as described previously. *T. brucei* cell lines overexpressing CPSF3 and overexpressing a mutated version of CPSF3 (Asn232His) were generated as part of a previous study and cultured as described.^19^

### Generation of *L. donovani* Transgenic Cell Lines

*L. donovaniCPSF*3 gene (LdBPK_343210.1) was custom synthesized by GeneArt and ligated into the pIR1SAT vector via BglII restriction sites. Using the Q5 mutagenesis kit (NEB) and the pIR1SAT-*LdCPSF*3^WT^ construct as a template, mutations encoding A655C and Asn219His change to CPSF3^WT^ were introduced. Mutations were introduced using the specific primers 5′-GAGTACACGGCATCCGC-3′ and 5′-TCCGCAATCAGGATGTCTGGT-3′ and as per manufacturers’ instructions. Wild-type and mutated pIR1SAT constructs were digested with SwaI and transfected into *L. donovani* promastigotes, as previously described. Resistant clones were selected with nourseothricin (100 μg/mL), and two clones per transfection were selected for further study.

### CRISPR-Cas9 Generated Mutations in *Ld*CPSF3

A cell line constitutively expressing Cas9 and T7 RNA polymerase was generated by transfecting 4 × 10^7^ promastigotes with the pTB007 vector (10 μg), as previously described.^29^ Expression of Cas9 in selected clones was confirmed by western blotting (Figure S1). In addition, the CRISPR-Cas9 system in these parasites was confirmed as functional following the successful tagging of the flagellar protein PF16 with mNeonGreen, as previously described.^29^

To introduce specific mutations into *CPSF*3, 3 × 10^6^ promastigotes expressing Cas9 and T7 RNA were resuspended in a 110 μL of Human T-Cell Nucleofector reagent and transfected with 18 μL of sgRNA and 4 μg of template using the Amaxa Nucleofector electroporator (program V-033). sgRNA was produced according to the previously established protocol from the Gluenz lab,^30^ in which the scaffold primer G00 and a gene-specific forward primer (FwsgRNA) were used (Table S1). The sgRNA template and templates introducing base edits are summarized in Table S1. Twenty-four hours following transfection, DNDI-6148 was added to cultures at 6 μM. Drug-selected populations were cloned by limiting dilution, EC_50s_ were determined, and genomic DNA was extracted. The *CPSF*3 gene from CRISPR-edited parasites was amplified by PCR, and the resulting products were sequenced.

### *In Vitro* Drug Sensitivity Assays (MoA Studies)

Drug sensitivity assays with wild-type and transgenic *T. brucei* and *L. donovani* cell lines were carried out as previously described.^31,32^

### Confirmation of CPSF3 Overexpression Using MS

WT and *L. donovani* promastigotes (3 × 10^8^) overexpressing CPSF3 were washed twice with PBS (1000 g, 4 °C, 5 min). The pellet was resuspended in 1.5 mL of lysis buffer (50 mM potassium phosphate buffer, pH 7.0, 1 mM EDTA, 1 mM DTT, and 1 × EDTA-free complete protease inhibitors (Roche)). Parasites were biologically inactivated by 3 × freeze/thaw cycles prior to lysis using a One-Shot Cell Disruptor (Constant Systems) at 30 kpsi. Lysed cells were then centrifuged (10 min, 4 °C, 15 000*g*), and the soluble material was harvested. Soluble cell lysates (100 μg samples) were reduced by the addition of tris(2-carboxyethyl)phosphine hydrochloride (TCEP, 25 mM final concentration) and incubated at 37 °C for 10 min. The samples were then alkylated by the addition of iodoacetamide (25 mM final concentration) and incubated at RT for 1 h in the dark. Alkylated samples were digested by the addition of 1:50 endoproteinase LysC (Wako, Japan) in 100 mM triethylammonium bicarbonate (TEAB), followed by incubation at 37 °C for 6 h, then the addition of 1:50 trypsin (1.50 dilution) and incubation at 37 °C overnight. The resulting digested peptides were then vacuum-dried.

### LC-MS/MS Analysis

Analysis of peptides was performed on a Q-Exactive Plus mass spectrometer (Thermo Scientific) coupled with a Dionex Ultimate 3000 RS (Thermo Scientific). LC buffers used were as follows: Buffer A (0.1% formic acid in Milli-Q water (v/v)) and Buffer B (80% acetonitrile and 0.1% formic acid in Milli-Q water (v/v)). Aliquots (15 μL per sample) were loaded at 10 μL/min onto a PepMap nanoViper C18 column (100 μm × 2 cm, 5 μm, 100 Å, Thermo Scientific) equilibrated with 98% Buffer A. The column was washed for 5 min at the same flow rate and then was switched in line with a Thermo Scientific, resolving PepMap RSLC C18 column (75 μm × 50 cm, 2 μm, 100 Å). Peptides were eluted at a constant flow rate of 300 nL/min with a linear gradient of 2–35% Buffer B in 125 min, then to 98% in 127 min. The column was then washed with 98% Buffer B for 20 min and re-equilibrated in 2% Buffer B for 17 min prior to loading the next sample. The Q-Exactive Plus was used in the data-dependent mode. The scan cycle comprised MS1 scan (*m*/*z* range from 335–1600, with a maximum ion injection time of 20 ms, a resolution of 70 000, and an automatic gain control (AGC) value of 1 × 106), followed by 15 sequential dependent MS2 scans (with an isolation window set to 1.4 Da, resolution at 17 500, maximum ion injection time at 100 ms, and AGC 2 × 105). The stepped collision energy was set to 27 and fixed first mass to 100 *m*/*z*. Spectra were acquired in a centroid mode and unassigned charge states. Charge states above 6, as well as singly charged species, were rejected. To ensure mass accuracy, the mass spectrometer was calibrated on day 1 of the analyses. LC-MS analysis was performed by the FingerPrints Proteomics Facility (University of Dundee).

### Data Analysis

MS data analysis was performed using MaxQuant software (http://maxquant.org/, version 1.6.2.6a). Carbamidomethyl (C), oxidation (M), acetyl (Protein N-term), deamidation (NQ), and Gln -> pyro-Glu were set as variable modifications. Proteins were identified by searching a protein sequence database containing *T. brucei brucei* TREU927 annotated proteins (downloaded from TriTrypDB 46, http://www.tritrypdb.org). LFQ and “match between runs” features were enabled. Trypsin/P and LysC/P were selected as the specific proteases with two potential missed cleavages. The FDR threshold for peptides and proteins was 0.01. FTMS MS/MS mass tolerance was set to 10 ppm, and ITMS MS/MS mass tolerance was 0.6 Da. Protein abundance was obtained using LFQ intensity values. LFQ intensities were calculated using at least 2 unique peptides. Data was visualized using Perseus 1.6.2.1 (https://maxquant.org/perseus/).

### Homology Modeling and *In Silico* Docking Studies

To identify suitable template structures for the generation of the *L. donovani* CPSF3 homology model, the sequence UniProtKB—E9BRB9 was used to query the PDB using “BLAST” as implemented in the NCBI blastp suite (https://blast.ncbi.nlm.nih.gov/). Endonucleases from five different species, human CPSF73 (PDB code 2I7T and 6M8Q—sequence identity 54%), *Saccharomyces cerevisiae* CPSF (PDB code 6I1D—sequence identity 47%), *Pyrococcus horikoshii* CPSF3 (PDB code 3AF5—sequence identity 29%), *Cryptosporidium hominis* (PDB code 6Q55—sequence identity 48%), and *T. thermophilus* TTHA0252 (several structures available exemplified by PDB code 3IEM—sequence identity 30%), were identified as close analogues that could be used as template structures. The *T. thermophilus* structure is complexed with a stable RNA analogue bound to the catalytic site located at the interface between the metallo-β-lactamase and β-CASP domains, whereas one human, *S. cerevisiae* and *P. horikoshii* structures are apo forms where the Zn^2+^-containing binding site is inaccessible. The *C. hominis* structure is in complex with a small boron-containing inhibitor (AN3661) and where the binding site is still inaccessible to a larger ligand. One of the human structures (6M8Q) is in complex with a small ligand that does not interact with the two Zn^2+^ ions and adopts the binding mode that causes structural rearrangements in loop regions adjacent to the binding cavity. Thus, the *T. thermophilus* structure was selected as a suitable template. The alignment between the amino acid sequence of *Ld*CPSF3 that of the *T. thermophilus* CPSF3 was manually curated (Figure S5) and showed a sequence identity of 68% in the binding site area. A homology model for *Ld*CPSF3 was built using the knowledge-based method in Prime in Schrödinger (Schrödinger Release 2019-3: Schrödinger, LLC, New York, NY, 2020). Zinc atoms were modeled into the structure but not the RNA substrate. Due to its empty p-orbital, the boron atom in DNDI-6148 is a strong electrophile (Lewis acid) that can react with solvent water molecules. The nucleophilic attack of an activated water molecule on the trigonal boron atom leads to the formation of a tetrahedral negatively charged boron species. The three-dimensional structure of the hydroxylated form of DNDI-6148 was built in Maestro, minimized with the OPLS3 force field, and docked in the catalytic site of the *Ld*CPSF3 model using GLIDE in Schrödinger. Docking results were subjected to a restrained minimization procedure with the OPLS3e force field, as implemented, where each heavy atom was allowed to deviate by up to 0.5 Å from its original position in the model.

## Supporting Information Available

Chemistry experimental details for all compounds, Supplementary figures and tables related to MoA and docking studies; HPLC chromatograms of key compounds used *in vivo*; and data illustrating the PK properties of compound **23** in rat and dog and off-target profiling of compound **23** (PDF)
String data for all compounds (CSV)
DNDI-6148 rat and dog PK data (XLSX)
Compound **23** docked into *Ld*CPSF3 (PDB)

## Supplementary Material

jm1c01437_si_003.xlsx

jm1c01437_si_004.pdb

## Abbreviations Used

CC_50_: half-maximum cytotoxic concentration
Cl_int_: intrinsic clearance
CPSF3: cleavage and polyadenylation specificity factor subunit 3
DND*i*: Drugs for Neglected Diseases *initiative*
FaSSIF: fasted state simulated intestinal fluid
FeSSIF: fed state simulated intestinal fluid
HLM: human liver microsomes
HamLM: hamster liver microsomes
LAB: liposomal amphotericin B
MoA: mode of action
MRC5: human fetal lung fibroblasts
PM: paromomycin
PMM: primary mouse macrophages
SI: selectivity index
SSG: sodium stibogluconate
TCP: target candidate profile
TPP: target product profile
VL: visceral leishmaniasis

## References

[ref1] Alvar J.; Yactayo S.; Bern C. Leishmaniasis and poverty. Trends Parasitol. 2006, 22, 552–557. 10.1016/j.pt.2006.09.004. 17023215

[ref2] Alvar J.; Vélez I. D.; Bern C.; Herrero M.; Desjeux P.; Cano J.; Jannin J.; den Boer M. Leishmaniasis worldwide and global estimates of its incidence. PLoS One 2012, 7, e35671 10.1371/journal.pone.0035671. 22693548 PMC3365071

[ref3] WHO Leishmaniasis fact sheet. https://www.who.int/news-room/fact-sheets/detail/leishmaniasis.

[ref4] Berman J. Visceral leishmaniasis in the New World & Africa. Indian J. Med. Res. 2006, 123, 289–294. 16778311

[ref5] DNDi Current treatments for leishmaniasis. https://dndi.org/diseases/visceral-leishmaniasis/facts/.

[ref6] Alves F.; Bilbe G.; Blesson S.; Goyal V.; Monnerat S.; Mowbray C.; Muthoni Ouattara G.; Pécoul B.; Rijal S.; Rode J.; Solomos A.; Strub-Wourgaft N.; Wasunna M.; Wells S.; Zijlstra E. E.; Arana B.; Alvar J. Recent Development of visceral leishmaniasis treatments: successes, pitfalls, and perspectives. Clin. Microbiol. Rev. 2018, 31, e00048-18 10.1128/CMR.00048-18. 30158301 PMC6148188

[ref7] Wyllie S.; Roberts A. J.; Norval S.; Patterson S.; Foth B. J.; Berriman M.; Read K. D.; Fairlamb A. H. Activation of bicyclic nitro-drugs by a novel nitroreductase (NTR2) in Leishmania. PLoS Pathog. 2016, 12, e1005971 10.1371/journal.ppat.1005971. 27812217 PMC5094698

[ref8] Wyllie S.; Thomas M.; Patterson S.; Crouch S.; De Rycker M.; Lowe R.; Gresham S.; Urbaniak M. D.; Otto T. D.; Stojanovski L.; Simeons F. R. C.; Manthri S.; MacLean L. M.; Zuccotto F.; Homeyer N.; Pflaumer H.; Boesche M.; Sastry L.; Connolly P.; Albrecht S.; Berriman M.; Drewes G.; Gray D. W.; Ghidelli-Disse S.; Dixon S.; Fiandor J. M.; Wyatt P. G.; Ferguson M. A. J.; Fairlamb A. H.; Miles T. J.; Read K. D.; Gilbert I. H. Cyclin-dependent kinase 12 is a drug target for visceral leishmaniasis. Nature 2018, 560, 192–197. 10.1038/s41586-018-0356-z. 30046105 PMC6402543

[ref9] Wyllie S.; Brand S.; Thomas M.; De Rycker M.; Chung C. W.; Pena I.; Bingham R. P.; Bueren-Calabuig J. A.; Cantizani J.; Cebrian D.; Craggs P. D.; Ferguson L.; Goswami P.; Hobrath J.; Howe J.; Jeacock L.; Ko E. J.; Korczynska J.; MacLean L.; Manthri S.; Martinez M. S.; Mata-Cantero L.; Moniz S.; Nühs A.; Osuna-Cabello M.; Pinto E.; Riley J.; Robinson S.; Rowland P.; Simeons F. R. C.; Shishikura Y.; Spinks D.; Stojanovski L.; Thomas J.; Thompson S.; Viayna Gaza E.; Wall R. J.; Zuccotto F.; Horn D.; Ferguson M. A. J.; Fairlamb A. H.; Fiandor J. M.; Martin J.; Gray D. W.; Miles T. J.; Gilbert I. H.; Read K. D.; Marco M.; Wyatt P. G. Preclinical candidate for the treatment of visceral leishmaniasis that acts through proteasome inhibition. Proc. Natl. Acad. Sci. U.S.A. 2019, 116, 9318–9323. 10.1073/pnas.1820175116. 30962368 PMC6511062

[ref10] Nagle A.; Biggart A.; Be C.; Srinivas H.; Hein A.; Caridha D.; Sciotti R. J.; Pybus B.; Kreishman-Deitrick M.; Bursulaya B.; Lai Y. H.; Gao M. Y.; Liang F.; Mathison C. J. N.; Liu X.; Yeh V.; Smith J.; Lerario I.; Xie Y.; Chianelli D.; Gibney M.; Berman A.; Chen Y. L.; Jiricek J.; Davis L. C.; Liu X.; Ballard J.; Khare S.; Eggimann F. K.; Luneau A.; Groessl T.; Shapiro M.; Richmond W.; Johnson K.; Rudewicz P. J.; Rao S. P. S.; Thompson C.; Tuntland T.; Spraggon G.; Glynne R. J.; Supek F.; Wiesmann C.; Molteni V. Discovery and characterization of clinical candidate LXE408 as a kinetoplastid-selective proteasome inhibitor for the treatment of leishmaniases. J. Med. Chem. 2020, 63, 10773–10781. 10.1021/acs.jmedchem.0c00499. 32667203 PMC7549094

[ref11] DNDi Acoziborole factsheet. https://dndi.org/research-development/portfolio/acoziborole/.

[ref12] Jacobs R. T.; Nare B.; Wring S. A.; Orr M. D.; Chen D.; Sligar J. M.; Jenks M. X.; Noe R. A.; Bowling T. S.; Mercer L. T.; Rewerts C.; Gaukel E.; Owens J.; Parham R.; Randolph R.; Beaudet B.; Bacchi C. J.; Yarlett N.; Plattner J. J.; Freund Y.; Ding C.; Akama T.; Zhang Y. K.; Brun R.; Kaiser M.; Scandale I.; Don R. SCYX-7158, an orally-active benzoxaborole for the treatment of stage 2 human African trypanosomiasis. PLoS Neglected Trop. Dis. 2011, 5, e1151 10.1371/journal.pntd.0001151. PMC3125149 21738803

[ref13] Nare B.; Wring S.; Bacchi C.; Beaudet B.; Bowling T.; Brun R.; Chen D.; Ding C.; Freund Y.; Gaukel E.; Hussain A.; Jarnagin K.; Jenks M.; Kaiser M.; Mercer L.; Mejia E.; Noe A.; Orr M.; Parham R.; Plattner J.; Randolph R.; Rattendi D.; Rewerts C.; Sligar J.; Yarlett N.; Don R.; Jacobs R. Discovery of novel orally bioavailable oxaborole 6-carboxamides that demonstrate cure in a murine model of late-stage central nervous system african trypanosomiasis. Antimicrob. Agents Chemother. 2010, 54, 4379–4388. 10.1128/AAC.00498-10. 20660666 PMC2944573

[ref14] Van den Kerkhof M.; Mabille D.; Chatelain E.; Mowbray C. E.; Braillard S.; Hendrickx S.; Maes L.; Caljon G. In vitro and in vivo pharmacodynamics of three novel antileishmanial lead series. Int. J. Parasitol.: Drugs Drug Resist. 2018, 8, 81–86. 10.1016/j.ijpddr.2018.01.006. 29425734 PMC6114106

[ref15] Hann M. M. Molecular obesity, potency and other addictions in drug discovery. In Multifaceted Roles of Crystallography in Modern Drug Discovery; Scapin G.; Patel D.; Arnold E., Eds.; Springer: Dordrecht, 2015; pp 183–196.

[ref16] Peterson L. A. Reactive metabolites in the biotransformation of molecules containing a furan ring. Chem. Res. Toxicol. 2013, 26, 6–25. 10.1021/tx3003824. 23061605 PMC3574180

[ref17] Wall R. J.; Moniz S.; Thomas M. G.; Norval S.; Ko E. J.; Marco M.; Miles T. J.; Gilbert I. H.; Horn D.; Fairlamb A. H.; Wyllie S. Antitrypanosomal 8-hydroxy-naphthyridines are chelators of divalent transition metals. Antimicrob. Agents Chemother. 2018, 62, e00235-18 10.1128/AAC.00235-18. 29844044 PMC6105827

[ref18] Begolo D.; Vincent I. M.; Giordani F.; Pöhner I.; Witty M. J.; Rowan T. G.; Bengaly Z.; Gillingwater K.; Freund Y.; Wade R. C.; Barrett M. P.; Clayton C. The trypanocidal benzoxaborole AN7973 inhibits trypanosome mRNA processing. PLoS Pathog. 2018, 14, e1007315 10.1371/journal.ppat.1007315. 30252911 PMC6173450

[ref19] Wall R. J.; Rico E.; Lukac I.; Zuccotto F.; Elg S.; Gilbert I. H.; Freund Y.; Alley M. R. K.; Field M. C.; Wyllie S.; Horn D. Clinical and veterinary trypanocidal benzoxaboroles target CPSF3. Proc. Natl. Acad. Sci. U.S.A. 2018, 115, 9616–9621. 10.1073/pnas.1807915115. 30185555 PMC6156652

[ref20] Sonoiki E.; Ng C. L.; Lee M. C.; Guo D.; Zhang Y. K.; Zhou Y.; Alley M. R.; Ahyong V.; Sanz L. M.; Lafuente-Monasterio M. J.; Dong C.; Schupp P. G.; Gut J.; Legac J.; Cooper R. A.; Gamo F. J.; DeRisi J.; Freund Y. R.; Fidock D. A.; Rosenthal P. J. A potent antimalarial benzoxaborole targets a Plasmodium falciparum cleavage and polyadenylation specificity factor homologue. Nat. Commun. 2017, 8, 14574 10.1038/ncomms14574. 28262680 PMC5343452

[ref21] Bellini V.; Swale C.; Brenier-Pinchart M. P.; Pezier T.; Georgeault S.; Laurent F.; Hakimi M. A.; Bougdour A. Target identification of an antimalarial oxaborole identifies AN13762 as an alternative chemotype for targeting CPSF3 in apicomplexan parasites. iScience 2020, 23, 101871 10.1016/j.isci.2020.101871. 33336164 PMC7733022

[ref22] Corpas-Lopez V.; Moniz S.; Thomas M.; Wall R. J.; Torrie L. S.; Zander-Dinse D.; Tinti M.; Brand S.; Stojanovski L.; Manthri S.; Hallyburton I.; Zuccotto F.; Wyatt P. G.; De Rycker M.; Horn D.; Ferguson M. A. J.; Clos J.; Read K. D.; Fairlamb A. H.; Gilbert I. H.; Wyllie S. Pharmacological validation of N-myristoyltransferase as a drug target in *Leishmania donovani*. ACS Infect. Dis. 2019, 5, 111–122. 10.1021/acsinfecdis.8b00226. 30380837 PMC6332449

[ref23] Van den Kerkhof M.; Leprohon P.; Mabille D.; Hendrickx S.; Tulloch L. B.; Wall R. J.; Wyllie S.; Chatelain E.; Mowbray C. E.; Braillard S.; Ouellette M.; Maes L.; Caljon G. Identification of resistance determinants for a promising antileishmanial oxaborole series. Microorganisms 2021, 9, 1408 10.3390/microorganisms9071408. 34210040 PMC8305145

[ref24] Van Bocxlaer K.; Caridha D.; Black C.; Vesely B.; Leed S.; Sciotti R. J.; Wijnant G. J.; Yardley V.; Braillard S.; Mowbray C. E.; Ioset J. R.; Croft S. L. Novel benzoxaborole, nitroimidazole and aminopyrazoles with activity against experimental cutaneous leishmaniasis. Int. J. Parasitol.: Drugs Drug Resist. 2019, 11, 129–138. 10.1016/j.ijpddr.2019.02.002. 30922847 PMC6904836

[ref25] Stauber L. A. Host Resistance to the Khartoum Strain of Leishmania donovani; The Rice University Pamphlet, 1958; Vol. 45, pp 80–96.

[ref26] Jha T. K.; Sundar S.; Thakur C. P.; Bachmann P.; Karbwang J.; Fischer C.; Voss A.; Berman J. Miltefosine, an oral agent, for the treatment of Indian visceral leishmaniasis. N. Engl. J. Med. 1999, 341, 1795–1800. 10.1056/NEJM199912093412403. 10588964

[ref27] Jacobs R. T. L. Y.; Sciotti R. J.; Robinson S. J.; Mowbray C. E.; Whitlock G. A.; Speake J. D.; Glossop P. A.; Launay D. F. M.. WO2018160845 - Novel oxaborole analogues and uses thereof. 2018.

[ref28] Wentrup C. W.; Winter H. W. Isolation of diazacycloheptatetraenes from thermal nitrene-nitrene rearrangements. J. Am. Chem. Soc. 1980, 102, 6159–6161. 10.1021/ja00539a039.

[ref29] Beneke T.; Madden R.; Makin L.; Valli J.; Sunter J.; Gluenz E. A CRISPR Cas9 high-throughput genome editing toolkit for kinetoplastids. R. Soc. Open Sci. 2017, 4, 170095 10.1098/rsos.170095. 28573017 PMC5451818

[ref30] Beneke T.; Gluenz E. LeishGEdit: A method for rapid gene knockout and tagging using CRISPR-Cas9. In Methods in Molecular Biology; Humana Press: New York, NY, 2019; Vol. 1971, pp 189–210. 30980304 10.1007/978-1-4939-9210-2_9

[ref31] Jones D. C.; Hallyburton I.; Stojanovski L.; Read K. D.; Frearson J. A.; Fairlamb A. H. Identification of a κ-opioid agonist as a potent and selective lead for drug development against human African trypanosomiasis. Biochem. Pharmacol. 2010, 80, 1478–1486. 10.1016/j.bcp.2010.07.038. 20696141 PMC3025325

[ref32] Wyllie S.; Patterson S.; Stojanovski L.; Simeons F. R.; Norval S.; Kime R.; Read K. D.; Fairlamb A. H. The anti-trypanosome drug fexinidazole shows potential for treating visceral leishmaniasis. Sci. Transl. Med. 2012, 4, 119re1 10.1126/scitranslmed.3003326. PMC3457684 22301556

