# Supplementary material for: DNDI-6148: A Novel Benzoxaborole Preclinical Candidate for the Treatment of Visceral Leishmaniasis
Source: J Med Chem. 2021 Oct 29;64(21):16159–76. doi: 10.1021/acs.jmedchem.1c01437 (PMC8591608; doi:10.1021/acs.jmedchem.1c01437)
Supplement: Supplementary file 1 — jm1c01437_si_001.pdf [file jm1c01437_si_001.pdf]

Supporting Information

DNDI-6148: A novel benzoxaborole preclinical candidate for the treatment of visceral leishmaniasis

Charles E. Mowbray[1,*], Stéphanie Braillard[1], Paul A. Glossop[2], Gavin A. Whitlock[2], Robert T. Jacobs[3], Jason Speake[3], Bharathi Pandi[3], Bakela Nare[3], Louis Maes[4], Vanessa Yardley[5], Yvonne Freund[6], Richard J. Wall[7], Sandra Carvalho[7], Davide Bello[7], Magali Van den Kerkhof[4], Guy Caljon[4], Ian H. Gilbert[7], Victoriano Corpas-Lopez[7], Iva Lukac[7], Stephen Patterson[7], Fabio Zuccotto[7], Susan Wyllie[7,*]

[1]Drugs for Neglected Diseases *initiative* (DND*i*), 15 Chemin Louis-Dunant, 1202 Geneva, Switzerland.

[2]Sandexis Medicinal Chemistry Ltd, Innovation House, Discovery Park, Ramsgate Road, Sandwich, Kent, CT13 9ND, UK.

[3]Scynexis, 3501 C Tricenter Boulevard, Durham, North Carolina 27713, USA

[4]Laboratory for Microbiology, Parasitology and Hygiene (LMPH), University of Antwerp, Universiteitsplein 1, 2610 Wilrijk, Antwerp, Belgium.

[5]London School of Hygiene & Tropical Medicine, Faculty of Infectious and Tropical Diseases, Keppel Street, London WC1E 7HT, UK

[6]Anacor Pharmaceuticals, 1020 East Meadow Circle, Palo Alto, California 94303, USA

[7]Division of Biological Chemistry and Drug Discovery, Wellcome Centre for Anti-infectives Research, School of Life Sciences, University of Dundee, Dow Street, Dundee DD1 5EH, UK.

[*]corresponding authors: Susan Wyllie (s.wyllie@dundee.ac.uk); Charles Mowbury (cmowbury@dndi.org)

Contents

Chemistry experimental S3

Supplementary figure and tables S46

Chemistry experimental

General procedure for the preparation of amides.

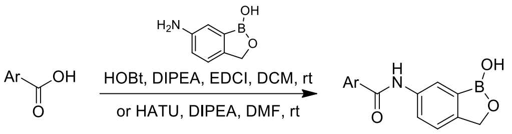

General procedure A. To a solution of the corresponding acid (14.4 mmol, 1.0 equiv), HOBt (17.3 mmol, 1.2 equiv), EDCI (17.3 mmol, 1.2 equiv) and DIPEA (36.1, 2.5 equiv) in DCM (30 mL) was added 6-aminobenzo[c][1,2]oxaborol-1(3H)-ol (14.4 mmol, 1.0 equiv). The reaction mixture was stirred at room temperature for 2 h. Water (100 mL) was added and the reaction mixture was extracted with DCM (100 mL x 3). The combined organic extracts were washed with brine (100 mL x 2), dried with anhydrous Na_2SO_4, filtered and concentrated in vacuum to give the crude product, which was purified by silica gel chromatography or preparative HPLC.

General procedure B. To a solution of the corresponding acid (1.5 mmol, 1.0 equiv), HATU (1.9 mmol, 1.3 equiv), DIPEA (2.8 mmol, 1.8 equiv) in DMF (3 mL) was added 6-aminobenzo[c][1,2]oxaborol-1(3H)-ol (**2**) (1.5 mmol, 1.0 equiv). The mixture was stirred at room temperature overnight. The mixture was purified by preparative HPLC.

General procedure C. A solution of the corresponding acid (10.5 mmol) in $SOCl_2$ (40 mL) was stirred at 50 °C for 4 h. The mixture was concentrated in vacuum to give acyl chloride which was used directly in the next step. To a solution of 6-aminobenzo[c][1,2]oxaborol-1(3H)-ol (**2**) (1.2 g, 8.1 mmol) and DIPEA (2.9 mL, 16.7

mmol) in THF (30 mL) was added the acid chloride (8.1 mmol). The mixture was stirred at room temperature for 16 h. Water (100 mL) was added and the reaction mixture was extracted with EtOAc (100 mL x 3). The combined organic extracts were washed with brine (100 mL x 2), dried with anhydrous Na_2SO_4, filtered and concentrated in vacuum to give the crude product, which was purified by silica gel chromatography or preparative HPLC.

N-(1-hydroxy-1,3-dihydrobenzo[c][1,2]oxaborol-6-yl)-1,3-bis(trifluoromethyl)-1H-pyrazole-4-carboxamide (4)

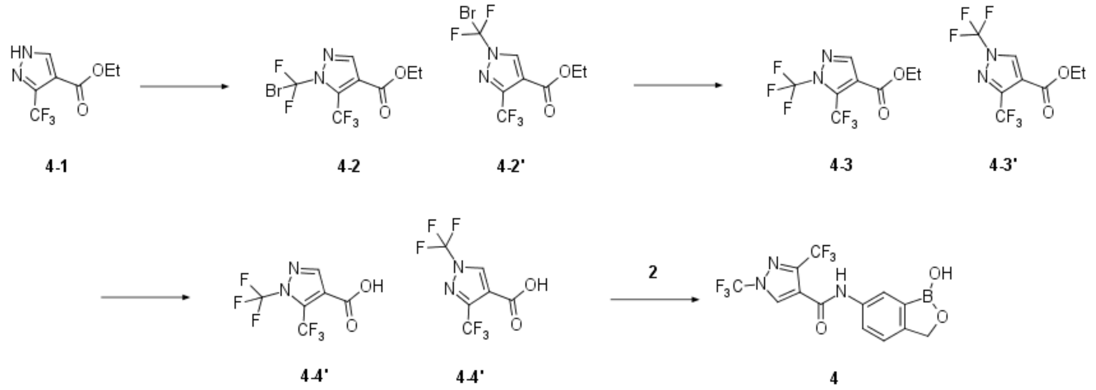

Ethyl 1-(bromodifluoromethyl)-3-(trifluoromethyl)-1H-pyrazole-4-carboxylate (4-2). To a solution of ethyl 3-(trifluoromethyl)-1H-pyrazole-4-carboxylate **4-1** (15.0 g, 72 mmol) in DMF (200 mL) was added NaH (3.7 g, 60% purity, 93.6 mmol) in portions at -10 ºC - 0 ºC. After stirring 30 min, dibromodifluoromethane (9.7 mL, 108 mmol) was added and then the mixture warmed to room temperature and stirred at this temperature overnight. The mixture was poured into ice-water (400 mL) and extracted with EtOAc (400 mL x 2). The organic layer was washed with brine (400 mL), dried

with anhydrous Na_2SO_4, filtered and concentrated in vacuum give the crude product, which was purified by silica gel chromatography (elution with Petroleum ether/Ethyl acetate = 50 : 1) to give a mixture of ethyl 1-(bromodifluoromethyl)-3-(trifluoromethyl)-1H-pyrazole-4-carboxylate and ethyl 1-(bromodifluoromethyl)-5-(trifluoromethyl)-1H-pyrazole-4-carboxylate (19.0 g, 79%) as colorless oil which was used directly in the next step.

Ethyl 1,3-bis(trifluoromethyl)-1H-pyrazole-4-carboxylate (4-3). To a mixture of ethyl 1-(bromodifluoromethyl)-3-(trifluoromethyl)-1H-pyrazole-4-carboxylate **4-2** and ethyl 1-(bromodifluoromethyl)-5-(trifluoromethyl)-1H-pyrazole-4-carboxylate **4-2'** (19.0 g, 57 mmol) in DCM (300 mL) was added $AgBF_4$ (33.3 g, 171 mmol) at -70 ºC to -65 ºC. The mixture warmed to room temperature over 2 h and stirred for another 16 h. Additional $AgBF_4$ (9.0 g, 46 mmol) was added at -70 ºC to -65 ºC and then the mixture warmed to room temperature and stirred at this temperature for 16 h. Saturated solution $NaHCO_3$ (200 mL) was added. The organic layer was washed with brine (200 mL), dried over anhydrous Na_2SO_4, concentrated in vacuum to give a mixture of ethyl 1,3-bis(trifluoromethyl)-1H-pyrazole-4-carboxylate and ethyl 1,5-bis(trifluoromethyl)-1H-pyrazole-4-carboxylate as yellow solid (13.5 g, crude) which was used directly in the next step.

1,3-bis(trifluoromethyl)-1H-pyrazole-4-carboxylic acid (4-4). To a mixture of ethyl 1,3-bis(trifluoromethyl)-1H-pyrazole-4-carboxylate **4-3** and ethyl 1,5-bis(trifluoromethyl)-1H-pyrazole-4-carboxylate **4-3'** (13.5 g, 49 mmol) in THF (100

mL) and H_2O (100 mL) was added LiOH (2.4 g, 100 mmol). The mixture was stirred at room temperature overnight. EtOAc (100 mL) was added and the phases were separated. The aqueous layer was acidified with 3 M HCl and extracted with EtOAc (100 mL x 2). The combined organic extracts were dried over anhydrous Na_2SO_4, concentrated in vacuum to give a mixture of 1,3-bis(trifluoromethyl)-1H-pyrazole-4-carboxylic acid and 1,5-bis(trifluoromethyl)-1H-pyrazole-4-carboxylic acid as yellow oil (9.2 g, crude) which was used directly in the next step.

_N_-(1-hydroxy-1,3-dihydrobenzo[c][1,2]oxaborol-6-yl)-1-(2,2,2-trifluoroethyl)-3-(trifluoromethyl)-1H-pyrazole-4-carboxamide (5)

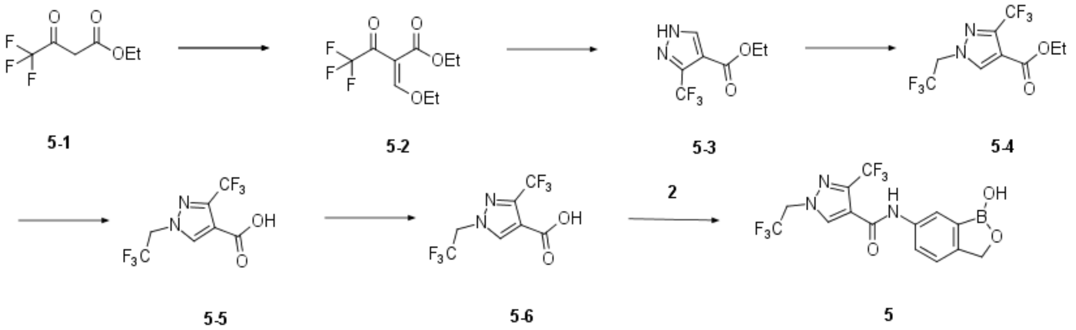

2-Ethoxymethylene-4,4,4-trifluoro-3-oxo-butyric acid ethyl ester (5-2). A mixture of 4,4,4-trifluoro-3-oxo-butyric acid ethyl ester **5-1** (200.0 g, 1.1 mol), triethoxymethane (346.0 g, 2.3 mol) in acetic anhydride (333.0 g, 3.3 mol) was heated to 140 °C for 7 h. After cooling to room temperature, the mixture was poured into ice-water (600 mL), extracted with DCM (250 mL x 3). The combined organic extracts were washed with brine (300 mL x 2), dried over anhydrous Na_2SO_4 and concentrated

to yield light yellow oil (228 g, crude) which was used directly in the next step.

3-Trifluoromethyl-1H-pyrazole-4-carboxylic acid ethyl ester (5-3). To an ice-cooled solution of 2-ethoxymethylene-4,4,4-trifluoro-3-oxo-butyric acid ethyl ester **5-2** (152.0 g, 633 mmol) in ethanol (1 L) was added hydrazine hydrate (32.2 g, 644 mmol). The mixture was stirred at room temperature for 16 h and then concentrated in vacuum to give the crude product which was purified by silica gel chromatography (elution with Petroleum ether/Ethyl acetate = 150 : 1 to 50 : 1) to yield a white solid (68.0 g, 52%). ^{1}H NMR (400MHz, DMSO-d6) δ 14.19 (s, 1H), 8.63 (s, 1H), 4.32 (q, *J* = 7.2 Hz, 2H), 1.34 (t, *J* = 7.2 Hz, 3H).

Ethyl 1-(2,2,2-trifluoroethyl)-3-(trifluoromethyl)-1H-pyrazole-4-carboxylate (5-4). To a solution of ethyl 3-(trifluoromethyl)-1H-pyrazole-4-carboxylate **5-3** (56.0 g, 269.2 mmol) and Cs_2CO_3 (262.5 g, 805.2 mmol) in MeCN (1 L) was added 2,2,2-trifluoroethyl trifluoromethanesulfonate (93.5 g, 403.0 mmol) drop-wise. The mixture was stirred at room temperature for 3 h. The mixture was concentrated in vacuum to give a residue which was diluted with water (150 mL) and extracted with EtOAc (100 mL x 3). The combined organic extracts were washed with water (80 mL x 3), brine (80 mL x 2), dried anhydrous Na_2SO_4, and concentrated in vacuum to yield t a yellow solid (66.0 g, crude) which was used directly in the next step.

1-(2,2,2-Trifluoroethyl)-3-(trifluoromethyl)-1H-pyrazole-4-carboxylic acid (5-5). To a solution of ethyl 1-(2,2,2-trifluoroethyl)-3-(trifluoromethyl)-1H-pyrazole -4-carboxylate **5-4** (66.0 g, 227.6 mmol) in EtOH (1.2 L) was added a solution of NaOH

(27.3 g, 682.5 mmol) in water (300 mL). The mixture was stirred at room temperature for 12 h. EtOH was removed under reduced pressure. The solution was washed with EtOAc (500 mL x 2), and the aqueous layer was adjusted pH to 3 with 4 M HCl. The precipitate was filtered and the cake was washed with water, dried under reduced pressure to yield a white solid (50.0 g, 84%).

1-(2,2,2-Trifluoroethyl)-3-(trifluoromethyl)-1H-pyrazole-4-carbonyl chloride (5-6). A solution of 1-(2,2,2-trifluoroethyl)-3-(trifluoromethyl)-1H-pyrazole -4-carboxylic acid **5-5** (40.0 g, 152.7 mmol) in sulfurous dichloride (150 mL) was heated to 50 °C and stirred for 4 h. The mixture was concentrated in vacuum to yield a yellow solid (42.7 g, crude) which was used directly in the next step.

***N*-(1-hydroxy-1,3-dihydrobenzo[c][1,2]oxaborol-6-yl)-3-(trifluoromethyl)-1-(3,3,3-trifluoropropyl)-1H-pyrazole-4-carboxamide (6)**

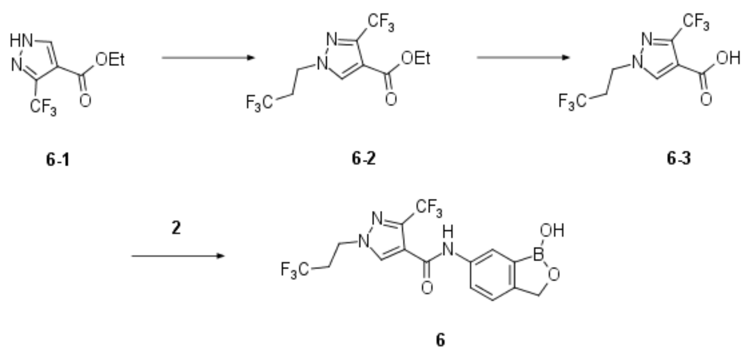

Ethyl 3-(trifluoromethyl)-1-(3,3,3-trifluoropropyl)-1H-pyrazole-4-carboxylate (6-2). To a suspension of ethyl 3-(trifluoromethyl)-1H-pyrazole-4-carboxylate 6**-1** (18.0 g, 86.5 mmol) and K_2CO_3 (23.9 g, 173.2 mmol) in DMF (200 mL) was added 3,3,3-

trifluoropropyl trifluoromethanesulfonate (51.1 g, 207.3 mmol) drop-wise at 5 °C and then the mixture was stirred at room temperature for 12 h. Water (500 mL) was added and the mixture was extracted with EtOAc (200 mL x 2). The combined organic extracts were washed with brine (400 mL x 3), dried over Na_2SO_4 and concentrated to yield yellow oil (31.0 g, crude) which was used directly in the next step. LC-MS (ESI) m/z = 305.0 $[M + 1]^+$, t = 0.822 min.

3-(Trifluoromethyl)-1-(3,3,3-trifluoropropyl)-1H-pyrazole-4-carboxylic acid (6-3). To a solution of ethyl 3-(trifluoromethyl)-1-(3,3,3-trifluoropropyl)pyrazole-4-carboxylate **6-2** (30.0 g, 98.7 mmol) in MeOH (100 mL) was added a solution of NaOH (27.6 g, 690.0 mmol) in water (100 mL). The mixture was stirred at room temperature for 12 h. MeOH was removed under reduced pressure. The pH was adjusted to 6 with 6 M HCl and the mixture was extracted with EtOAc (300 mL). The organic layer was dried over anhydrous Na_2SO_4, concentrated in vacuum to yield a yellow solid (25.0 g, crude) which was used directly in the next step.

***N*-(1-hydroxy-1,3-dihydrobenzo[c][1,2]oxaborol-6-yl)-3-methyl-1-(3,3,3-trifluoropropyl)-1H-pyrazole-4-carboxamide (7) and *N*-(1-hydroxy-1,3-dihydrobenzo[c][1,2]oxaborol-6-yl)-5-methyl-1-(3,3,3-trifluoropropyl)-1H-pyrazole-4-carboxamide (15)**

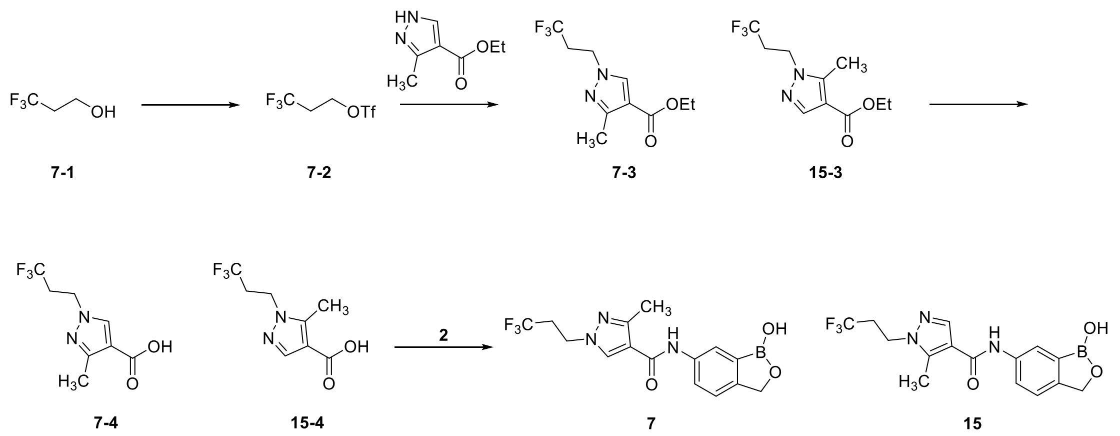

3,3,3-trifluoropropyl trifluoromethanesulfonate (7-2). To a solution of 3,3,3-trifluoropropan-1-ol **7-1** (5.0 g, 43.9 mmol) and DIPEA (8.5 g, 65.9 mmol) in DCM (50 mL) was added Tf_2O (14.8 g, 52.5 mmol) dropwise at 0 ºC. The mixture was stirred at room temperature overnight. The reaction mixture was washed with H_2O (30 mL x 3) and saturated $NaHCO_3$ aqueous solution (30 mL x 3). The organic layer was dried over Na_2SO_4 and concentrated in vacuum to give a yellow oil (4.8 g, 44%). ^{1}H NMR (400MHz, DMSO-d_6): δ 4.43 (t, J= 11.6 Hz, 2H), 2.73 (m, 2H).

Ethyl 3-methyl-1-(3,3,3-trifluoropropyl)-1H-pyrazole-4-carboxylate (7-3) and **ethyl 5-methyl-1-(3,3,3-trifluoropropyl)-1H-pyrazole-4-carboxylate (15-3).** To a mixture of ethyl 3-methyl-1H-pyrazole-4-carboxylate (1.0 g, 6.5 mmol) and K_2CO_3 (2.0 g, 14.5 mmol) in DMF (10 mL) was added 3,3,3-trifluoropropyl trifluoromethanesulfonate **7-2** (2.0 g, 8.1 mmol) drop-wise at 0 ºC. The mixture was stirred at room temperature overnight and diluted with H_2O (50 mL), extracted with MTBE (30 mL x 3). The combined organic extracts were combined and dried over Na_2SO_4, concentrated in vacuum to give a residue. The residue was purified by silica

gel chromatography (elution with PE : EA = 10 : 1 to 5 : 1) to give a yellow solid (521 mg, 32%) which were used directly in the next step without further separation.

3-Methyl-1-(3,3,3-trifluoropropyl)-1H-pyrazole-4-carboxylic acid (7-4) and **5-methyl-1-(3,3,3-trifluoropropyl)-1H-pyrazole-4-carboxylic acid (15-4).** To a solution of ethyl 3-methyl-1-(3,3,3-trifluoropropyl)-1H-pyrazole-4-carboxylate **7-3** and ethyl 5-methyl-1-(3,3,3-trifluoropropyl)-1H-pyrazole-4-carboxylate **15-3** (521 mg, 2.1 mmol) in THF (10 mL) was added LiOH aqueous (5 mL, 2 M, 10.0 mmol). The mixture was stirred at room temperature for 2 h. The reaction mixture was washed with MTBE (10 mL x 3), adjusted pH to 4 with 4 M HCl, extracted with EtOAc (10 mL x 3). The organic extracts were combined and dried over Na_2SO_4 concentrated in vacuum to give a white solid (469.0 mg, crude) which were used directly in the next step.

Cyclobutyl-*N*-(1-hydroxy-1,3-dihydrobenzo[c][1,2]oxaborol-6-yl)-3-methyl-1H-pyrazole-4-carboxamide (8)

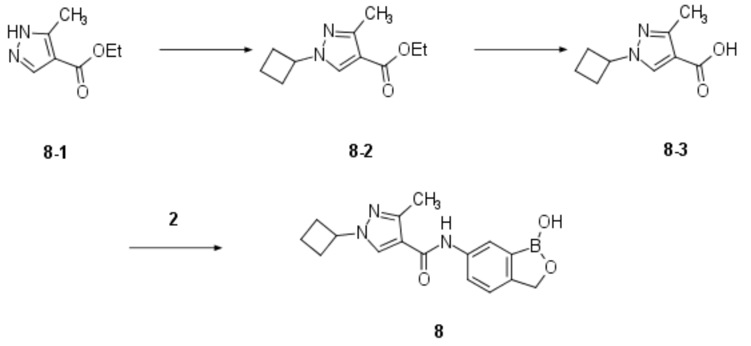

Ethyl 1-cyclobutyl-3-methyl-1H-pyrazole-4-carboxylate (8-2). To a solution of ethyl 3-methyl-1H-pyrazole-4-carboxylate **8-1** (1.0 g, 6.5 mmol) in DMF (10 mL) was added Cs_2CO_3 (4.2 g, 13.0 mmol) and bromocyclobutane (1.4 g, 9.5 mmol). The mixture was stirred at 100 ºC overnight. After cooling to room temperature, the reaction mixture was diluted with H_2O (50 mL), extracted with EtOAc (30 mL x 3) and the combined organic extracts were washed with brine (30 mL x 3), dried over Na_2SO_4 and concentrated in vacuum to give a residue which was purified by preparative TLC (Petroleum ether/Ethyl acetate = 10 : 1) to give a colourless oil (232.0 mg, 17%). ^{1}H NMR (400MHz, Methanol-d_4): δ 7.85 (s, 1H), 4.89 (m, 1H), 4.27 (q, J=7.2 Hz, 2H), 2.64 (m, 2H), 2.53 (s, 3H), 2.43 (m, 2H), 1.91 (m, 2H), 1.35 (t, J=7.2 Hz, 3H).

Cyclobutyl-3-methyl-1H-pyrazole-4-carboxylic acid (8-3). To a solution of ethyl 1-cyclobutyl-3-methyl-1H-pyrazole-4-carboxylate **8-2** (232.0 mg, 1.1 mmol) in MeOH (2 mL) and H_2O (6 mL) was added KOH (368.0 mg, 5.5 mmol). The mixture was stirred at 60 ºC overnight. The pH was adjusted to 5 with 4 M HCl and the mixture was extracted with EtOAc (10 mL x 3). The combined organic extracts was washed with brine (50 mL x 3), dried over Na_2SO_4 and concentrated in vacuum to give a white solid (201.0 mg, crude) which was used directly in the next step.

1-Cyclobutyl-*N*-(1-hydroxy-1,3-dihydrobenzo[c][1,2]oxaborol-6-yl)-3-(trifluoromethyl)-1H-pyrazole-4-carboxamide (9)

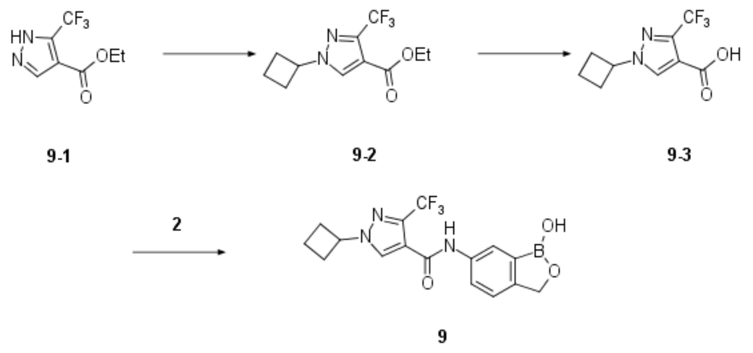

Ethyl 1-cyclobutyl-3-(trifluoromethyl)-1H-pyrazole-4-carboxylate (9-2). To a mixture of ethyl 5-(trifluoromethyl)-1H-pyrazole-4-carboxylate **9-1** (1.0 g, 4.8 mmol) and Cs_2CO_3 (2.4 g, 7.4 mmol) in DMF (15 mL) was added bromocyclobutane (1.4 g, 10.4 mmol). The mixture was stirred at 60 ºC overnight. After cooling to room temperature, water (40 mL) was added and the mixture was extracted with EtOAc (20 mL x 2). The combined organic extracts were washed with brine (40 mL x 3), dried over anhydrous Na_2SO_4, concentrated in vacuum to yield a yellow oil (1.4 g, crude) which was used directly in the next step. ^{1}H NMR (400MHz, DMSO-d$_6$) δ = 8.66 (s, 1H), 4.97 (m, 1H), 4.25 (q, J = 7.2 Hz, 2H), 2.48 (m, 4H), 1.81 (m, 2H), 1.27 (t, J = 7.2 Hz, 2H).

1-Cyclobutyl-3-(trifluoromethyl)-1H-pyrazole-4-carboxylic acid (9-3). To a solution of ethyl 1-cyclobutyl-3-(trifluoromethyl)-1H-pyrazole-4-carboxylate **9-2** (1.4 g, 5.3 mmol) in THF (4 mL) was added LiOH aqueous (6 mL, 2 M, 12 mmol) and the mixture was stirred at 60 ºC for 6 h. After cooling to room temperature, the mixture was concentrated under vacuum. Water (20 mL) was added and the mixture was extracted with EtOAc (10 mL x 3). The aqueous layer was adjusted to pH=2-3 and then extracted

with DCM (30 mL x 3). The combined organic extracts were washed with brine (100 mL), dried over anhydrous Na_2SO_4, concentrated in vacuum to yield a white solid (1.1 g, 89%). ^{1}H NMR (400MHz, DMSO-d_6) δ = 12.96 (s, 1H), 8.58 (s, 1H), 5.01 (m, 1H), 2.50 (m, 4H), 1.81 (m, 2H).

_N_-(1-hydroxy-1,3-dihydrobenzo[c][1,2]oxaborol-6-yl)-1-(tetrahydro-2H-pyran-4-yl)-3-(trifluoromethyl)-1H-pyrazole-4-carboxamide (10)

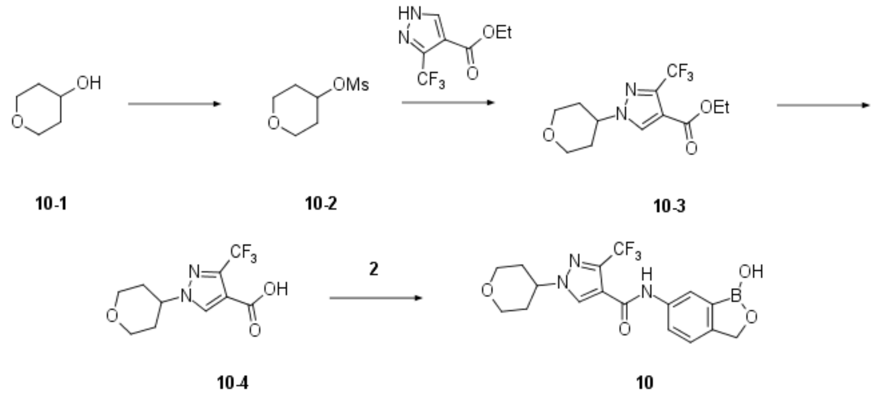

Cyclohexyl methanesulfonate (10-2). To a solution of cyclohexanol **10-1** (1.7 g, 16.7 mmol) in DCM (40 mL) and DIPEA (4.3 g, 33.3 mmol) was added MsCl (2.3 g, 20 mmol) drop-wise. The mixture was stirred at room temperature for 12 h. The mixture was diluted with H_2O (20 mL) and extracted with DCM (20 mL x 2). The combined organic layers were washed with saturated aqueous $NaHCO_3$ (40 mL x 2), brine(100 mL), dried with anhydrous Na_2SO_4, filtered and concentrated in vacuum to yield yellow oil (2.0 g, 67%). ^{1}H NMR (400MHz, DMSO-d_6) δ = 4.84 (m, 1H), 3.79 (m, 2H), 3.47 (m, 2H), 3.20 (s, 3H), 1.95 (m, 2H), 1.67 (m, 2H).

Ethyl 1-(tetrahydro-2H-pyran-4-yl)-3-(trifluoromethyl)-1H-pyrazole -4-

carboxylate (10-3). To a suspension of ethyl 3-(trifluoromethyl)-1H-pyrazole-4-carboxylate (582.0 mg, 2.8 mmol) and Cs_2CO_3 (2.7 g, 8.4 mmol) in DMF (20 mL) was added cyclohexyl methanesulfonate **10-2** (2.0 g, 11.1 mmol) drop-wise at 100 ºC and then the mixture was stirred at 100 ºC for 18 h. After cooling to room temperature, water (40 mL) was added and the reaction mixture was extracted with EtOAc (30 mL x 2). The combined organic extracts were washed with brine (60 mL x 2), dried with anhydrous Na_2SO_4, filtered and concentrated in vacuum to yield yellow oil (500 mg, crude) which was used directly in the next step.

1-(Tetrahydro-2H-pyran-4-yl)-3-(trifluoromethyl)-1H-pyrazole-4-carboxylic acid (10-4). To a solution of ethyl 1-(tetrahydro-2H-pyran-4-yl)-3-(trifluoromethyl)-1H-pyrazole-4-carboxylate **10-3** (860.0 mg, 2.9 mmol) in THF (10 mL) was added NaOH aqueous (4.8 mmol, 2.4 mL, 2 M). The mixture was stirred at room temperature for 16 h and then heated to 70 ºC for 12 h. The reaction was concentrated under reduced pressure to remove THF, and then EtOH (4 mL) was added. The mixture was stirred at room temperature for 6 h and then heated to 80 ºC for 8 h. The additional NaOH aqueous (7 mL, 2 M, 14 mmol) was added and the mixture was stirred at 90 ºC for 12 h. The mixture was extracted with EtOAc (20 mL). The aqueous layer was adjusted to pH = 2-3 with 2 M HCl and extracted with EtOAc (20 mL x 3). The combined organic extracts were dried with anhydrous Na_2SO_4, filtered and concentrated in vacuum to yield a white solid (900 mg, crude) which was used directly in the next step. ^1H NMR (400MHz, DMSO-d_6) δ = 8.52 (s, 1H), 4.54 (m, 1H), 3.97 (m, 2H), 3.47 (m, 2H), 1.98 (m, 4H).

N-(1-hydroxy-1,3-dihydro-2,1-benzoxaborol-6-yl)-3-methyl-1-(pyridine-2-yl)-1H-pyrazole-4-carboxamide (11)

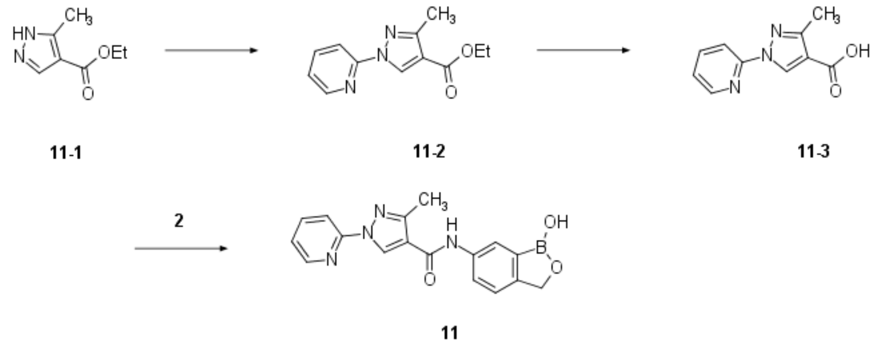

Ethyl 3-methyl-1-(pyridin-2-yl)-1*H*-pyrazole-4-carboxylate (11-2)

A round bottom flask was loaded with ethyl 3-methyl-1*H*-pyrazole-4-carboxylate **11-1** (700 mg, 4.59 mmol), potassium carbonate (1.26 g, 9.08 mmol), copper (I) chloride (45 mg, 0.45 mmol) and L-proline (105 mg, 0.910 mmol). The vessel was purged with nitrogen before the addition of anhydrous DMSO (5 ml). The reaction mixture was heated to 100 ºC overnight, diluted with EtOAc (30 ml) and filtered through a celite pad. The filtrate was washed with water, dried over $MgSO_4$, filtered and the solvent removed under reduced pressure to give the title compound (320 mg, 31%) as a crude residue used without further purification.

3-Methyl-1-(pyridin-2-yl)-1*H*-pyrazole-4-carboxylic acid (11-3)

Lithium hydroxide (100 mg 4.17 mmol) was added to a solution of ethyl 3-methyl-1-(pyridin-2-yl)-1*H*-pyrazole-4-carboxylate **11-2** (320 mg, 1.38 mmol) in a

THF/MeOH/water mixture (1:1:1) (6 ml). After stirring at room temperature for 4 h, the reaction was acidified to pH~2 with 1M HCl and extracted with EtOAc (2 x 15 ml). The combined organic layers were dried over $MgSO_4$, filtered, the solvent removed under reduced pressure to give the title compound (280 mg, 100%) as a white solid.

6-(((1-(Pyridin-2-yl)-3-(trifluoromethyl)-1H-pyrazole-4-carbonyl)oxy)amino)benzo[c][1,2]oxaborol-1(3H)-ol (12)

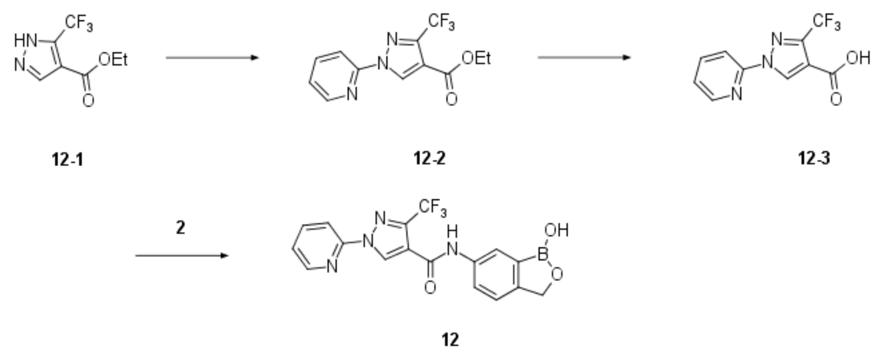

Ethyl 1-(pyridin-2-yl)-3-(trifluoromethyl)-1H-pyrazole-4-carboxylate (12-2). To a solution of ethyl 3-(trifluoromethyl)-1H-pyrazole-4-carboxylate **12-1** (500.0 mg, 2.4 mmol), 2-bromopyridine (376.8 mg, 2.4 mmol), CuI (136.8 mg, 0.72 mmol), K_2CO_3 (662.4 mg, 4.8 mmol), (1S,2S)-N1,N2-dimethylcyclohexane-1,2-diamine (204.5 mg, 1.4 mmol) in toluene (6 mL) was stirred at 110 ºC overnight. After cooling to room temperature, the mixture was filtered and the filtrate was diluted with water (5 mL) and extracted with EtOAc (5 mL x 2). The combined organic extracts were washed with brine (10 mL x 2), dried with anhydrous Na_2SO_4, filtered and concentrated in vacuum to give a yellow solid (600 mg, crude) which was used directly in the next step. ^{1}H

NMR (400MHz, DMSO-d_6): δ 9.19 (s, 1H), 8.60 (d, *J*=3.6 Hz, 1H), 8.13-8.03(m, 1H), 8.02 (d, *J*=8.4 Hz, 1H), 7.59-7.56 (m, 1H), 4.35-4.30 (m, 2H), 1.33 (t, *J*=7.2 Hz, 3H).

1-(Pyridin-2-yl)-3-(trifluoromethyl)-1H-pyrazole-4-carboxylic acid (12-3). To a solution of Ethyl 1-(pyridin-2-yl)-3-(trifluoromethyl)-1H-pyrazole-4-carboxylate **12-2** (600.0 mg, 2.1 mmol) in EtOH (10 mL) was added NaOH aqueous (4 mL, 2 M, 8.4 mol) at room temperature. The mixture was stirred at room temperature overnight. EtOH was removed under reduced pressure. EtOAc (5 mL) was added. The phase was separated and the aqueous layer was adjusted to pH=4. The precipitate was filtered and the cake was dried under reduced pressure to give a white solid (500.0 mg, 93%).^{1}H NMR (400MHz, DMSO-d_6) : δ 13.33 (s, 1H), 9.09 (m, 1H), 8.59 (m, 1H), 8.11 (m, 1H), 7.99 (d, *J*=8.2 Hz, 1H), 7.55 (m, 1H).

6-(((1-(4-chlorophenyl)-3-(trifluoromethyl)-1H-pyrazole-4-carbonyl)oxy)amino)benzo[c][1,2]oxaborol-1(3H)-ol (13)

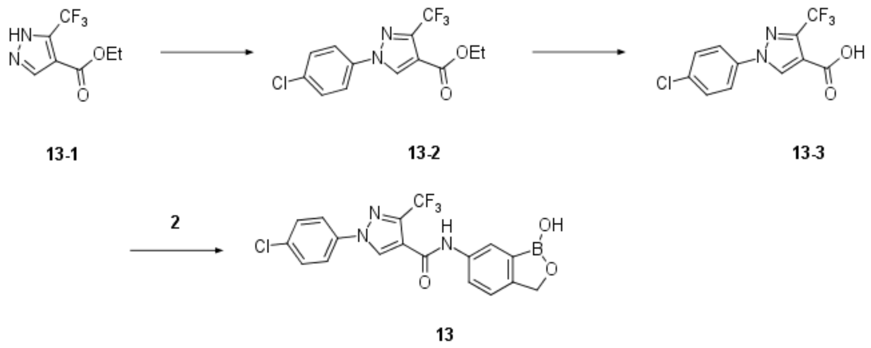

Ethyl 1-(4-chlorophenyl)-3-(trifluoromethyl)-1H-pyrazole-4-carboxylate (13-2). To a solution of ethyl 3-(trifluoromethyl)-1H-pyrazole-4-carboxylate **13-1** (500.0 mg,

2.4 mmol) in DMF (10 mL) was added (4-chlorophenyl)boronic acid (750.0 mg, 4.8 mmol), $Cu(OAc)_2$ (348.0 mg, 1.9 mmol), Py (0.39 mL, 4.8 mmol). The mixture was stirred at room temperature overnight under O_2 atmosphere. The saturated aqueous NH_4Cl (30 mL) and EtOAc (30 mL) were added. The phase was separated and the aqueous phase was extracted with EtOAc (30 mL x 2). The combined organic extracts were dried over anhydrous Na_2SO_4, concentrated in vacuum, and purified silica gel chromatography (elution with Petroleum ether/Ethyl acetate = 10 : 1 to 3: 1) to yield a white solid (764.0 mg, 76%). ^{1}H NMR (400MHz, CDCl3): δ 8.46 (s, 1H), 7.66(m, 2H), 7.47(m, 2H), 4.37 (q, *J*=7.2 Hz, 1H), 1.38 (t, *J*=7.2 Hz, 2H).

1-(4-Chlorophenyl)-3-(trifluoromethyl)-1H-pyrazole-4-carboxylic acid (13-3). To a solution of ethyl 1-(4-chlorophenyl)-3-(trifluoromethyl)-1H-pyrazole-4-carboxylate **13-2** (400.0 mg, 1.3 mmol) in THF (5 mL) was added NaOH aqueous (1.3 mL, 2M, 2.6 mmol). The mixture was stirred at room temperature overnight. The pH was adjusted to 6 and the mixture was extracted with EtOAc (10 mL x 2). The combined organic extracts were dried over anhydrous Na_2SO_4, concentrated in vacuum to yield a white solid (300.0 mg, 82%). ^{1}H NMR (400MHz, Methanol-d_4): δ 8.81 (d, *J*=0.4 Hz, 1H), 7.77 (m, 2H), 7.46 (m, 2H).

***N*-(1-hydroxy-1,3-dihydro-2,1-benzoxaborol-6-yl)-1-(2-methoxyphenyl)-3-methyl-1H-pyrazole-4-carboxamide (14)**

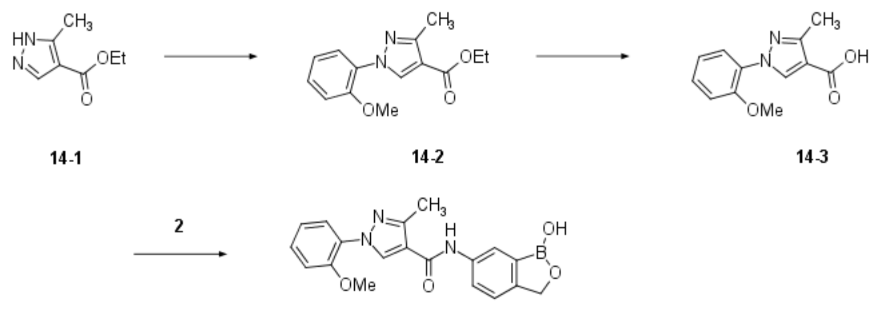

Ethyl 1-(2-methoxyphenyl)-3-methyl-1*H*-pyrazole-4-carboxylate (14-2)

To a solution of ethyl 3-methyl-1*H*-pyrazole-4-carboxylate **14-1** (700 mg, 4.54 mmol) in DCM (5 ml) were added 2-methoxybenzeneboronic acid pinacol ester (2.13 g, 9.08 mmol), copper (II) acetate (1.24 g, 6.81 mmol) and pyridine (1.44 g, 18.2 mmol). The reaction mixture was stirred at room temperature under air overnight. The mixture was filtered, washed with water (2 x 10 ml), saturated NaCl (15 ml), dried over $MgSO_4$, filtered, and fused onto silica (0.2 g). Purification by flash column chromatography (5 g SiO_2, ethyl acetate/hexanes 3:97 increasing to 25:75) gave the title compound (800 mg, 68%) as a light-yellow oil.

1-(2-Methoxyphenyl)-3-methyl-1*H*-pyrazole-4-carboxylic acid (14-3)

Lithium hydroxide (324 mg, 13.5 mmol) was added to a solution of ethyl 1-(2-methoxyphenyl)-3-methyl-1*H*-pyrazole-4-carboxylate **14-3** (880 mg, 3.38 mmol) in THF/MeOH/water mixture (1:1:1) (5 ml) in a 25 ml round bottom flask. After stirring at room temperature overnight, the reaction was acidified to pH~2 with 1 M HCl and extracted with EtOAc (2 x 15 ml). The combined organic layers were dried over $MgSO_4$,

filtered, and the solvents were removed under reduced pressure to give the title compound (600 mg, 76%) as an off-white solid.

***N*-(1-hydroxy-1,3-dihydrobenzo[c][1,2]oxaborol-6-yl)-5-methyl-1-(pyridin-2-yl)-1H-pyrazole-4-carboxamide (16)**

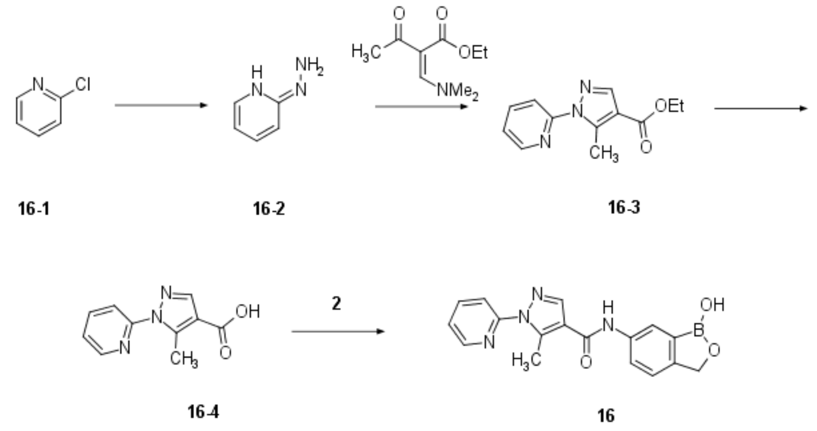

2-Hydrazono-1,2-dihydropyridine (16-2). A solution of 2-chloropyridine **16-1** (50.0 g, 0.44 mol) in hydrazine hydrate (200 mL, 98% purity) was stirred at 100 °C overnight. After cooling to room temperature, the reaction was diluted with H_2O (200 mL), extracted with EtOAc (300 mL x 3). The combined organic extracts were dried over Na_2SO_4, concentrated in a vacuum to give a yellow oil (42 g, 87 %).

Ethyl 5-methyl-1-(pyridin-2-yl)-1H-pyrazole-4-carboxylate (16-3). To a solution of 2-hydrazono-1,2-dihydropyridine **16-2** (42.0 g, 0.4 mol) in EtOH (400 mL) was added ethyl 2-((dimethylamino)methylene)-3-oxobutanoate (85.0 g, 0.5 mol). The mixture

was stirred at 80 °C for 2 h. The mixture was concentrated in vacuum to give a residue which was purified silica gel chromatography (elution with Petroleum ether/Ethyl acetate = 10 : 1) to give a yellow solid (60.0 g, 67%).

5-Methyl-1-(pyridin-2-yl)-1H-pyrazole-4-carboxylic acid (16-4). To a solution of 2-hydrazono-1,2-dihydropyridine **16-3** (60 g, 0.26 mol) in THF(120 mL) was added LiOH aqueous (500 mL, 3.5 M, 1.8 mol). The mixture was stirred at room temperature for 2 h. The reaction mixture was washed with MTBE (300 mL x 2), adjusted pH to 3 with 4 M HCl, extracted with EtOAc (40 mL x 3). The organic extracts were combined, dried over Na_2SO_4 and concentrated in vacuum to give a white solid (31.0 g, 58 %).

***N*-(1-hydroxy-1,3-dihydrobenzo[c][1,2]oxaborol-6-yl)-1-(6-methoxypyridin-2-yl)-5-methyl-1H-pyrazole-4-carboxamide (17)**

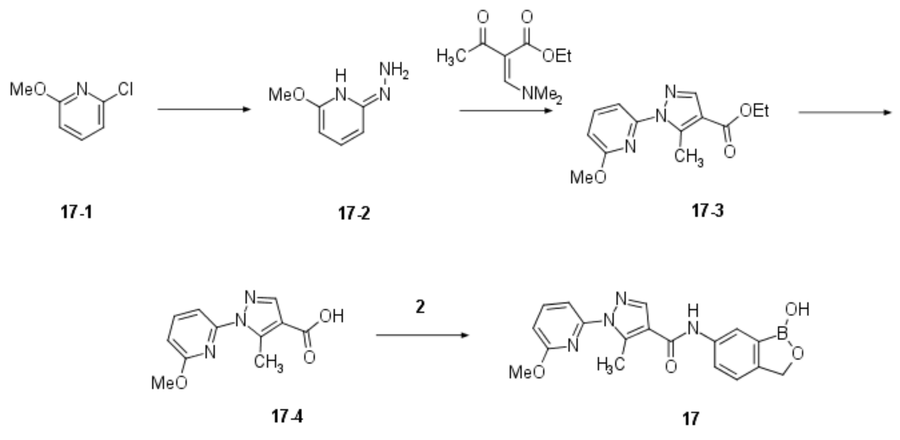

2-Hydrazono-6-methoxy-1,2-dihydropyridine (17-2). Compound **17-2** was

synthesized according to the same procedure as preparing of **16-2** using 2-chloro-6-methoxypyridine (19.0 g, 101 mmol) and hydrazine hydrate (65 mL, 98% purity) to yield a yellow oil (11.6 g, 82%) which was used directly in the next step. ^{1}H NMR (400MHz, $CDCl_3$) δ 7.43 (t, *J*=8.0 Hz, 1H), 6.24 (d, *J*=7.6 Hz, 1H), 6.13 (d, *J*=8.0 Hz, 1H), 5.76 (s, 1H), 3.88 (s, 3H), 3.88 (m, 2H).

Ethyl 1-(6-methoxypyridin-2-yl)-5-methyl-1H-pyrazole-4-carboxylate (17-3). Compound **17-3** was synthesized according to the same procedure as preparing of **16-3** using 2-hydrazono-6-methoxy-1,2-dihydropyridine (11.6 g, 83.4 mmol), ethyl 2-((dimethylamino)methylene)-3-oxobutanoate (27.2 g, 147.0 mmol) and EtOH (100 mL) to yield a yellow solid (18.1 g, 83%). ^{1}H NMR (400MHz, $CDCl_3$) δ 8.02 (s, 1H), 7.73 (t, *J*=8.0 Hz, 1H), 7.42 (d, *J*=7.6 Hz, 1H), 6.74 (d, *J*=8.0 Hz, 1H), 4.34 (q, *J*=7.2 Hz, 2H), 3.96 (s, 3H), 2.98 (s, 3H), 1.39 (t, *J*=7.2 Hz, 3H).

1-(6-Methoxypyridin-2-yl)-5-methyl-1H-pyrazole-4-carboxylic acid (17-4). Compound **17-4** was synthesized according to the same procedure as preparing of **16-4** using 1-(6-methoxypyridin-2-yl)-5-methyl-1H-pyrazole-4-carboxylate (9.2 g, 35.2 mmol), NaOH aqueous (27.5 mL, 2 M, 55.0 mmol) and THF (80 mL) to yield a white solid (7.3 g, 89%). ^{1}H NMR (400MHz, DMSO-d_6) δ 12.54 (s, 1H), 8.01 (s, 1H), 7.92 (t, *J*=8.0 Hz, 1H), 7.42 (d, *J*=7.6 Hz, 1H), 6.88 (d, *J*=8.0 Hz, 1H), 3.91 (s, 3H), 2.90 (s, 3H).

***N*-(1-hydroxy-1,3-dihydrobenzo[c][1,2]oxaborol-6-yl)-5-methyl-1-(6-**

methylpyridin-2-yl)-1H-pyrazole-4-carboxamide (18)

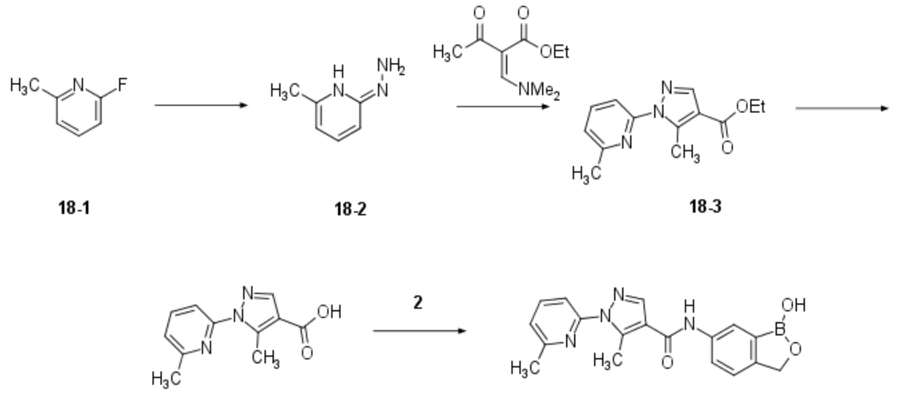

18-1 18-2 18-3 18-4 18

2-Hydrazono-6-methyl-1,2-dihydropyridine (18-2). Compound **18-2** was synthesized according to the same procedure as preparing of **16-2** using 2-fluoro-6-methylpyridine **18-1** (1.0 g, 9.0 mmol) and hydrazine hydrate (5 mL, 98% purity) to yield a yellow oil (1.1 g, crude) which was used directly in the next step.

Ethyl 5-methyl-1-(6-methylpyridin-2-yl)-1H-pyrazole-4-carboxylate (18-3). Compound **18-3** was synthesized according to the same procedure as preparing of **16-3** using 2-hydrazono-6-methyl-1,2-dihydropyridine **18-2** (800.0 mg, 6.5 mmol) and ethyl 2-((dimethylamino)methylene)-3-oxobutanoate (2.2 g, 12.0 mmol) to yield a yellow solid (500.0 mg, 31 %).^{1}H NMR (400MHz, $CDCl_3$): δ 8.04 (s, 1H), 7.76 (t, *J*=7.6 Hz, 1H), 7.59 (d, *J*=8.4 Hz, 1H), 7.17 (d, *J*=7.2 Hz, 1H), 4.37 (q, *J*=7.2 Hz, 2H), 2.92 (s, 3H), 2.61 (s, 3H), 1.40 (t, *J*=6.8 Hz, 3H).

5-Methyl-1-(6-methylpyridin-2-yl)-1H-pyrazole-4-carboxylic acid (18-4). Compound **18-4** was synthesized according to the same procedure as preparing of **16-4** using Ethyl 5-methyl-1-(6-methylpyridin-2-yl)-1H-pyrazole-4-carboxylate **18-3**

(500.0 mg, 2.0 mmol), NaOH aqueous (4 mL, 1 M, 4.0 mmol) and THF (10 mL) to yield a yellow solid (265.0 mg, 60 %) which was used directly the next step.

***N*-(1-hydroxy-1,3-dihydrobenzo[c][1,2]oxaborol-6-yl)-3-(pyridin-2-yl)-1H-pyrazole-5-carboxamide (19)**

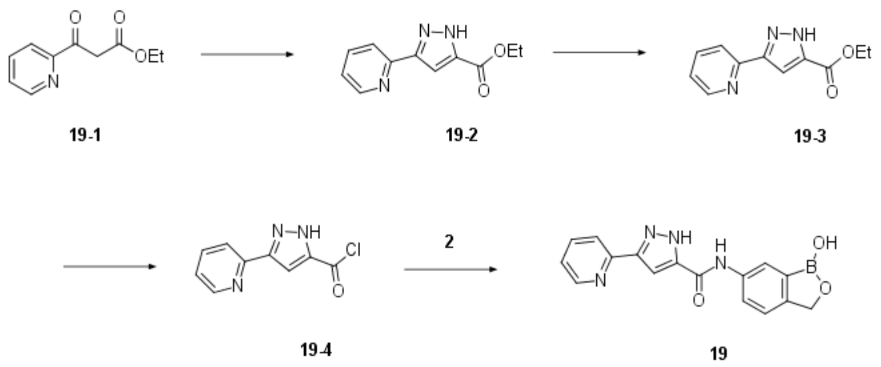

Ethyl 3-(pyridin-2-yl)-1H-pyrazole-5-carboxylate (19-2). Compound **19-2** was synthesized according to the same procedure as preparing of **20-2/21-2** using ethyl 2,4-dioxo-4-(pyridin-2-yl)butanoate **19-1** (500.0 mg, 2.3 mmol), hydrazine hydrate (113.0 mg, 2.3 mmol) and EtOH (5 mL) to yield the crude product (490 mg, crude) which was used directly in the next step.

3-(Pyridin-2-yl)-1H-pyrazole-5-carboxylic acid (19-3). Compound **19-3** was synthesized according to the same procedure as preparing of **20-4/21-4** using ethyl 3-(pyridin-2-yl)-1H-pyrazole-5-carboxylate **19-2** (490.0 mg, 2.3 mmol), KOH (632 mg, 11.3 mmol) H_2O (10 mL) and MeOH (10 mL) to yield a white solid (500 mg, crude) which was used directly in the next step.

3-(Pyridin-2-yl)-1H-pyrazole-5-carbonyl chloride (19-4). Compound **19-4** was synthesized according to the same procedure as preparing of **31-5** using 3-(pyridin-2-yl)-1H-pyrazole-5-carboxylic acid **19-3** (500.0 mg, 2.6 mmol), oxalyl dichloride (650.0 mg, 5.2 mmol), catalytic amount of DMF and DCM (10 mL) to yield a yellow solid (500.0 mg, crude) which was used directly in the next step.

***N*-(1-hydroxy-1,3-dihydrobenzo[c][1,2]oxaborol-6-yl)-1-methyl-3-(pyridin-2-yl)-1H-pyrazole-5-carboxamide (20) and *N*-(1-hydroxy-1,3-dihydrobenzo[c][1,2]oxaborol-6-yl)-1-methyl-5-(pyridin-2-yl)-1H-pyrazole-3-carboxamide (21)**

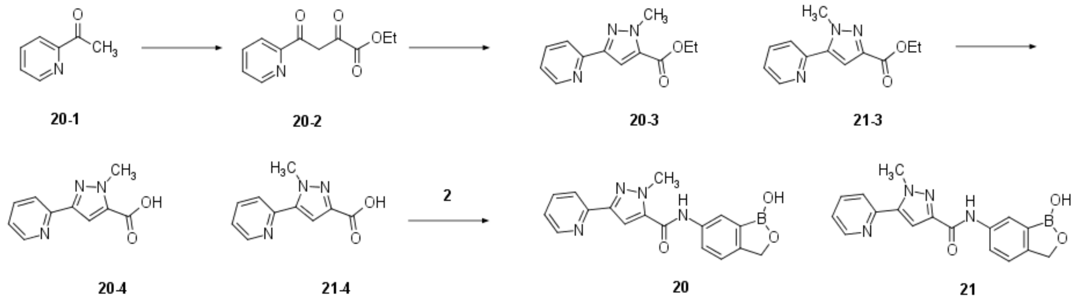

Ethyl 2,4-dioxo-4-(pyridin-2-yl)butanoate (20-1). To a solution of NaOMe (1.6 g, 29.6 mmol) in EtOH (30 mL) was added diethyl oxalate (4.3 g, 29.6 mmol), and then 1-(pyridin-2-yl)ethanone (3.0 g, 24.8 mmol) was added. The mixture was stirred at room temperature for 20 h. The saturated aqueous NH_4Cl (200 mL) and EtOAc (200 mL) were added. The phase was separated and the aqueous phase was extracted with

EtOAc (200 mL x 2). The combined organic extracts were dried over anhydrous Na_2SO_4, concentrated in vacuum and purified silica gel chromatography (elution with PE/EtOAc= 1 : 0 to 10 : 1) to yield a white solid (1.7 g, 31%). LC-MS (ESI) m/z = 222.2 $[M + 1]^+$, t = 0.680 min.

Ethyl 1-methyl-3-(pyridin-2-yl)-1H-pyrazole-5-carboxylate (20-2) and ethyl 1-methyl-5-(pyridin-2-yl)-1H-pyrazole-3-carboxylate (21-2). To a solution of ethyl 2,4-dioxo-4-(pyridin-2-yl)butanoate **20-1** (1.0 g, 4.5 mmol) in EtOH (10 mL) was added methylhydrazine (210.0 mg, 4.6 mmol). The mixture was stirred at 90 ºC for 8 h. After cooling to room temperature, the mixture was concentrated in vacuum to give a yellow solid (1.0 g, crude) which was used directly in the next step. LC-MS (ESI) m/z = 232.1 $[M + 1]^+$, t = 0.709 min and t = 0.800 min.

1-Methyl-3-(pyridin-2-yl)-1H-pyrazole-5-carboxylic acid (20-3) and 1-methyl-5-(pyridin-2-yl)-1H-pyrazole-3-carboxylic acid (21-3). To a solution of KOH (1.2 g, 21.4 mmol) in H_2O (20 mL) and MeOH (20 mL) was added a mixture of ethyl 1-methyl-3-(pyridin-2-yl)-1H-pyrazole-5-carboxylate **20-2** and ethyl 1-methyl-5-(pyridin-2-yl)-1H-pyrazole-3-carboxylate **21-2** (1.0 g, 4.3 mmol). The mixture was stirred at 50 ºC for 20 h. After cooling to room temperature, MeOH was removed under reduced pressure. The pH was adjusted to 3 with 2 M HCl. A lot of solid formed. The precipitate was filtered and dried in vacuum to give a white solid (700 mg, crude) which was used directly in the next step.

***N*-(1-hydroxy-1,3-dihydrobenzo[c][1,2]oxaborol-6-yl)-1-(pyridin-2-yl)-1H-1,2,3-triazole-4-carboxamide (22)**

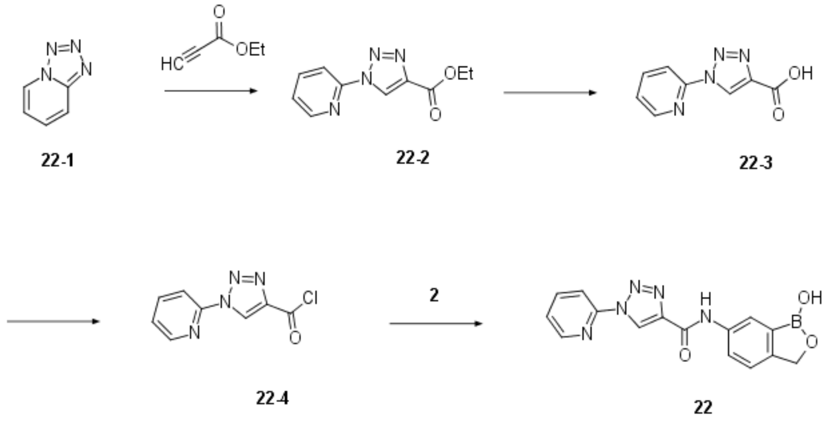

Ethyl 1-(pyridin-2-yl)-1H-1,2,3-triazole-4-carboxylate (22-2). To a suspension of tetrazolo[1,5-a]pyridine **22-1** (2.4 g, 20.0 mmol) in toluene (100 mL) and H_2O (1 mL) was added $Cu(CF_3SO_3)_2$ (1.4 g, 4.0 mmol), Na-ascrobate (1.2 g) and ethyl propiolate (2.3 g, 24.0 mmol) was added. The reaction was stirred at refluxing for 4 h. The mixture was concentrated in vacuum and purified silica gel chromatography (elution with Petroleum ether/Ethyl acetate= 5 : 1 to 2 : 1) to give a yellow solid (3.9 g, 89%). ^{1}H NMR (400MHz, DMSO-d_6): δ 9.30 (s, 1H), 8.65 (dd, J=4.8, 1.6 Hz, 1H), 8.16 (m, 2H), 7.62 (m, 1H), 4.36 (q, J=7.2 Hz, 2H), 1.34 (t, J=7.2 Hz, 3H).

1-(Pyridin-2-yl)-1H-1,2,3-triazole-4-carboxylic acid (22-3). To a solution of Ethyl 1-(pyridin-2-yl)-1H-1,2,3-triazole-4-carboxylate **22-2** (4.4 g, 20.4 mmol) in EtOH (120 mL) was added NaOH aqueous (10 mL, 6 M, 60 mmol). The reaction mixture was stirred at room temperature overnight. The solvent was evaporated and diluted with

water (100 mL). The aqueous layer was washed with EtOAc (50 mL). The aqueous phase was acidified with 3 M HCl. The precipitate filtered and the cake was dried under reduced pressure to yield a yellow solid (3.7 g, 97%). ^{1}H NMR (400MHz, DMSO-d_6): δ 13.43 (s, 1H), 9.20 (s, 1H), 8.64 (d, J=4.8 Hz, 1H), 8.16 (d, J=3.2 Hz, 2H), 7.61 (d, J=8.8, 4.4 Hz, 1H).

1-(Pyridin-2-yl)-1H-1,2,3-triazole-4-carbonyl chloride (22-4). A solution of 1-(pyridin-2-yl)-1H-1,2,3-triazole-4-carboxylic acid **22-3** (2.0 g, 10.5 mmol) in sulfurous dichloride (40 mL) was heated to 60 °C and stirred for 4 h. The solvent was removed under reduced pressure. The residue was added toluene (40 mL) and concentrated in vacuum yield a yellow solid (2.2 g, crude) which was used directly in the next step.

***N*-(1-hydroxy-1,3-dihydrobenzo[c][1,2]oxaborol-6-yl)-5-methyl-1-(pyridin-2-yl)-1H-1,2,3-triazole-4-carboxamide (23)**

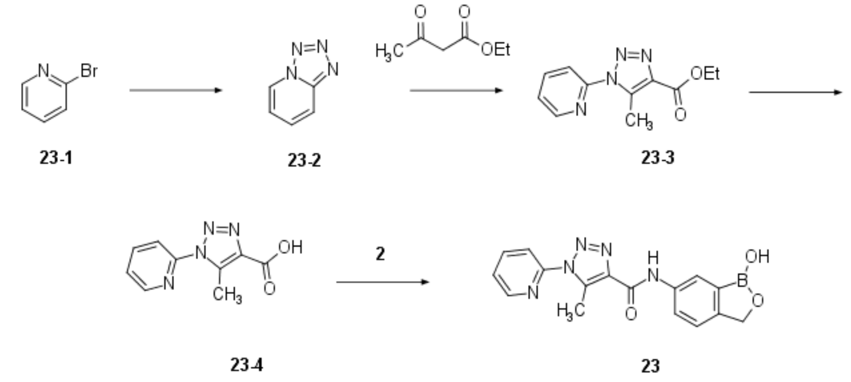

Tetrazolo[1,5-a]pyridine (23-2). A solution of 2-bromopyridine **23-1** (25.0 g, 159.2 mmol) in H_2O (225 mL) and EtOH (525 mL) was degassed and then sodium azide (31.5

g, 484.6 mmol), copper(I) iodide (3.0 g, 15.8 mmol), sodium ascorbate (1.6 g, 7.9 mmol) and DMEDA (2.1 g, 24.1 mmol) were added. The suspension was stirred at refluxing for 1 h. After cooling to room temperature, H_2O (1 L) was added and the pH was adjusted to 9. The mixture was extracted with DCM (1 L x 4) and the combined organic extracts was washed with brine (1 L), dried over Na_2SO_4, filtered and concentrated in vacuum to give a residue which was purified silica gel chromatography (elution with Petroleum ether/Ethyl acetate = 10 : 1) to yield a grey solid. (18.0 g, 94%). ^{1}H NMR (400 MHz, DMSO-d_6): δ = 9.31 (d, J=6.8 Hz, 1H), 8.21 (d, J=9.2 Hz, 1H), 7.86 (m, 1H), 7.44 (t, J=6.8 Hz, 1H).

5-Methyl-1-(pyridin-2-yl)-1H-1,2,3-triazole-4-carboxylic acid (23-4). To a solution of Tetrazolo[1,5-a]pyridine **23-2** (20.5 g, 170.8 mmol) and ethyl 3-oxobutanoate (44.4 g, 341.5 mmol) in EtOH (600 mL) was added fresh EtONa (34.0 g, 521.7 mmol). The mixture was stirred at refluxing for 48 h to give ethyl- 5-methyl-1-(pyridin-2-yl)-1H-1,2,3-triazole-4-carboxylate **23-3**. After cooling to room temperature, water (120 mL) was added and the mixture was stirred at 45 °C for 20 h. The solvent was evaporated and diluted with water (800 mL). The aqueous layer was washed with DCM (200 mL x 5). The aqueous phase was acidified with 3 M HCl. The precipitate filtered and the cake was dried under reduced pressure to yield a yellow solid (16.8 g, 48%). ^{1}H NMR (400 MHz, DMSO-d_6): δ 13.26 (s, 1H), 8.67 (m, 1H), 8.16 (m, 1H), 7.94 (d, J=8.0 Hz, 1H), 7.65(m, 1H), 2.74 (s, 3H).

***N*-(1-hydroxy-1,3-dihydrobenzo[c][1,2]oxaborol-6-yl)-5-methyl-1-phenyl-1H-1,2,3-triazole-4-carboxamide (24)**

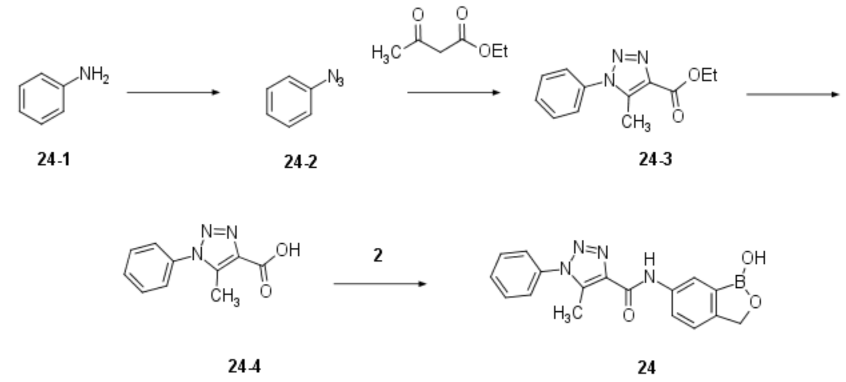

Azidobenzene (24-2). To a solution of aniline **24-1** (6.4 g, 68.8 mmol) and con.HCl (11.6 mL, 36%, 139.2 mmol) in water (73 mL) was added a solution of $NaNO_2$ (4.8 g, 69.6 mmol) in water (12 mL) at 0 °C. After stirring 20 min, a solution of NaN_3 (4.5 g ,69 mmol) in water (20 mL) was added and the mixture was stirred at room temperature for 2 h. The reaction mixture was extracted with EtOAc (150 mL x 2). The combined extracts were washed with brine (100 mL x 3), dried over anhydrous Na_2SO_4 and concentrated in vacuum to give a white solid (5.4 g, crude) which was used directly in the next step.

Ethyl 5-methyl-1-phenyl-1H-1,2,3-triazole-4-carboxylate (24-3). To a solution of azidobenzene **24-2** (5.4 g, 45.4 mmol) in DMSO (8 mL) was added ethyl-3-oxobttamote (4.0 g, 30.8 mmol), diethylamine (219.0 mg, 3.0 mmol). The mixture was heated to 70 °C for 5 hr. The mixture was diluted with water (100 mL) and extracted with EtOAc (100 mL x 3). The combined extracts were washed with brine (100 mL x 3), dried over anhydrous Na_2SO_4 and concentrated in vacuum to give a yellow oil (3.1 g, 44%) which

was used directly in the next step. LC-MS (ESI) m/z = 232.1 $[M + 1]^+$, t = 0.897 min.

5-Methyl-1-phenyl-1H-1,2,3-triazole-4-carboxylic acid (24-4). To a solution of ethyl 5-methyl-1-phenyl-1H-1,2,3-triazole-4-carboxylate **24-3** (1.5 g, 6.5 mmol) in MeOH (20 mL) was added NaOH aqueous (6.4 mL, 2M, 13.8 mmol). The mixture was stirred at room temperature for 2 hr. The solvent was evaporated and diluted with water (10 mL). The aqueous layer was washed with EtOAc (50 mL x 2). The aqueous phase was acidified with 2 M HCl and extracted with EtOAc (30 mL x 2). The combined organic extracts were washed with brine (50 mL), dried with anhydrous Na_2SO_4, filtered and concentrated in vacuum to yield a yellow solid (880.0 mg, 68%) which used directly in the next step. LC-MS (ESI) m/z = 204.1 $[M + 1]^+$, t = 0.754 min.

***N*-(1-hydroxy-1,3-dihydrobenzo[c][1,2]oxaborol-6-yl)-1-(6-methoxypyridin-2-yl)-5-methyl-1H-1,2,3-triazole-4-carboxamide (25)**

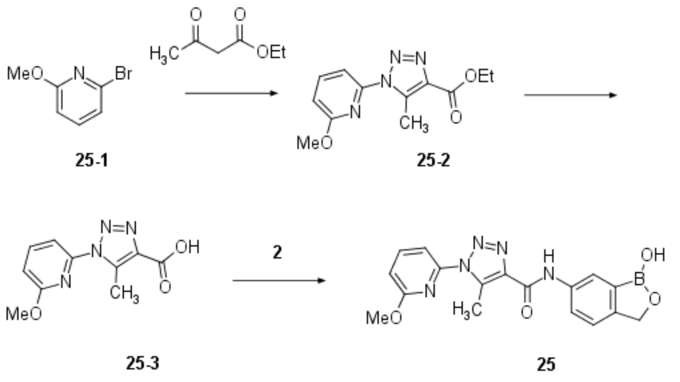

Ethyl 1-(6-methoxypyridin-2-yl)-5-methyl-1H-1,2,3-triazole-4-carboxylate (25-2). To a suspension of 2-bromo-6-methoxypyridine **25-1** (1.0 g, 5.3 mmol), ethyl 3-

oxobutanoate (689.0 mg, 5.3 mmol), L-proline (127.0 mg, 1.1 mmol), Na_2CO_3 (117.0 mg, 1.1 mmol), Na-ascrobate (105.0 mg, 0.5 mmol) and copper sulfate pentahydrate (66.0 mg, 0.3mmol) in DMSO (12 mL) and H_2O (1.4 mL) was added NaN_3 (416.0 mg, 6.4 mmol). The reaction was stirred at 100 °C overnight. After cooling to room temperature, water (30 mL) was added and the reaction mixture was extracted with EtOAc (30 mL x 2). The combined organic extracts were washed with brine (50 mL x 2), dried with anhydrous Na_2SO_4, filtered and concentrated in vacuum to give a yellow solid (814.0 mg, crude) which was used directly in the next step.

1-(6-Methoxypyridin-2-yl)-5-methyl-1H-1,2,3-triazole-4-carboxylic acid (25-3). To a solution of ethyl 1-(6-methoxypyridin-2-yl)-5-methyl-1H-1,2,3-triazole -4-carboxylate **25-2** (814.0 mg, 3.1 mmol) in THF (10 mL) was added NaOH aqueous (6.2 mL, 2 M, 12.4 mmol). The reaction solution was heated at refluxing overnight. The solvent was evaporated and diluted with water (5 mL). The aqueous layer was washed with DCM (5 mL). The aqueous phase was acidified with 3 M HCl, and extracted with EtOAc (30 mL x 2). The combined organic extracts were washed with brine (50 mL), dried with anhydrous Na_2SO_4, filtered and concentrated in vacuum to give a white solid (450.0 mg, crude) which was used directly in the next step.

***N*-(1-hydroxy-1,3-dihydrobenzo[*c*][1,2]oxaborol-6-yl)-5-methyl-1-(6-methylpyridin-2-yl)-1*H*-1,2,3-triazole-4-carboxamide (26)**

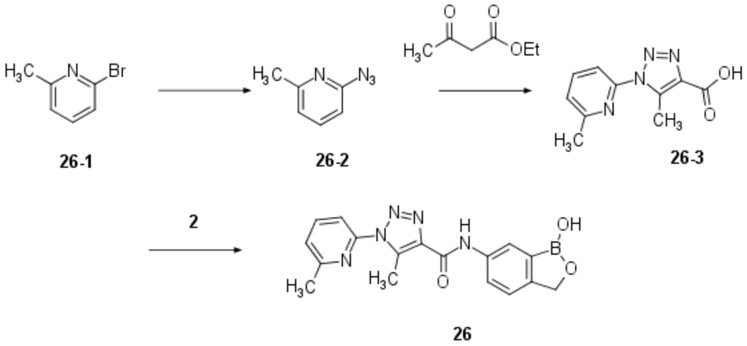

2-Azido-6-methylpyridine (26-2). To a solution of 2-bromo-6-methylpyridine **26-1** (5.0 g, 29.4 mmol) in DMSO (50 mL) was added NaN_3 (2.3 g, 35.4 mmol) and stirred at 120 °C overnight. After cooling to room temperature, $NaHCO_3$ aqueous (80 mL, 5%) was added and the mixture was extracted with EtOAc (100 mL x 2). The combined organic extracts were dried over anhydrous Na_2SO_4, concentrated in vacuum and purified silica gel chromatography (elution with Petroleum ether/Ethyl acetate = 1 : 1) to yield a white solid (1.5 g, 38%).

5-Methyl-1-(6-methylpyridin-2-yl)-1H-1,2,3-triazole-4-carboxylic acid (26-3). To a solution of 2-azido-6-methylpyridine **26-2** (300.0 mg, 2.2 mmol) in EtOH (5 mL) was added ethyl 3-oxobutanoate (286.0 mg, 2.2 mmol) and NaOEt (177.0 mg, 2.6 mmol). The mixture was stirred at refluxing overnight. After cooling to room temperature, water (2 mL) was added and the mixture was stirred for 2 h. The solvent was evaporated and diluted with water (3 mL). The aqueous layer was washed with EtOAc (5 mL). The aqueous phase was acidified with 3 M HCl, and extracted with EtOAc (5 mL x 2). The combined organic extracts were washed with brine (10 mL), dried with anhydrous Na_2SO_4, filtered and concentrated in vacuum to give a yellow solid (80.0 mg, crude) which was used directly in the next step.

_N_-(1-hydroxy-1,3-dihydrobenzo[c][1,2]oxaborol-6-yl)-5-methyl-1-(pyridin-3-yl)-1H-1,2,3-triazole-4-carboxamide (27)

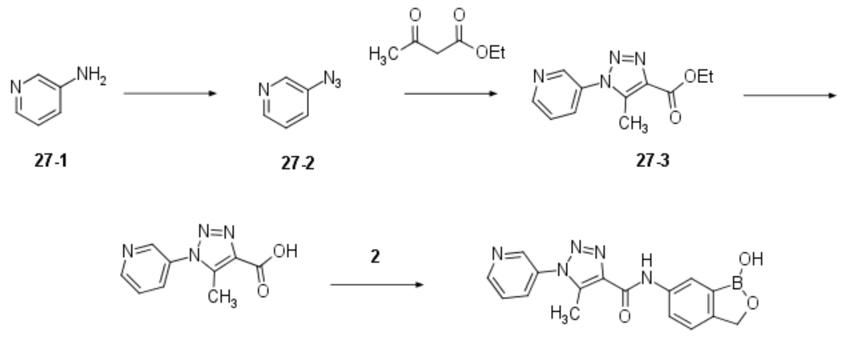

3-Azidopyridine (27-2). Compound **27-2** was synthesized according to the same procedure as preparing of **24-2** using pyridin-3-amine **27-1** (10.0 g, 106.4 mmol), sodium nitrite (8.8 g, 127.5 mmol), sodium azide (6.8 g, 104.6 mmol) and water (40 mL) to yield a red oil (11.0 g, crude) which was used directly in the next step.

Ethyl 5-methyl-1-(pyridin-3-yl)-1H-1,2,3-triazole-4-carboxylate (27-3). Compound **27-3** was synthesized according to the same procedure as preparing of **24-3** using 3-azidopyridine **27-2** (1.0 g, 8.3 mmol), ethyl 3-oxobutanoate (1.1 g, 8.5 mmol), Et_2NH (62.0 mg, 0.8 mmol) and DMSO (5 mL) to yield a yellow solid (1.7 g, crude) which was used directly in the next step. LC-MS (ESI) m/z = 232.1 $[M + 1]^+$, t = 0.467 min.

5-Methyl-1-(pyridin-3-yl)-1H-1,2,3-triazole-4-carboxylic acid (27-4). Compound **27-4** was synthesized according to the same procedure as preparing of **24-4** using ethyl 5-methyl-1-(pyridin-3-yl)-1H-1,2,3-triazole-4-carboxylate **27-3** (1.7 g, 7.3 mmol),

NaOH (8 mL, 2 M, 16.0 mmol) and MeOH (10 mL) to yield a yellow solid (1.3 g, crude) which was used directly in the next step.

_N_-(1-hydroxy-1,3-dihydrobenzo[c][1,2]oxaborol-6-yl)-3-methyl-4-(pyridin-2-yl)furan-2-carboxamide (28)

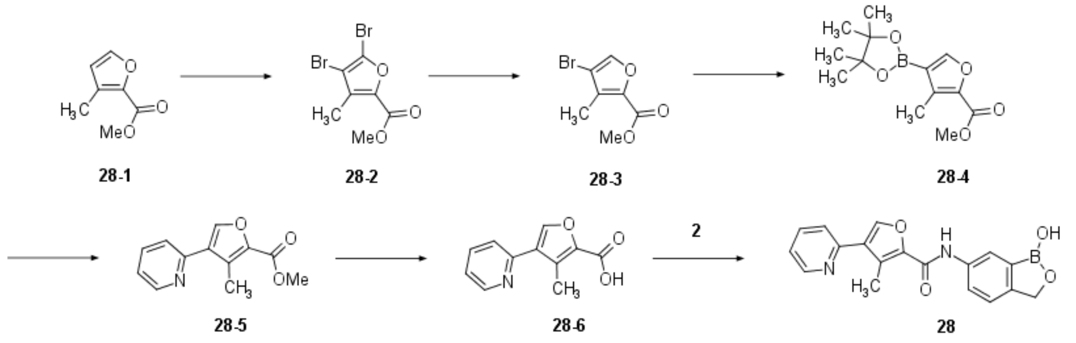

4, 5-Dibromo-3-methyl-furan-2-carboxylic acid methyl ester (28-2). 3-methyl-furan-2-carboxylic acid methyl ester **28-1** (40.0 g, 285.7 mmol) was added to a cooled mixture of $AlCl_3$ (70.0 g, 530.3 mmol) in DCM (250 mL) at 0 °C -10 °C. A solution of Br_2 (29 mL, 572.0 mmol) in DCM (50 mL) was added at 0 °C -10 °C. The mixture was stirred at 0 °C -10 °C for 30 min and then stirred at room temperature for 2 h. Water (600 mL) was added slowly at room temperature. The organic layer was separated and the aqueous layer was extracted with DCM (200 mL). The combined organic extracts was washed with saturated solution Na_2SO_3 solution (400 mL x 2), dried with Na_2SO_4 and concentrated in vacuum to yield a light yellow solid (77.6 g, 92 %). ^{1}H NMR (400MHz, DMSO-d_6) δ 3.82 (s, 3H), 2.27 (s, 3H).

4-Bromo-3-methyl-furan-2-carboxylic acid methyl ester (28-3). To a mixture of 4,5-dibromo-3-methyl-furan-2-carboxylic acid methyl ester **28-2** (77.6 g, 262.1 mmol) and NH_4Cl (139.0 g, 2.6 mol) in MeOH (400 mL) was added Zinc dust (167.0 g, 2.6 mol) at room temperature. The mixture was stirred at 0 °C -10 °C for 2 h and filtered through a celite pad and the filtrate was concentrated in vacuum. Water (400 mL) was added and the reaction mixture was extracted with DCM (300 mL). The combined organic extracts were washed with brine (300 mL), dried with anhydrous Na_2SO_4, filtered and concentrated in vacuum to give a buff solid (52.2 g, 91%). ^1H NMR (400MHz, DMSO-d_6) δ 8.14 (s, 1H), 3.81 (s, 3H), 2.23 (s, 3H).

Methyl 3-methyl-4-(4,4,5,5-tetramethyl-1,3,2-dioxaborolan-2-yl)furan-2-carboxylate (28-4). To a solution of 4-bromo-3-methyl-furan-2-carboxylic acid methyl ester **28-3** (10.0 g, 45.9 mmol) was added 4,4,4',4',5,5,5',5'-octamethyl-2,2'-bi(1,3,2-dioxaborolane) (17.5 g, 68.9 mmol), AcOK (6.7 g, 68.9 mmol) and Pd(dppf)Cl_2 (2.7 g, 3.7 mmol) in dioxane (100 mL). The mixture was stirred at 85 °C overnight. The mixture was filtered through a celite pad and the filtrate was concentrated in vacuum to give a residue. Water (400 mL) was added to the residue and the mixture was extracted with EtOAc (400 mL). The organic layer was dried with anhydrous Na_2SO_4, concentrated in vacuum and purified by flash to yield a white solid (7.7 g, 63 %). ^1H NMR (400MHz, $CDCl_3$) δ = 7.76 (s, 1H), 3.90 (s, 3H), 2.47 (s, 3H), 1.32 (s, 12H).

Methyl 3-methyl-4-(pyridin-2-yl)furan-2-carboxylate (28-5). To a solution of methyl 3-methyl-4-(4,4,5,5-tetramethyl-1,3,2-dioxaborolan-2-yl)furan-2-carboxylate

28-4 (4.5 g, 16.9 mmol), K_2CO_3 (3.12 g, 22.6 mmol), PPh_3 (1.2 g, 4.5 mmol) and 2-chloropyridine (1.3 g, 11.3 mmol) in toluene (45 mL) and MeOH (45 mL) was added and $Pd(OAc)_2$ (253.0 mg, 1.1 mmol). The mixture was stirred at 65 ºC overnight under nitrogen atmosphere. After cooling to room temperature, the mixture was filtered through a celite pad and the filtrate was concentrated in vacuum. Water (150 mL) was added and the mixture was extracted with EtOAc (150 mL). The organic extract was washed with brine (100 mL), dried with anhydrous Na_2SO_4, filtered and concentrated in vacuum to yield a white solid (2.6 g, crude). ^{1}H NMR (400MHz, DMSO-d_6) δ 8.63 (dd, *J* = 5.2, 1.6 Hz, 1H), 8.40 (s, 1H), 7.86 (m, 1H), 7.68 (d, *J* = 8.0 Hz, 1H), 7.34 (dd, *J* = 6.0, 1.2 Hz, 1H), 3.83 (s, 3H), 2.57 (s, 3H).

3-Methyl-4-(pyridin-2-yl)furan-2-carboxylic acid (28-6). To a solution of methyl 3-methyl-4-(pyridin-2-yl)furan-2-carboxylate **28-5** (2.6 g, 12.1 mmol) in MeOH (5 mL) was added LiOH aqueous (12 mL, 2 M, 24.2 mmol). The mixture was stirred at room temperature overnight. The solvent was evaporated and diluted with water (150 mL). The mixture was washed with EtOAc (150 mL). The aqueous phase was acidified with 2 M HCl, and extracted with EtOAc (50 mL x 5). The combined organic extracts were dried with anhydrous Na_2SO_4, filtered and concentrated in vacuum to yield a white solid (1.70 g, 69 %). ^{1}H NMR (400MHz, DMSO-d_6) δ 8.63 (dd, *J* = 4.8, 0.8 Hz, 1H), 8.33 (s, 1H), 7.85 (dd, *J* = 7.6, 6.0 Hz, 1H), 7.66 (d, *J* = 8.0 Hz, 1H), 7.33 (m, 1H), 2.56 (s, 3H).

***N*-(1-hydroxy-1,3-dihydrobenzo[c][1,2]oxaborol-6-yl)-5-methyl-4-(pyridin-2-**

yl)furan-2-carboxamide (29)

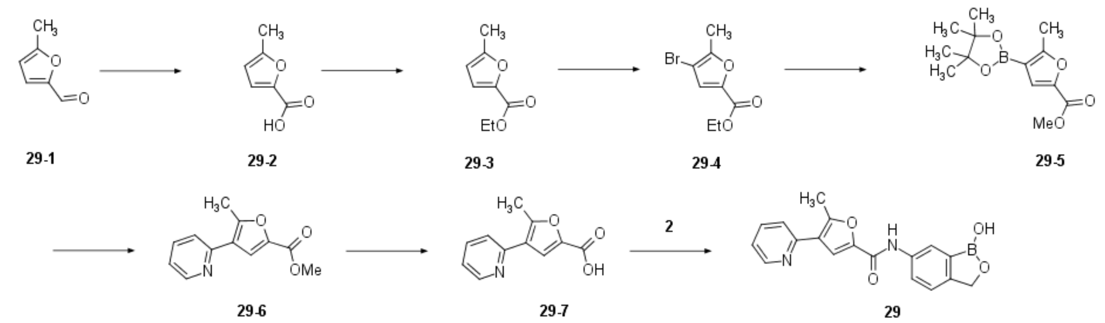

5-Methylfuran-2-carboxylic acid (29-2). To a solution of 5-methylfuran-2-carbaldehyde **29-1** (130.0 g, 1.2 mol) in acetone (600 mL) was added a solution of sulfamic acid (228.9 g, 2.4 mol) and H_2O (100 mL) at 0 °C -10 °C and the mixture was stirred at 0 °C -10 °C for 5 min. Then $NaClO_2$ (138.0 g, 1.6 mol) was added in portions at 0 °C -10 °C and the mixture was stirred at 0 °C -10 °C for 30 min. The pH was adjusted to 9 with 2 M NaOH aqueous, and the mixture was washed with EtOAc (500 mL x 2). The aqueous phase was adjusted pH to 2, extracted with EtOAc (500 mL x 3). The combined extracts were dried over anhydrous Na_2SO_4 and concentrated in vacuum to give a yellow solid (80.0 g, crude) which was used directly in the next step.

Ethyl 5-methylfuran-2-carboxylate (29-3). To a solution of 5-methylfuran-2-carboxylic acid **29-2** (80.0 g, crude) in EtOH (600 mL) was added $SOCl_2$ (30 mL). The reaction mixture was stirred at refluxing overnight. After cooling to room temperature, the mixture was concentrated in vacuum to give a residue. The residue was diluted with water (150 mL) and extracted with EtOAc (100 mL x 2). The combined organic extracts were washed with brine (100 mL), dried over anhydrous Na_2SO_4 and concentrated in vacuum to yield a white solid (120.0 g, crude).

Ethyl 4-bromo-5-methylfuran-2-carboxylate (29-4). To a solution of ethyl 5-methylfuran-2-carboxylate **29-3** (107.0 g, 695.0 mmol) in DCM (700 mL) was added $AlCl_3$ (184.0 g, 1.4 mol) at 0 ºC. After stirring for 10 min, Br_2 (36 mL, 695 mmol) was added at 0 ºC. The mixture was stirred at room temperature for 1.5 h. The mixture poured into ice-water (2 L) and extracted with DCM (1 L). The organic phase was washed H_2O (1 L), dried over anhydrous Na_2SO_4 and concentrated in vacuum to give a yellow oil (106.0 g, crude) which was used directly in the next step.

Ethyl 5-methyl-4-(4,4,5,5-tetramethyl-1,3,2-dioxaborolan-2-yl)furan-2-carboxylate (29-5). To a solution of ethyl 4-bromo-5-methylfuran-2-carboxylate **29-4** (40.0 g, 173.2 mmol), 4,4,4',4',5,5,5',5'-octamethyl-2,2'-bi(1,3,2-dioxaborolane) (56.0 g, 220.4 mmol) and KOAc (33.5 g, 341.8 mmol) in dioxane (400 mL) was added Pd(dppf)Cl_2 (12.5 g, 17.1 mmol). The mixture was stirred at 85 ºC overnight under N_2. The reaction was concentrated in vacuum and purified by silica gel chromatography to give a yellow solid (30 g, crude) which was used directly in the next step.

Ethyl 5-methyl-4-(pyridin-2-yl)furan-2-carboxylate (29-6). To the solution of ethyl 5-methyl-4-(4,4,5,5-tetramethyl-1,3,2-dioxaborolan-2-yl)furan-2-carboxylate **29-5** (22.0 g, 78.6 mmol), 2-bromopyridinem (14.8 g, 94.3 mmol) and KOAc (15.4 g, 157.2 mmol) in dioxane (200 mL) and H_2O (50 mL) was added Pd(dppf)Cl_2 (5.7 g, 7.8 mmol). The mixture was stirred at 85 ºC overnight under N_2. After cooling to room temperature, water (500 mL) was added and the mixture was extracted with EtOAc (300 mL x 2).

The combined organic extracts were dried over anhydrous Na_2SO_4 and concentrated in vacuum and purified by silica gel chromatography to give product (7.5 g, 41.6%).

5-Methyl-4-(pyridin-2-yl)furan-2-carboxylic acid (29-7). To a solution of ethyl 5-methyl-4-(pyridin-2-yl)furan-2-carboxylate **29-6** (13.0 g, 56.2 mmol) in MeOH (60 mL) was added NaOH aqueous (56 mL, 2 M, 112 mmol). The mixture was stirred at room temperature overnight. The solvent was evaporated and diluted with water (500 mL). The mixture was washed with DCM (300 mL). The aqueous phase was acidified with 2 M HCl, and extracted with EtOAc (50 mL x 5). The combined organic extracts were dried with anhydrous Na_2SO_4, filtered and concentrated in vacuum to yield a white solid (11 g, 96%).

***N*-(1-hydroxy-1,3-dihydrobenzo[c][1,2]oxaborol-6-yl)-3-methyl-4-(pyridin-2-yl)thiophene-2-carboxamide (30)**

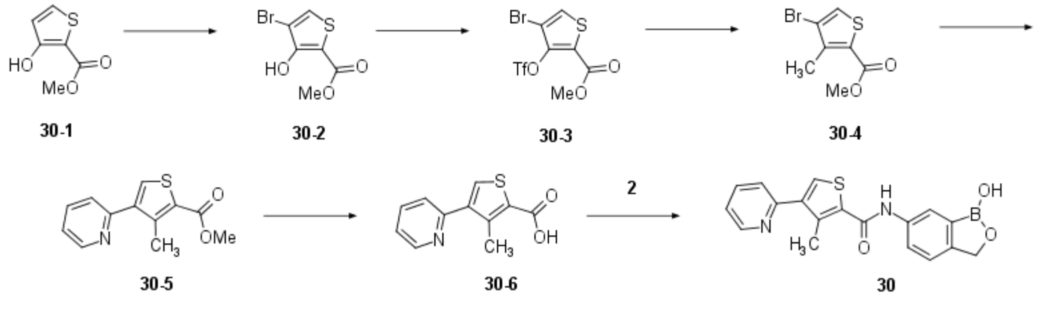

Methyl 4-bromo-3-hydroxythiophene-2-carboxylate (30-2). To a solution of methyl 3-hydroxythiophene-2-carboxylate **30-1** (4.5 g, 28.5 mmol) in AcOH (45 mL) at 0 °C - 10 °C a solution of Br_2 (1.5 mL) in AcOH (13 mL) was added. After stirring at 0 °C -

10 °C for 15 min, the mixture warmed to room temperature and stirred for 2 h. Water (200 mL) was added and a lot of solid formed. The precipitate was filtered and the cake was washed with PE (20 mL), dried in vacuum to give a white solid (2.1 g, crude) which used directly in the next step. ^{1}H NMR (400MHz, DMSO-d_6) δ = 9.67 (s, 1H), 7.31 (s, 1H), 3.85 (s, 3H).

Methyl 4-bromo-3-(((trifluoromethyl)sulfonyl)oxy)thiophene-2-carboxylate (30-3). To a solution of 5- methyl 4-bromo-3-hydroxythiophene-2-carboxylate **30-2** (2.1 g, 8.9 mmol), Et_3N (1.1 g, 10.8 mmol) and DMAP (50 mg, 0.4 mmol) in DCM (20 mL) was added Tf_2O (2.5 g, 8.9 mmol) at 0 °C -10 °C. The mixture was stirred at room temperature for 2 h. The reaction mixture was washed with saturated $NaHCO_3$ solution (30 mL x 2). The organic layer was dried over anhydrous Na_2SO_4, concentrated in vacuum and purified by silica gel chromatography to give crude product (2.9 g, crude) which was used directly in the next step. ^{1}H NMR (400MHz, DMSO-d_6) δ = 7.48 (s, 1H), 3.88 (s, 3H).

Methyl 4-bromo-3-methylthiophene-2-carboxylate (30-4). To a mixture of methyl 4-bromo-3-(((trifluoromethyl)sulfonyl)oxy)thiophene-2-carboxylate **30-3** (1.5 g, 4.1 mmol), K_2CO_3 (1.7 g, 12.3 mmol) and $CH_3B(OH)_2$ (268.0 mg, 4.1 mmol) in dioxane (7.5 mL) and H_2O (1.5 mL) were added $Pd(PPh_3)_4$ (480.0 mg, 0.4 mmol). The mixture was heated at 70 °C overnight in a sealed tube. After cooling to room temperature, the mixture was diluted with MeOH (15 mL) and filtered through celite pad. The filtrate was concentrated in vacuum and purified by silica gel chromatography to give the

product (500.0 mg, 52%). ^{1}H NMR (400MHz, DMSO-d_6) δ 7.36 (s, 1H), 3.85 (s, 3H), 2.48 (s, 3H).

Methyl 3-methyl-4-(pyridin-2-yl)thiophene-2-carboxylate (30-5) To a mixture of methyl 4-bromo-3-methylthiophene-2-carboxylate **30-4** (300.0 mg, 1.3 mmol), CsF (650 mg, 4.3 mmol) and 2-(tributylstannyl)pyridine (0.7 g, 1.9 mmol) was added $Pd(PPh_3)_4$ (130.0 mg, 0.1 mmol). The mixture was stirred at 130 °C for 45 min under microwave. After cooling to room temperature, water (40 mL) was added and the mixture was extracted with EtOAc (20 mL x 2). The combined organic extracts were concentrated in vacuum and purified by silica gel chromatography to yield the product (150.0 mg, 50%). ^{1}H NMR (400MHz, DMSO-d_6) δ 8.62 (m, 2H), 8.33 (m, 2H), 7.61 (s, 1H), 3.85 (s, 3H), 2.59 (s, 3H).

3-Methyl-4-(pyridin-2-yl)thiophene-2-carboxylic acid (30-6) To a solution of methyl 3-methyl-4-(pyridin-2-yl)thiophene-2-carboxylate **30-5** (150.0 mg, 0.6 mmol) in MeOH (5 mL) was added LiOH (2 mL, 1.5 M, 3 mmol). The mixture was stirred at room temperature overnight. The mixture was concentrated in vacuum to remove MeOH, and then diluted with water (10 mL). The pH was adjusted to 4 and the mixture was extracted with DCM (10 mL x 2). The combined organic extracts were washed with brine (20 mL), dried over anhydrous Na_2SO_4, concentrated in vacuum to give the product (100.0 mg, 71%). ^{1}H NMR (400 MHz, DMSO-d_6): δ 8.66 (d, J=4.4 Hz, 1H), 8.04 (s, 1H), 7.89 (m, 1H), 7.62 (d, J=7.6 Hz, 1H), 7.38 (m, 1H), 2.62 (s, 3H).

***N*-(1-hydroxy-1,3-dihydrobenzo[c][1,2]oxaborol-6-yl)-5-(pyridin-2-yl)-1,3,4-oxadiazole-2-carboxamide (31)**

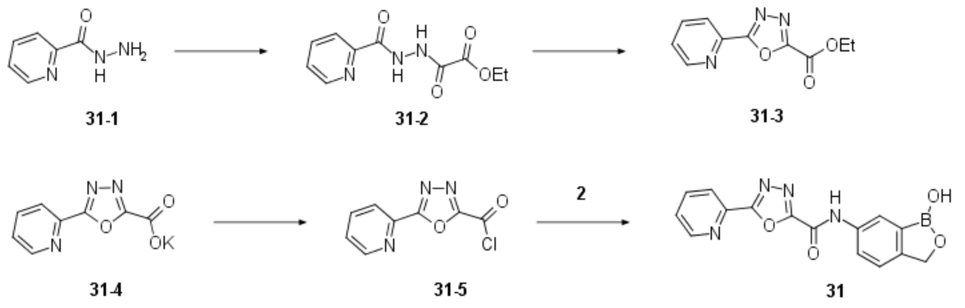

Ethyl 2-oxo-2-(2-picolinoylhydrazinyl)acetate (31-2). To a solution of pyridine-2-carbohydrazide **31-1** (10.0 g, 72.9 mmol) and TEA (22.14 g, 218.8 mmol) in DCM (250 mL) was added ethyl 2-chloro-2-oxo-acetate (11.9 g, 87.5 mmol) at 0 °C. The mixture was stirred at room temperature for 4 h. The solution of reaction was used into the next step directly.

Ethyl 5-(pyridin-2-yl)-1,3,4-oxadiazole-2-carboxylate (31-3). To a solution of ethyl 2-oxo-2-(2-picolinoylhydrazinyl)acetate **31-2** (17.3 g, 72.9 mmol) was added TEA (7.4 g, 72.9 mmol) and *p*-TosCl (9.6 g, 80.2 mmol) at room temperature for 3 h. The mixture was stirred at room temperature overnight. Water (400 mL) was added. The phase was separated and the aqueous phase was extracted with DCM (150mL x 2). The combined organic extracts were dried over anhydrous Na_2SO_4, concentrated in vacuum and purified silica gel chromatography (elution with Petroleum ether/Ethyl acetate= 6 : 1 to 1 : 1) to yield a white solid (11.2 g, 70%). ^{1}H NMR (400MHz, DMSO-d6) δ 8.84 (d, *J*= 4.8 Hz 1H), 8.30 (d, *J*= 8.0 Hz 1H), 8.13 (m, 1H), 7.73 (m, 1H), 4.50 (q, *J*= 7.2 Hz 2H), 1.40 (t, *J*=6.8 Hz, 3H).

Potassium 5-(pyridin-2-yl)-1,3,4-oxadiazole-2-carboxylate (31-4). To a solution of ethyl 5-(2-pyridyl)-1,3,4-oxadiazole-2-carboxylate **31-3** (7.0 g, 31.9 mmol) in THF (70 mL) and EtOH (35 mL) was added a solution of KOH (1.8 g, 32.1mmol) in H_2O (7.00 mL) at 0 °C. The mixture was stirred at 0 °C for 1 h. A lot of white solid formed. The reaction mixture was filtered and the cake was washed with THF (20 mL) and lyophilized to give a white solid (7.0 g, 96%). ^{1}H NMR (400MHz, DMSO-d6) δ 8.78 (d, *J*=4.4 Hz 1H), 8.27 (d, *J*=7.6 Hz 1H), 8.09 (m, 1H), 7.65 (m, 1H).

5-(pyridin-2-yl)-1,3,4-oxadiazole-2-carbonyl chloride (31-5). To a suspension of [5-(2-pyridyl)-1,3,4-oxadiazole-2-carbonyl]oxy potassium **31-4** (3.0 g, 13.1 mmol) and Py (1.04 g, 13.1 mmol) in MeCN (100 mL) was added oxalyl dichloride (3.7 g, 28.8 mmol, 2.52 mL) dropwise at 0 °C and then DMF (95.67 mg, 1.3 mmol) was added. The suspension was stirred at 0 °C for 2 h. The solution of reaction was used directly in the next step.

SUPPLEMENTARY FIGURES AND TABLES

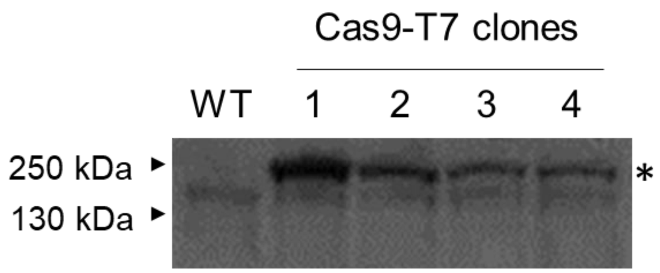

Figure S1. Validation of functional CRISPR-Cas9 system in *L. donovani*. Expression of Cas9 in pTB007-transfected cells was detected by western blotting, using anti-CRISPR-Cas9 [7A9-3A3] (1:5000, Abcam) and goat anti-mouse HRP (1:2000, Biorad) as primary and secondary antibodies, respectively. Cas9 (*, expected molecular weight ~165 kDa) was evident in all transgenic clones tested. Extracts from wild type parasites were used as negative control.

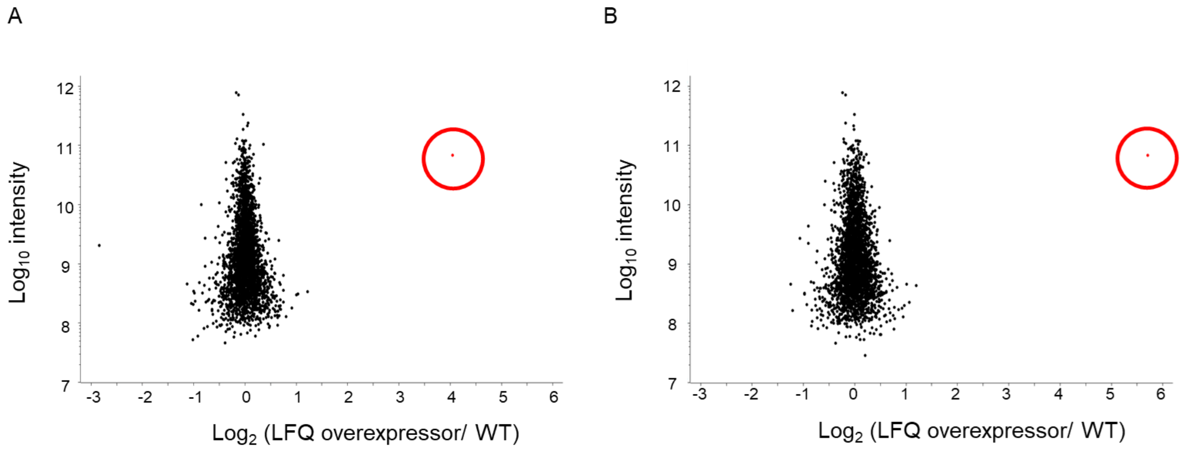

Figure S2. Label-free quantification (LFQ) of *L. donovani* promastigotes overexpressing CPSF3 (A) and CPSF3 (Asn^{219}His) (B). CPSF3 is highlighted and circled in red. Relative to endogenous levels of CPSF3 in wild-type promastigotes,

CPSF3 was 4-fold overexpressed in our transgenic cell line while CPSF3 (Asn[219]His) was 5.7-fold overexpressed.

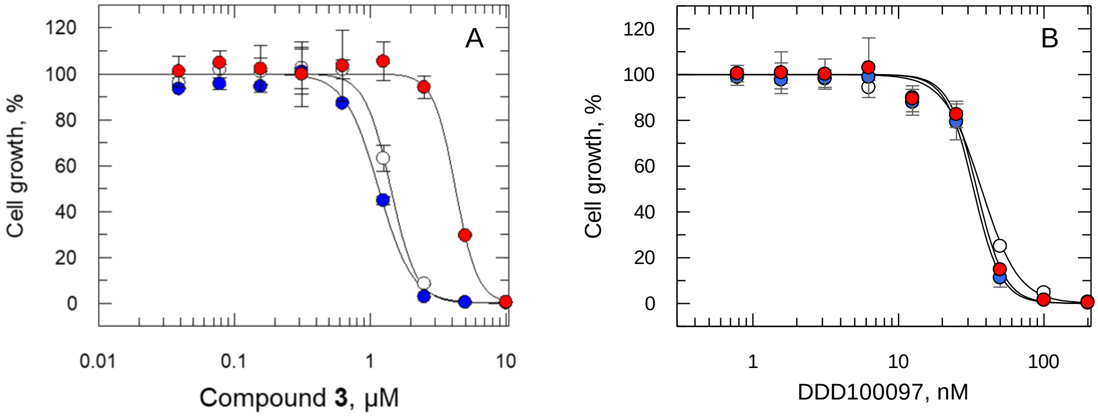

Figure S3. Potency of the compound 3 and the established *N*-myristoyltransferase-inhibitor DDD100097 against wild-type and transgenic *L. donovani*. (A) EC_{50} values of 1.2 ± 0.02, 1.4 ± 0.03 and 4.4 ± 0.2 µM were determined for SCYX-6759-treated wild-type (open circles), CPSF3-overexpressing (blue circles) and CPSF3 (Asn^{219}His)-overexpressing (red circles) *L. donovani* promastigotes, respectively. (B) EC_{50} values of 31 ± 1.2, 22 ± 0.5 and 19.4 ± 0.6 µM were determined for DDD100097-treated wild-type (open circles), CPSF3-overexpressing (blue circles) and CPSF3 (Asn^{219}His)-overexpressing (red circles) *L. donovani* promastigotes, respectively. All curves are the non-linear fits of data using a two-parameter EC_{50} equation provided by GraFit. EC_{50} values are the weighted mean ± standard deviation of at three biological replicates (n=3) with each biological replicate comprised of three technical replicates.

Figure S4. Activation of the benzoxaborole to boronate. The equilibrium between the neutral form of DNDI-6148 (a) and its boronate form (b).

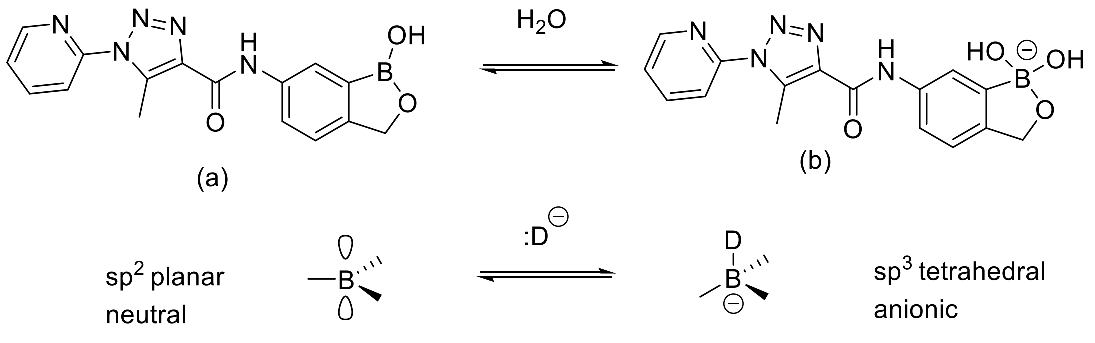

Figure S5. Sequence alignment used in the preparation of the *Ld*CPSF3 homology model. In pink the residues comprising the DNDI-6148 binding site. Residues Asn219 and Glu229 are highlighted in green.

```
L. donovani      VEVLPIGSGGEVGRSCVVVRYKGRGVMLDCGNHPAKSGLDSLPFFDSIKCDEIDVVLITH
T. thermophilus  MRIVPFGAAREVTGSAHLLLAGGRRVLLDCGMFQGKEEARNHAPFG~FDPKEVDAVLLTH

L. donovani      FHLDHCGALPYFCNQTSFKGRIFMTSATKAFYKMVMNDFLRIGAGASDLVTSEWLQSTID
T. thermophilus  AHLDHVGRLPKLFREG~YRGPVYATRATVLLMEIVLEDALKVMDEP~~FFGPEDVEEALG

L. donovani      RIETVEYHEEVTVNGISFQPFNAGHVLGAAMFMVDIAGMRALYTGDFSRVPDRHLLGAEV
T. thermophilus  HLRPLEYGEWLRLGALSLAFGQAGHLPGSAFVVAQGEGRTLVYSGDLGNREKDVLPDPSL

L. donovani      PPYSPDILIAESTNGIRELESREEREHLFTSSVHDVVRRGGRCLVPVFALGRAQELLLIL
T. thermophilus  PPLA~DLVLAEGTYGDRPHRPYRETVREFLEILEKTLSQGGKVLIPTFAVERAQEILYVL

L. donovani      EEFWDAHKELQNIPIYYASSLAQRCMKLYQTFVSAMNDRVKQQHANHHNPFVFKYIHSLM
T. thermophilus  YTHG~~~HRLPRAPIYLDSPMAGRVLSLYPRLVRYFSEEVQAHFLQGKNPFRPAGLEVVE

L. donovani      DTKSFEDN~~~~GPCVVLASPGMLQSGISLELFERWCGDRRNGIIMAGYCVDGTIAKDVL
T. thermophilus  HTEASKALNRAPGPMVVLAGSGMLAGGRILHHLKHGLSDPRNALVFVGYQPQGGLGAEII

L. donovani      AKPKEVAKPDGKVLPLRMSTIEAVSFSAHSDGRQTRDFIQSLTKVKHTILVHGNPGAMGQ
T. thermophilus  ARP~PAVRILGEEVPLRASVHTLGGFSGHAGQDELLDWLQ~~~GEPRVVLVHGEEEKLLA

L. donovani      LGKLLALRGQEVSLARFGEGVPV
T. thermophilus  LKSKLLQDFRDRNMSVYTTMNQE
```

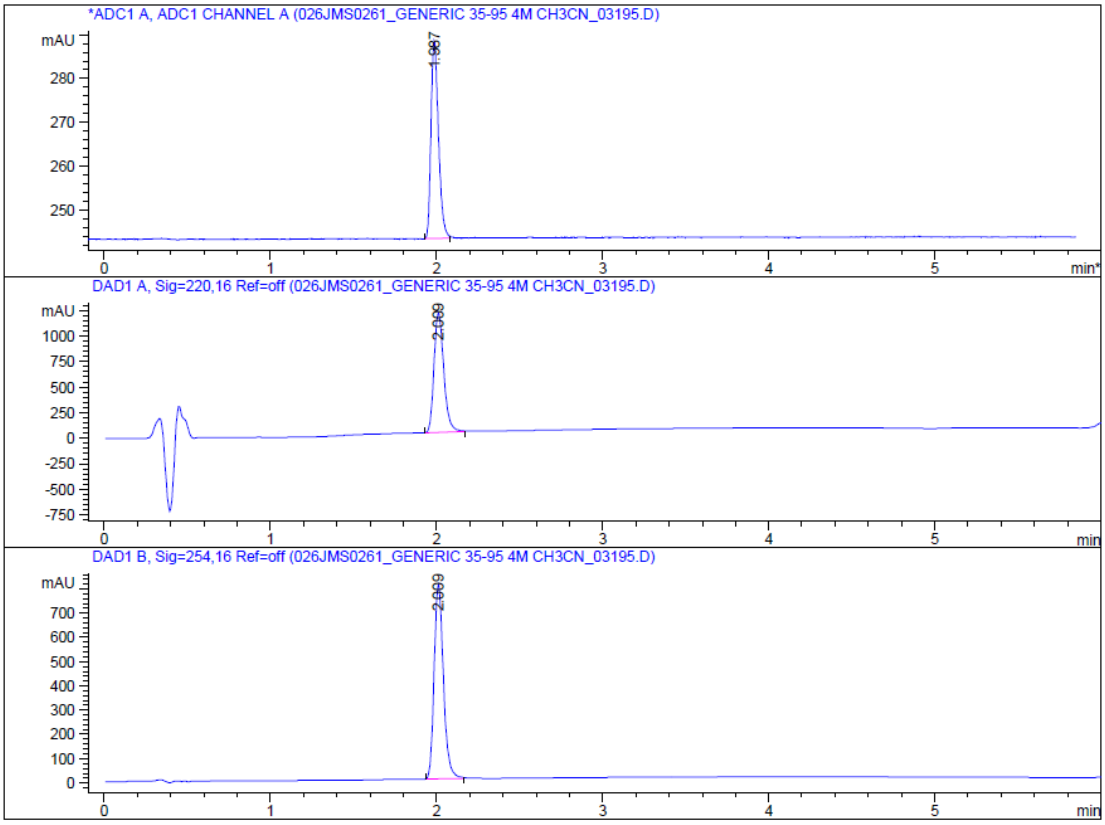

Figure S6: HPLC chromatogram of compound 3. HPLC conditions: 65:35→5:95 water (+0.1% formic acid (v/v)):MeCN (+0.1% formic acid (v/v)).

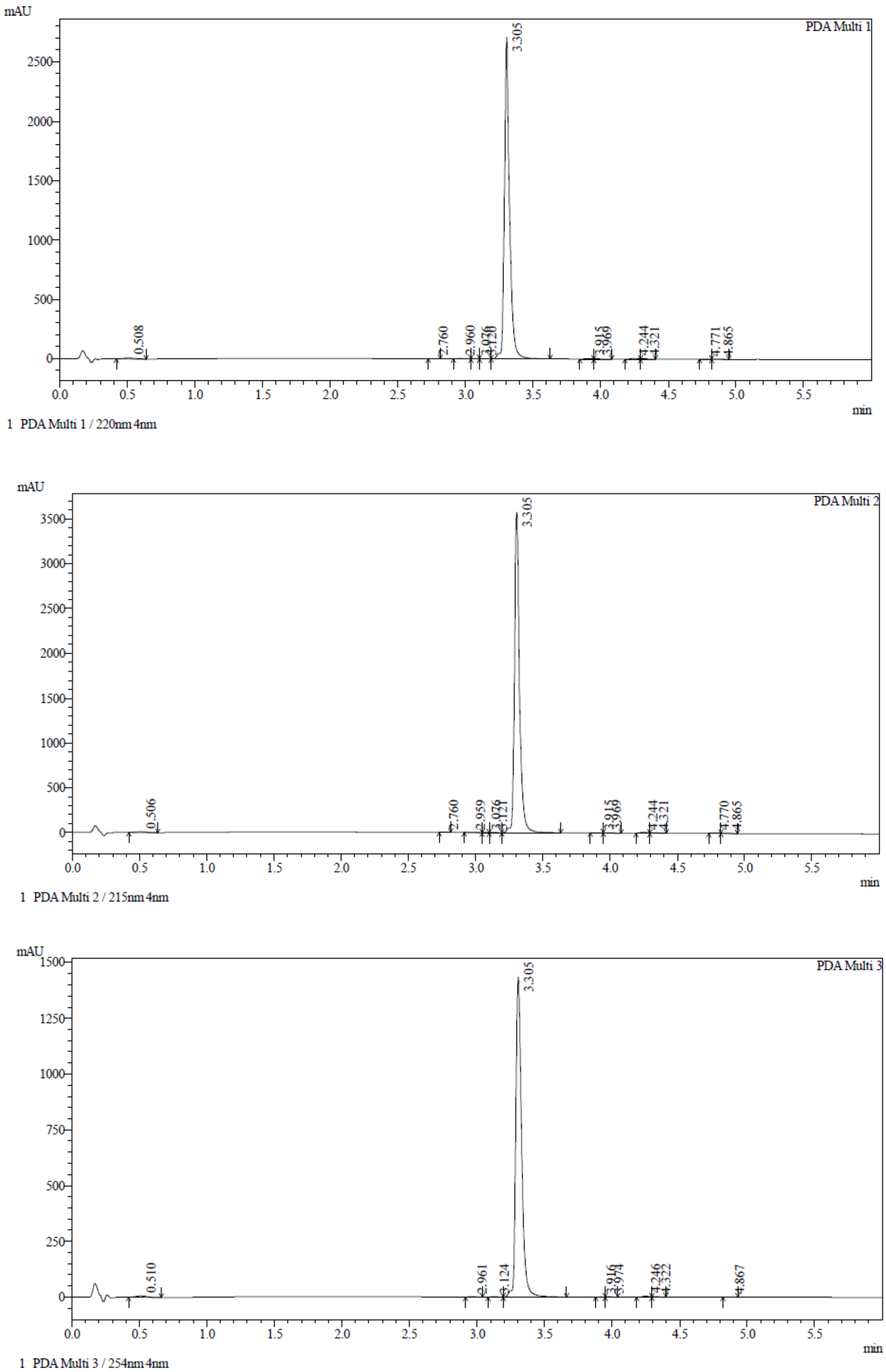

Figure S7: HPLC chromatogram of compound 5. HPLC conditions: 100:0→40:60 water (+0.0375% TFA (v/v)):MeCN (+0.01875% TFA (v/v)).

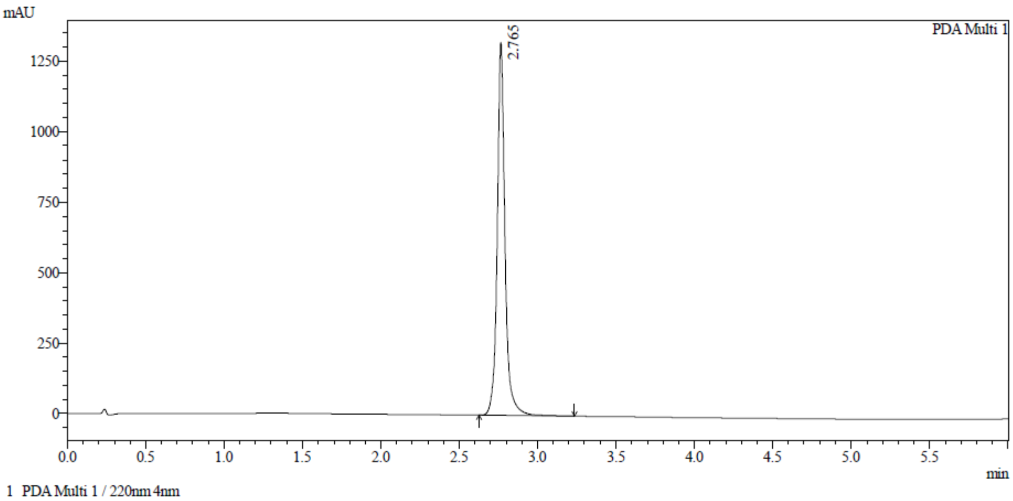

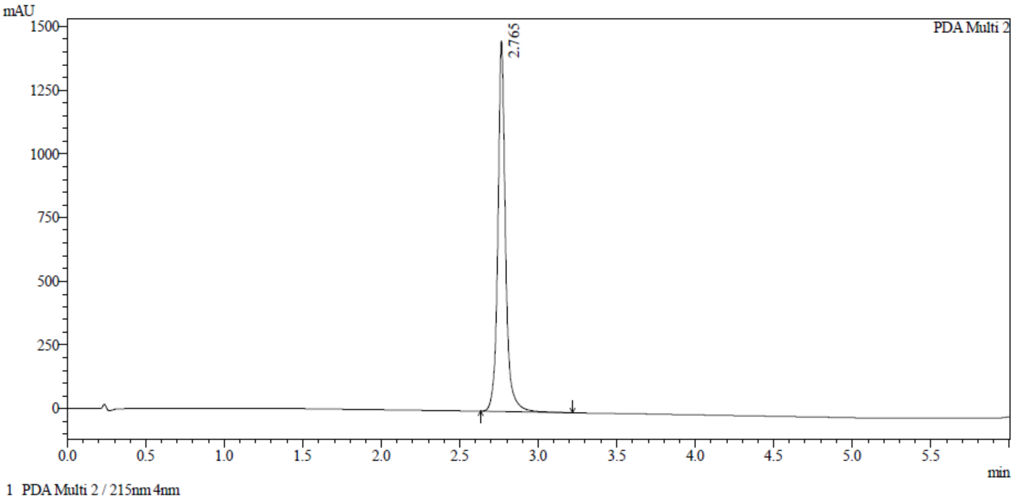

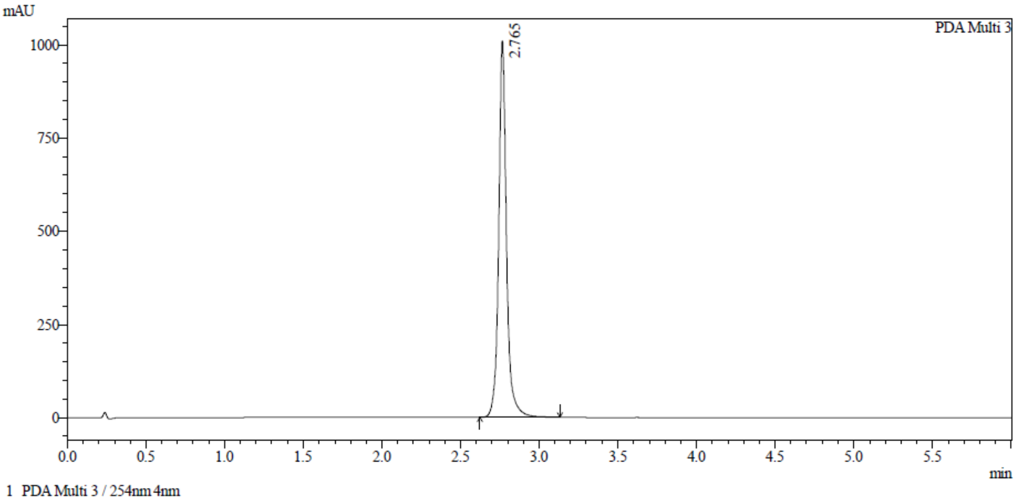

Figure S8: HPLC chromatogram of compound 16. HPLC conditions: 100:0→40:60 water (+0.0375% TFA (v/v)):MeCN (+0.01875% TFA (v/v)).

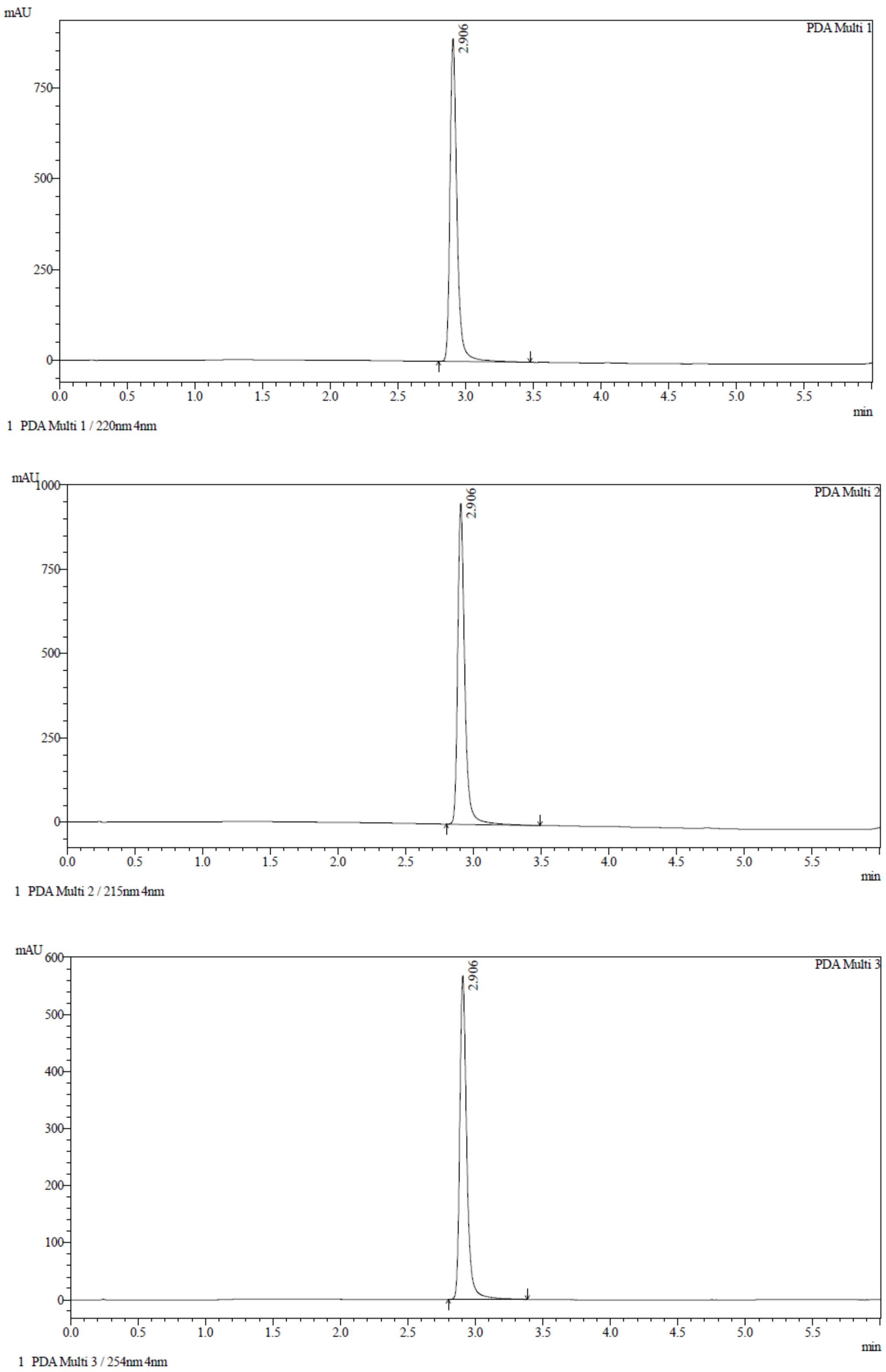

Figure S9: HPLC chromatogram of compound 23. HPLC conditions: 100:0→40:60 water (+0.0375% TFA (v/v)):MeCN (+0.01875% TFA (v/v)).

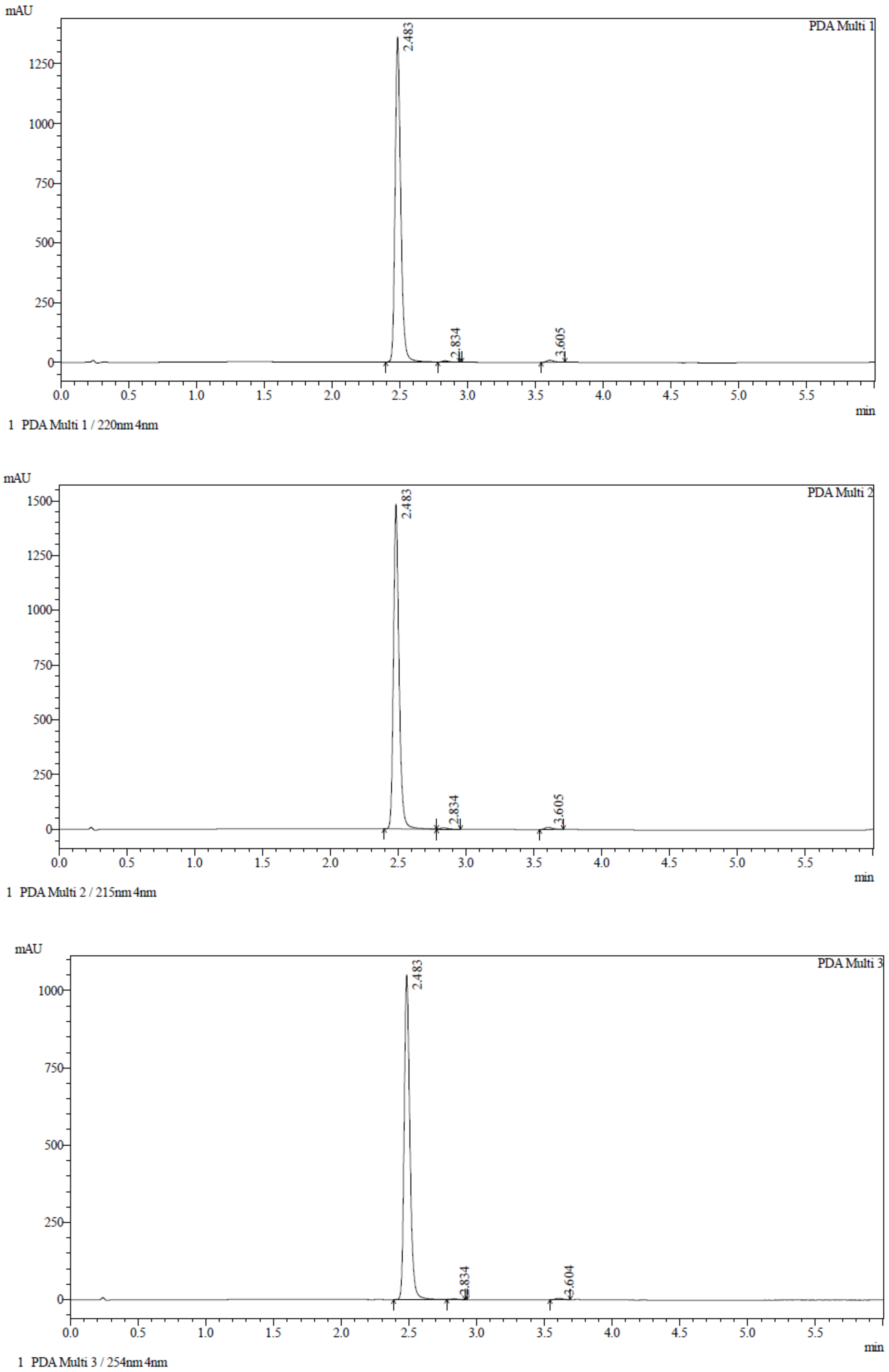

Figure S10: HPLC chromatogram of compound 28. HPLC conditions: 100:0→40:60 water (+0.0375% TFA (v/v)):MeCN (+0.01875% TFA (v/v)).

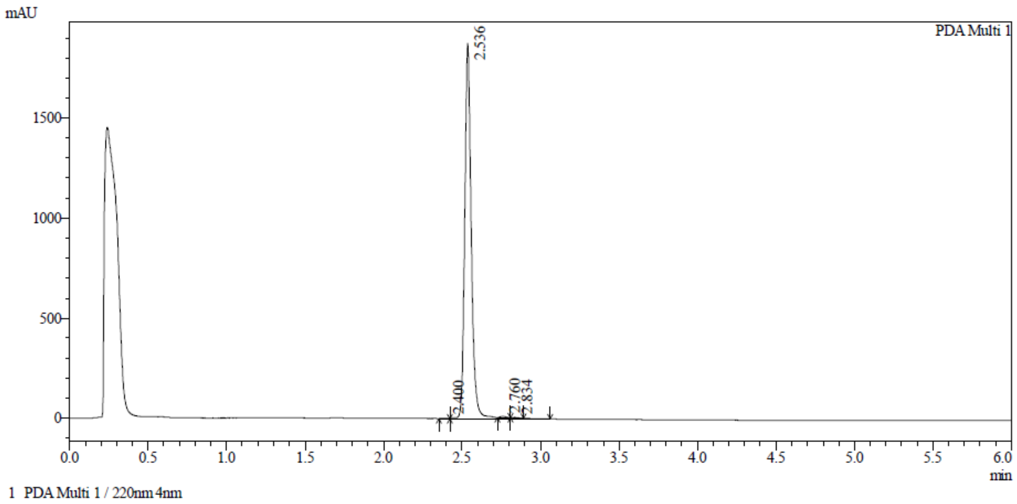

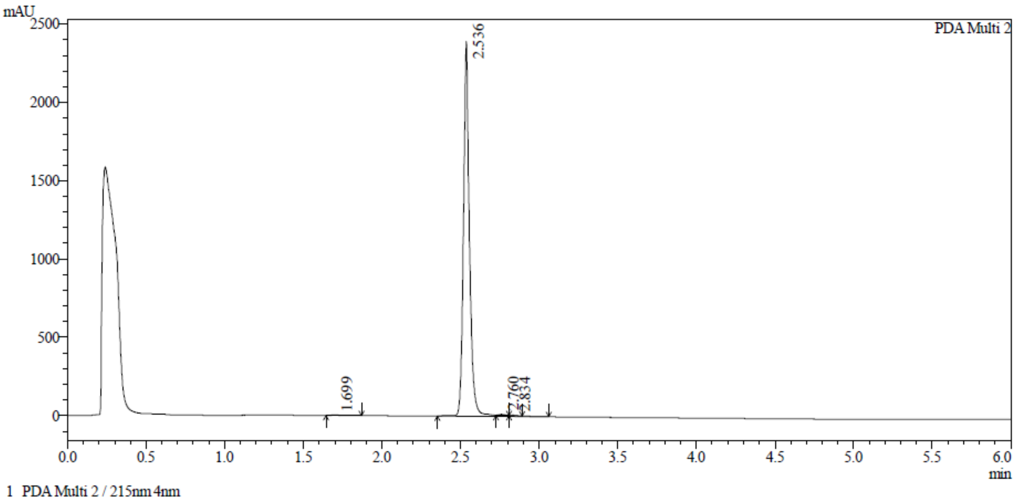

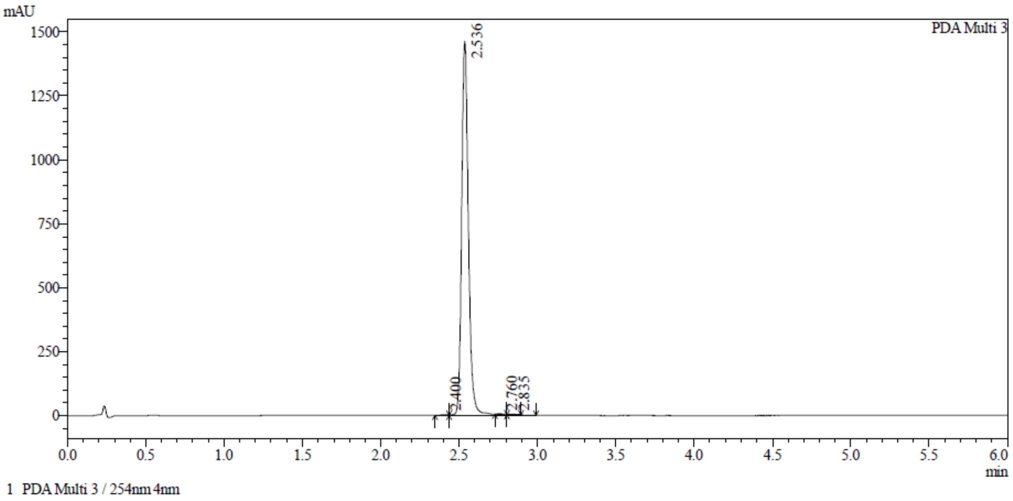

Figure S11: HPLC chromatogram of compound 31. HPLC conditions: 100:0→40:60 water (+0.0375% TFA (v/v)):MeCN (+0.01875% TFA (v/v)).

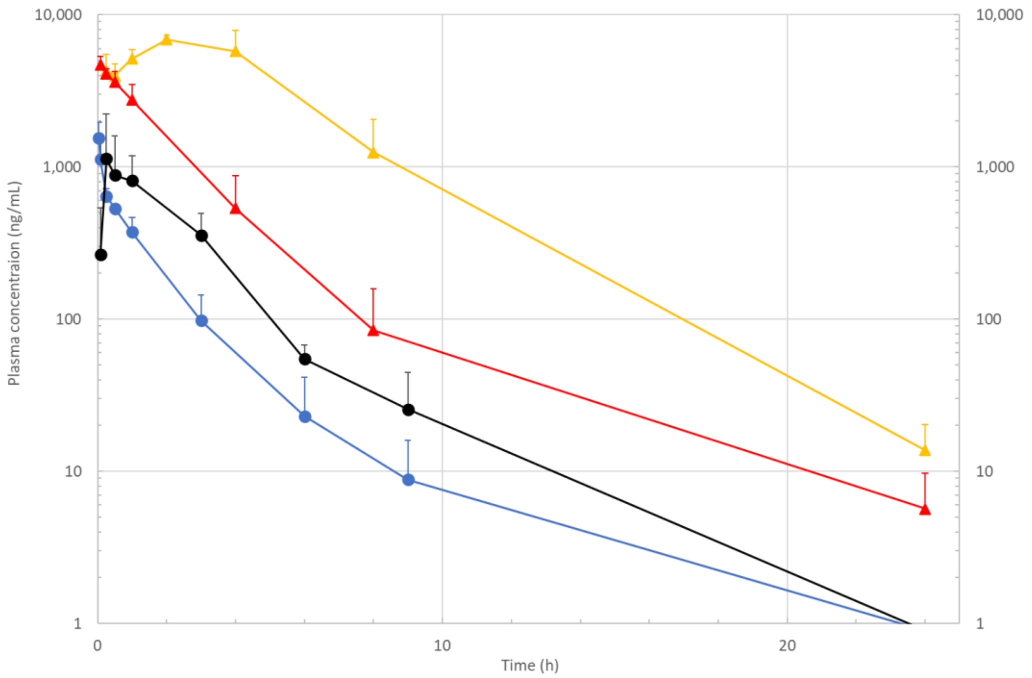

Figure S12: *In vivo* pharmacokinetic properties of compound 23 in rat and dog.

Plasma concentration of **23** following intravenous and oral administration; blue circles: n=2 male Beagle dogs, 1 mg/kg i.v; black circles: n=2 male Beagle dogs, 5 mg/kg p.o; red triangles: n=3 male SD rats, 2 mg/kg i.v.; yellow triangles: n=3 male SD rats, 10 mg/pk p.o.

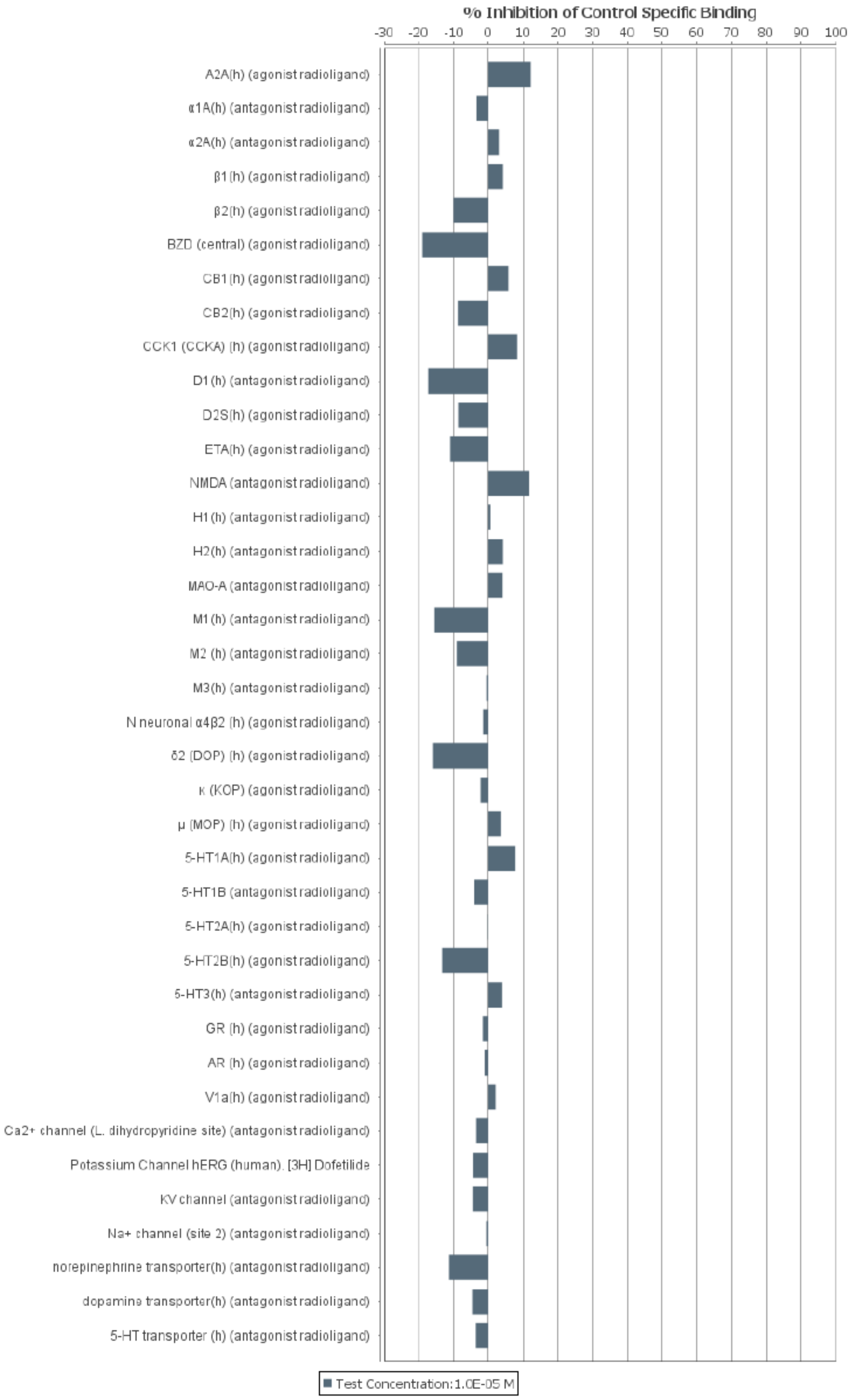

Figure S13: Off target profile of compound 23 (1/3)

Inhibition potential of 23 was tested at 10 μM against 88 targets (Eurofins Cerep Panel).

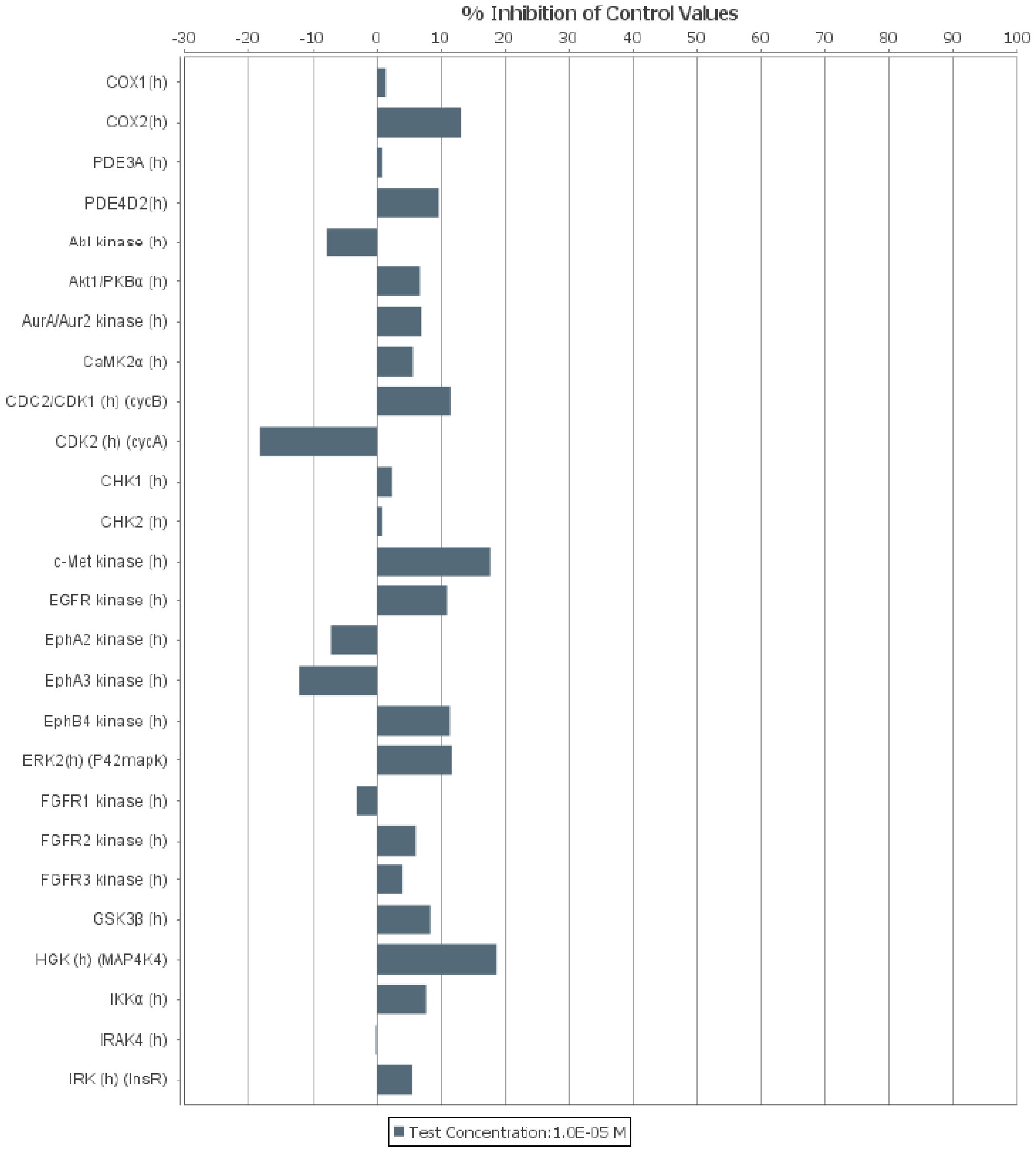

Figure S13: Off target profile of compound 23 (2/3)

Inhibition potential of 23 was tested at 10 µM against 88 targets (Eurofins Cerep Panel).

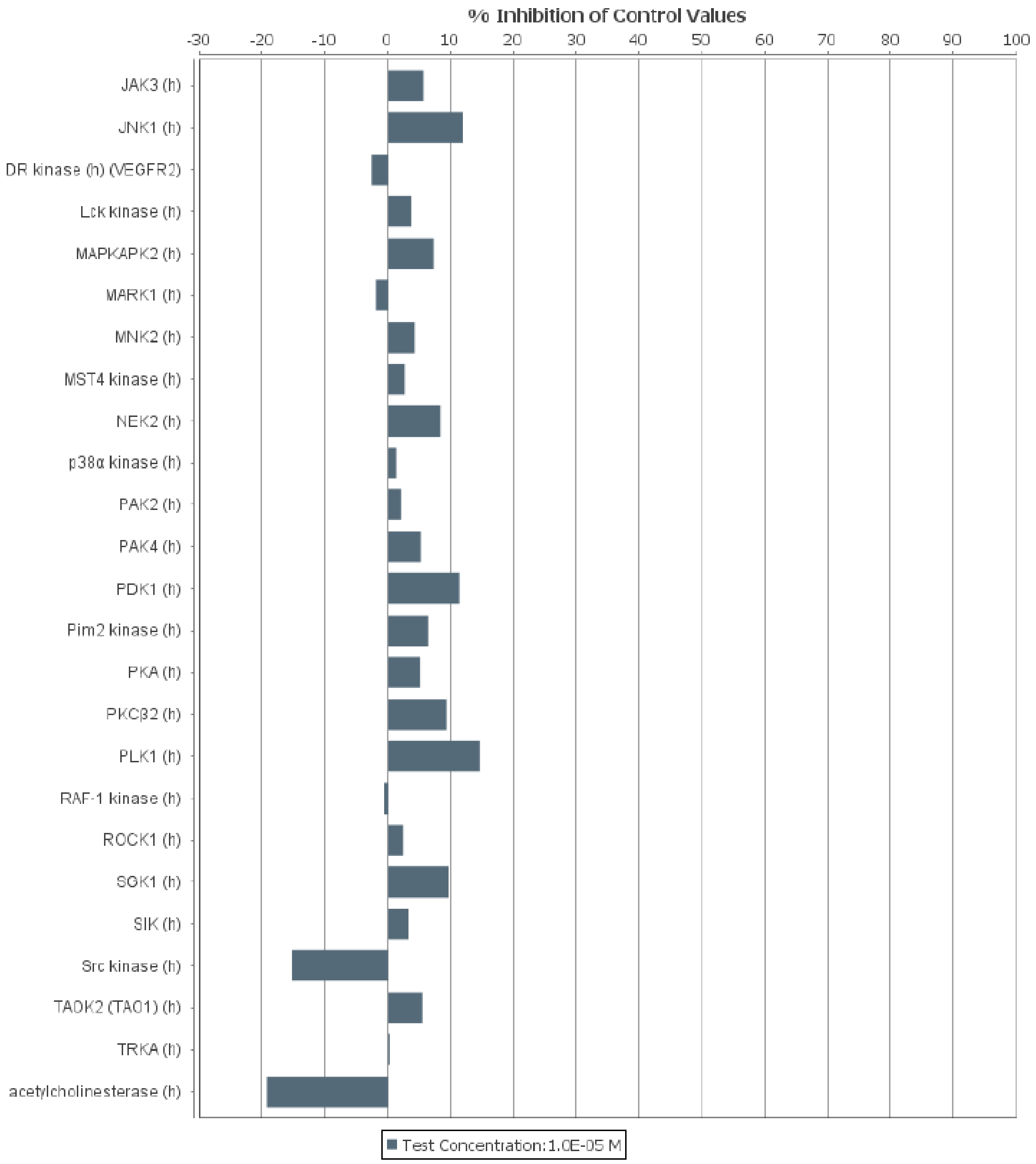

Figure S13: Off target profile of compound 23 (3/3)

Inhibition potential of 23 was tested at 10 µM against 88 targets (Eurofins Cerep Panel)

Table S1: Primers and templates used in CRISPR-Cas9 studies. Y indicates either C/T allowing for a selection between two possible mutations. Conservative changes to assist with the identification of template-guided nucleotide edits are underlined.

**Primer/template**	**Sequence (5' - 3')**
sgRNA	ATACGACTCACTATAGGGCCTCGCGGGACTCGAGCTCGGTTTTAGAGCTAGAAATAGCAAG
G00	AAAAGCACCGACTCGGTGCCACTTTTTCAAGTTGATAACGGACTAGCCTTATTTTAACTTGCTATTTCTAGCTCTAAAAC
His edit template	AGACATCCTGATTGCGGAGAGTACACACGGTATACGCGAGCTCGAGTCCCGCGAGGAGC
Tyr edit template	AGACATCCTGATTGCGGAGAGTACATACGGTATACGCGAGCTCGAGTCCCGCGAGGAGC
Degenerate template	AGACATCCTGATTGCGGAGAGTACA**Y**A**Y**GGTATACGCGAGCTCGAGTCCCGCGAGGAGC

Table S2: EC_{50} values for DNDI-6148 against *L. donovani* clones bearing CRISPR-Cas9 generated edits in *CPSF3*.

Cell lines	EC_{50}, µM[1]	Fold change (relative to WT)
WT	655 ± 40	**-**
Asn[219]His	1979 ± 156	**3.0**
Asn[219]His/Glu[229]Val	2830 ± 139	**5.2**

[1]Each value represents the weighted mean ± SD of three independent experiments.

Table S3: Permeability of DNDI-6148 in MDR1-MDCKII cell monolayer.

Mean P_{app} (10^{-6} cm/s)		Efflux Ratio	Mean total recovery %	
**A to B**	**B to A**		**A to B**	**B to A**
17.11	11.63	0.68	71.06	87.76
